# Supplementary material for: Extracellular vesicle surface display enhances the therapeutic efficacy and safety profile of cancer immunotherapy
Source: Mol Ther. 2024 Jul 20;32(10):3558–79. doi: 10.1016/j.ymthe.2024.07.013 (PMC11489549; doi:10.1016/j.ymthe.2024.07.013)
Supplement: Document S1. Figures S1‒S11, Tables S1 and S2, and supplemental materials and methods [file mmc1.pdf]

YMTHE, Volume *32*

Supplemental Information

Extracellular vesicle surface display enhances the therapeutic efficacy and safety profile of cancer immunotherapy

Migara Kavishka Jayasinghe, Yock Sin Lay, Dawn Xiao Tian Liu, Chang Yu Lee, Chang Gao, Brendon Zhijie Yeo, Faith Yuan Xin How, Rebecca Carissa Prajogo, Dong Van Hoang, Hong Anh Le, Thach Tuan Pham, Boya Peng, Cao Dai Phung, Daniel G. Tenen, and Minh T.N. Le

Supplemental Materials and Methods

Ex vivo tumor cell killing assay

Splenocytes or CD8+ TILs were isolated from the lung homogenates of B16-F10-Luc2 tumor bearing mice using CD8 (TIL) MicroBeads, mouse (Miltenyi Biotec, Germany). Splenocytes or sorted CD8-positive T-cells were subsequently co-cultured with B16-F10-Luc2 cells expressing a firefly luciferase reporter and incubated with desired treatments. Tumor cell death was assayed at 3- and 5-days using luciferase assay (Promega, USA) following the manufacturer's instructions.

RBCEV lyophilization

Lyophilization mixtures of RBCEVs suspended in PBS containing 100 mM trehalose were placed inside a CoolCell® container (Corning, USA) and left to freeze in a -80°C freezer for 4 hours. The frozen samples were then transferred to a FreeZone Plus 4.5 Liter Cascade Benchtop Freeze Dry System (Labconco, USA) for freeze-drying at -84°C, 0.120 mbar. To recover the lyophilised RBCEVs, the vial was reconstituted in deionized water, washed once with PBS by centrifugation at 21,000 g, and resuspended in PBS. The particle count and size distribution were measured using a Zetaview NTA Instrument to determine percentage recovery and size distribution (Particle Metrix, Germany).

EV purification

RBCEVs from mice were purified using similar protocols as outlined in Dang et al.[1] 4TO7 tumor cell-derived EVs were purified as described by Jayasinghe and colleagues.[2]

B16 F10 flank tumor model generation and treatment

8-week old female mice were injected subcutaneously with 0.5 M B15 F10 cells. Tumors were detected by day 3 and mice were treated with 20 µg αCD137 antibody (clone 3H3) either in its free form or conjugated on EVs via intratumoral injection. Flank tumor length and width was measured every 3 days using calipers and the formula $0.5 \times \text{Length} \times \text{Width}^2$ used to calculate tumor volume. At the endpoint of the experiment (day 18), AST and ALT levels were measured as described previously.

Supplemental Tables

Table S1| List of antibodies used for flow cytometry.

Marker/antigen	Conjugate	Clone	CAT#	Laser/Channel	Emission filter
CD86	BUV395	GL1	564199 (BD Bioscience)	UV405	405/30
CD8a	BUV496	53-6.7	750024 (BioLegend)	UV525	525/40
CD25	BUV737	PC61.5	367-0251-82	UV740	740/35
CD3e	BV421	145-2C11	100341 (BioLegend)	V450	450/45
CD11c	BV510	N418	117337 (BioLegend)	V525	525/40
CD170 (Siglec F)	BV605	E50-2440	740388 (BioLegend)	V610	610/20
CD69	BV650	H1.2F3	104541 (BioLegend)	V660	660/10
CD206	BV785	C068C2	141729 (BioLegend)	V780	780/60
CD45	Alexa Fluor 488	30-F11	103122 (BioLegend)	B525	525/40
MHC II	NovaFluor Blue 610-70S	M5/114.15.2	# M024T02B06 (Thermo Fisher Scientific)	B610	610/20
F4/80	BB700	T45-2342	746070 (BD Biosciences)	B690	690/50
Gr-1	PE	RB6-8C5	108408 (BioLegend)	Y585	585/42
CD370	PE	7H11	143504 (BioLegend)	Y585	585/42
CD45	StarBright Yellow 605	YW62.3	MCA1031SBY605 (Bio-Rad)	Y610	610/20
CD11b	PE-Cy5	M1/70	101210 (BioLegend)	Y675	675/30
CD19	NovaFluor Yellow 700	eBio1D3 (1D3)	M004T02Y06 (Thermo Fisher Scientific)	Y710	710/50
CD279 (PD-1)	PE-Cy7	RMP1-30	109110 (BioLegend)	Y780	780/60
Siglec H	PE-Cy7	eBio440c	# 25-0333-82 (eBioscience)	Y780	780/60
CD49b	APC	HMα2	103516 (BioLegend)	R660	660/10

CD161b/c (NK1.1)	APC	PK136	108708 (BioLegend)	R660	660/10
CD103	APC	2E7	121413 (BioLegend)	R660	660/10
CD235ab	APC	HIR2	306608 (BioLegend)	R660	660/10
FoxP3	Alexa Fluor 700	MF-14	126422 (BioLegend)	R712	712/25
CD4	APC-Fire 750	RM4-5	100568 (BioLegend)	R763	763/43
Granzyme B	CF820	QA16A02	372202 (BioLegend/Biotium)	IR840	840/20

Table S2| List of primers used in this study

**Target**	**Primer sequence (5' → 3')**	
Mouse *IL-2*	Forward	GTGCTCCTTGTCAACAGCG
	Reverse	GGGGAGTTTCAGGTTCCTGTA
Mouse *NF-κB*	Forward	GCTGCCAAAGAAGGACACGACA
	Reverse	GGCAGGCTATTGCTCATCACAG
Human *TNF-α*	Forward	CTCTTCTGCCTGCTGCACTTTG
	Reverse	ATGGGCTACAGGCTTGTCACTC
Mouse *GAPDH*	Forward	AGGTCGGTGTGAACGGATTTG
	Reverse	TGTAGACCATGTAGTTGAGGTCA
Human *GAPDH*	Forward	GGAGCGAGATCCCTCCAAAAT
	Reverse	GGCTGTTGTCATACTTCTCATGG
Mouse *IFN-α11*	Forward	GGTCCTGGCACAAATGAGGA
	Reverse	TCCAAGCAGCAGATGAGTCC
Mouse *IFN-α12*	Forward	AAGACTGAGTGAGAAGGAGTGAG
	Reverse	GAGATGCCAGAATTTGAGCAGTG
Mouse *IFN-γ*	Forward	GCCACGGCACAGTCATTGA
	Reverse	TGCTGATGGCCTGATTGTCTT
Mouse *IL-12*	Forward	TGGTTTGCCATCGTTTTGCTG
	Reverse	ACAGGTGAGGTTCACTGTTTCT

Supplemental Data

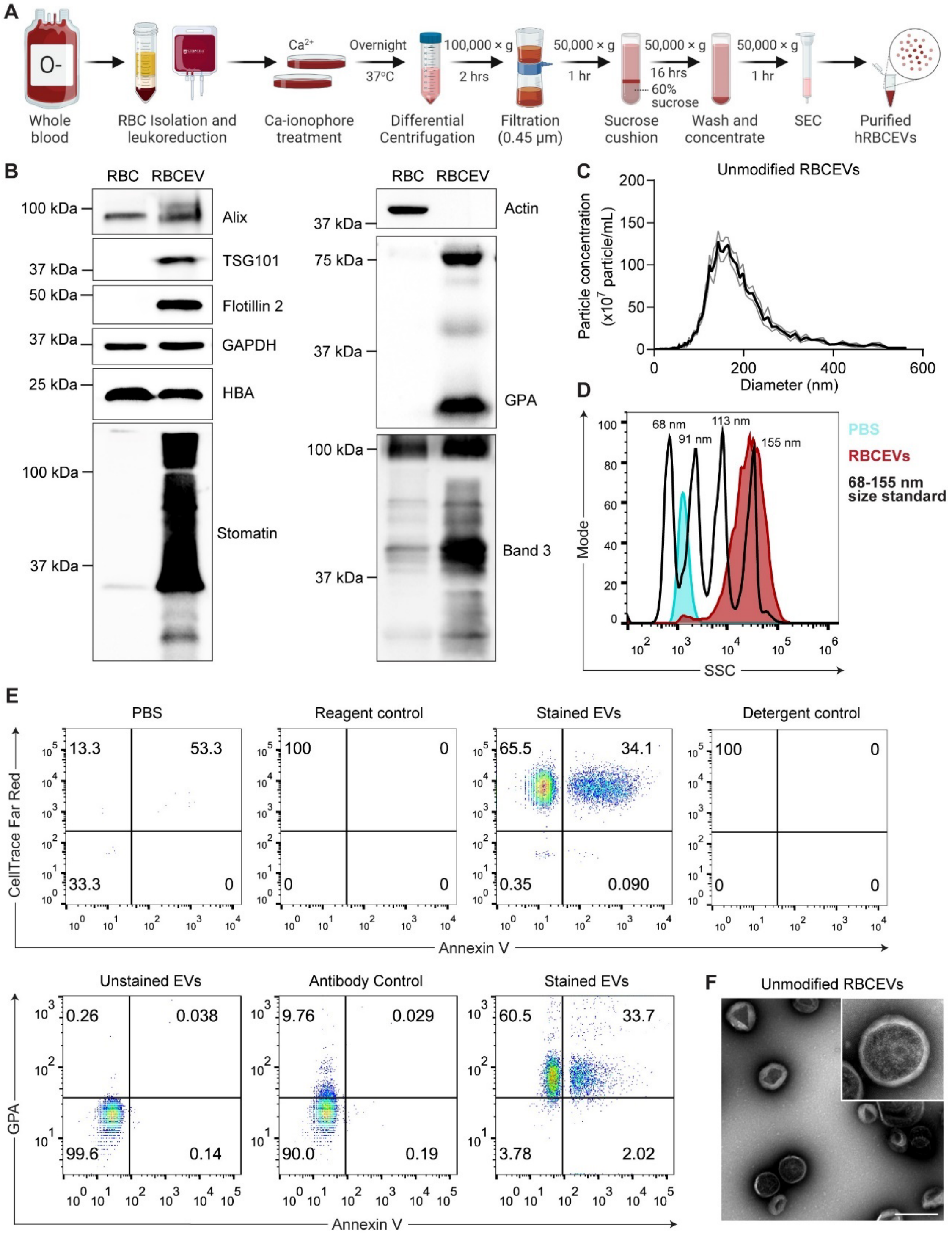

Figure S1| RBCEV purification and characterization. (A) Experimental schema used for the induction, isolation and purification of RBCEVs. (B) Western blot characterization of RBCEVs, comparing relative protein content compared to parental RBCs. (C) Size distribution of unmodified RBCEVs determined using NTA. (D) Gating strategy used to gate out RBCEVs when performing single EV flow cytometric analysis. A buffer control and size standard are included for comparison. Reference diameters for the size standard silica nanospheres are included above each peak. (E) Single EV flow cytometric characterization of RBCEVs, staining for GPA, PS (using Annexin V) and CellTrace Far Red. (F) TEM images of unmodified RBCEVs. Scale bar: 400 nm. NTA: nanoparticle tracking analysis, GPA: Glycophorin A, PS: phosphatidylserine. In (B) molecular weights of protein markers in kDa are shown on the left.

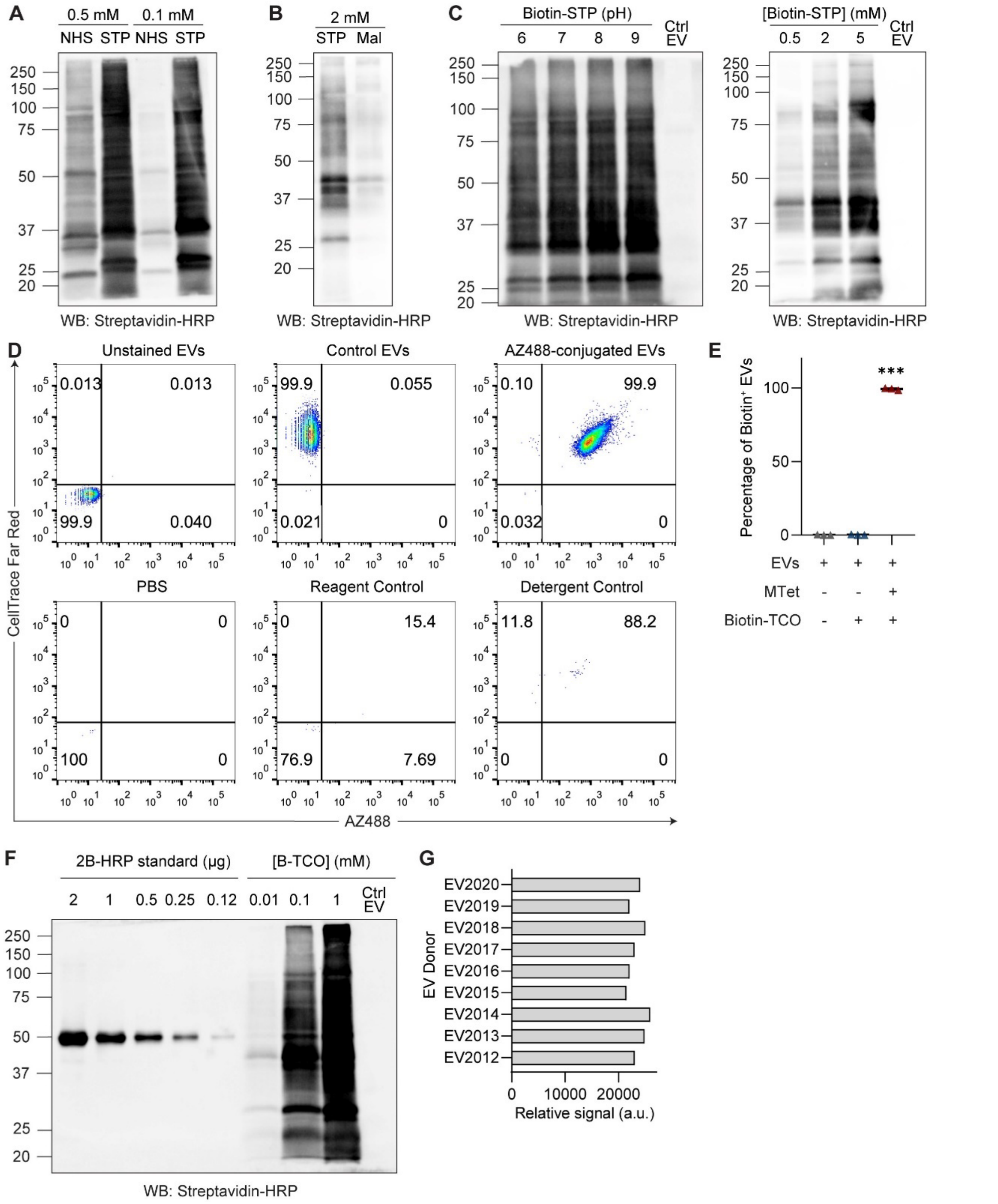

Figure S2| Optimization of ester-activated crosslinking and iEDDA reactions. (A-B) Comparison of relative EV surface modification efficiency of STP ester compared to equivalent concentrations of NHS ester (A) or Maleimide (Mal) (B). Modification efficiency was assessed using reactive esters/maleimide carrying a biotin probe which was detected via western blot. (C) Western blot analysis assessing the relative yield of Biotin-STP reacted with EV surface proteins under different pH or biotin-STP concentration. (D) Flow cytometry dot plots depicting AZ488-TCO conjugation onto MTet-EVs acquired using a NanoFCM system. PBS, reagent controls and detergent controls are included to demonstrate the accuracy of EV detection and all samples were co-stained with CellTrace Far Red to identify EV populations more accurately. (E) Percentage of RBCEVs conjugated with biotin as assessed by single EV flow cytometry. (F) Western blot assessing the conjugation efficiency of increasing concentrations of Biotin-TCO with MTet-EVs. (G) Relative biotin signal of MTet-EVs sourced from separate blood donors that were reacted with Biotin-TCO, quantified using western blot. NHS: N-Hydroxysuccinimide, STP: 4-Sulfo-2,3,5,6-tetrafluorophenyl, TCO: trans-cyclooctene, iEDDA: inverse electron demand Diels–Alder. In (A)-(C) and (F), molecular weights of protein markers in kDa are shown on the left. Error bars represent standard deviation.

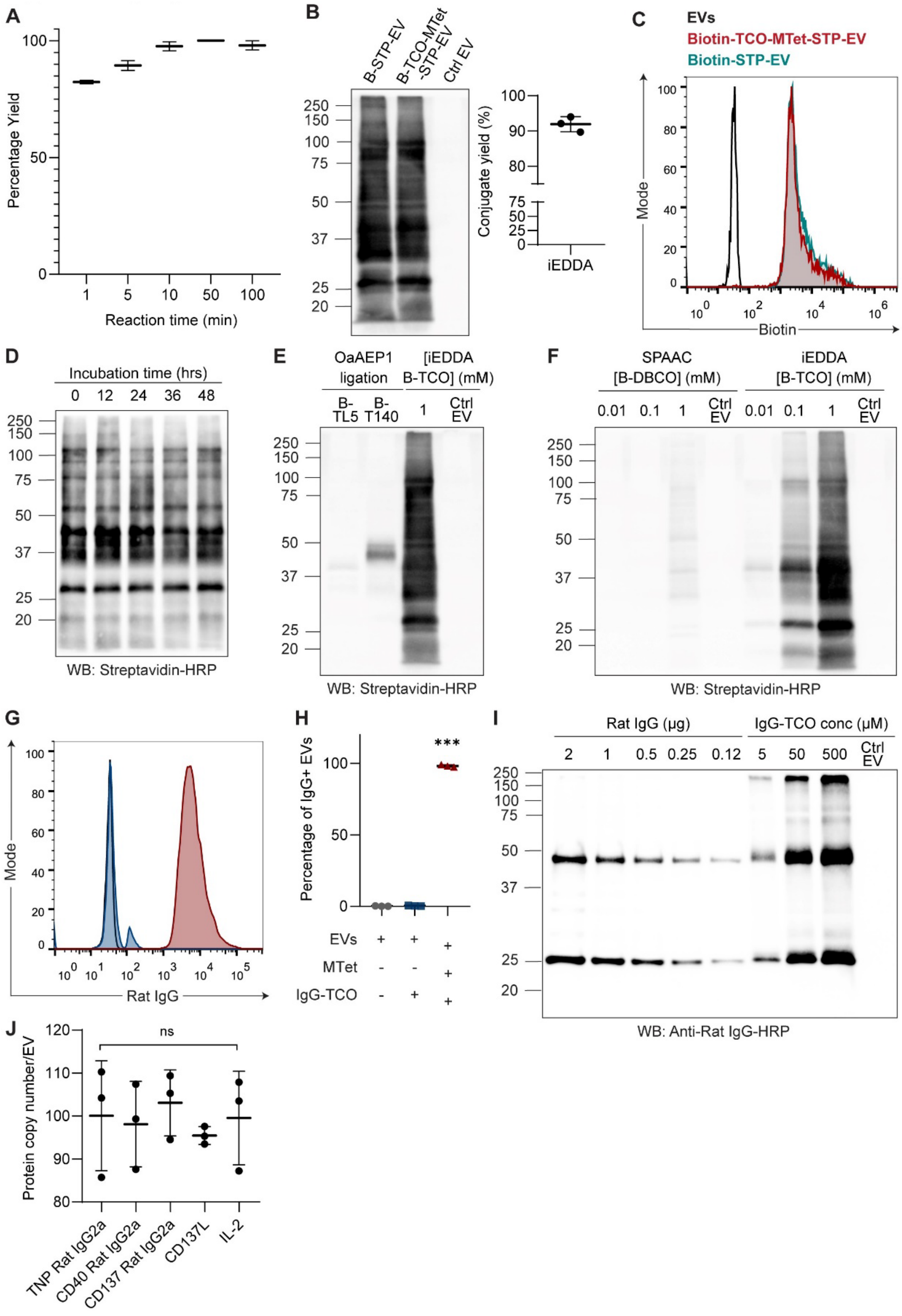

Figure S3| Characterizationon of iEDDA-mediated EV surface functionalization. (A) Percentage yield of the iEDDA reaction over time assessed by the reaction between MTet-EVs with Biotin-TCO. (B) Relative efficiency of iEDDA-mediated conjugation assessed using western blot comparing banding intensity between Biotin-STP-EVs and Biotin-TCO-MTet-EVs. (C) Single EV flow cytometric analysis of Biotin-TCO conjugated EVs as compared to EVs directly reacted with Biotin-STP or control unconjugated EVs. (D) Relative stability of iEDDA-conjugated EVs over a period of 48 hours following incubation in human serum at 37 °C. (E-F) Comparison of iEDDA-mediated conjugation with enzymatic ligation (E) or SPAAC-mediated click chemistry (F). (G) Single EV flow cytometry histogram depicting the conjugation of a TCO-labelled rat IgG onto MTet-labelled EVs. (H) Percentage of EVs conjugated with rat IgG as determined in (G). (I) Western blot analysis assessing the conjugation of IgG onto EVs via iEDDA-

mediated conjugation. Western blot was run under reducing conditions. (J) Copy number of different proteins conjugated on EVs using the same reactions conditions (2.5 µM concentration, 10 minutes). Figures (A), (B), (H) and (J) represent data from 3 individual replicates prepared from separate batches of EVs. MTet: Methyltetrazine, TCO: trans-cyclooctene, iEDDA: inverse electron demand Diels–Alder, SPAAC: strain-promoted azide–alkyne cycloaddition. P value (***$P < 0.001$) was determined using Student's two-tailed t-test. In (B), (D)-(F) and (I) molecular weights of protein markers in kDa are shown on the left. Error bars represent standard deviation.

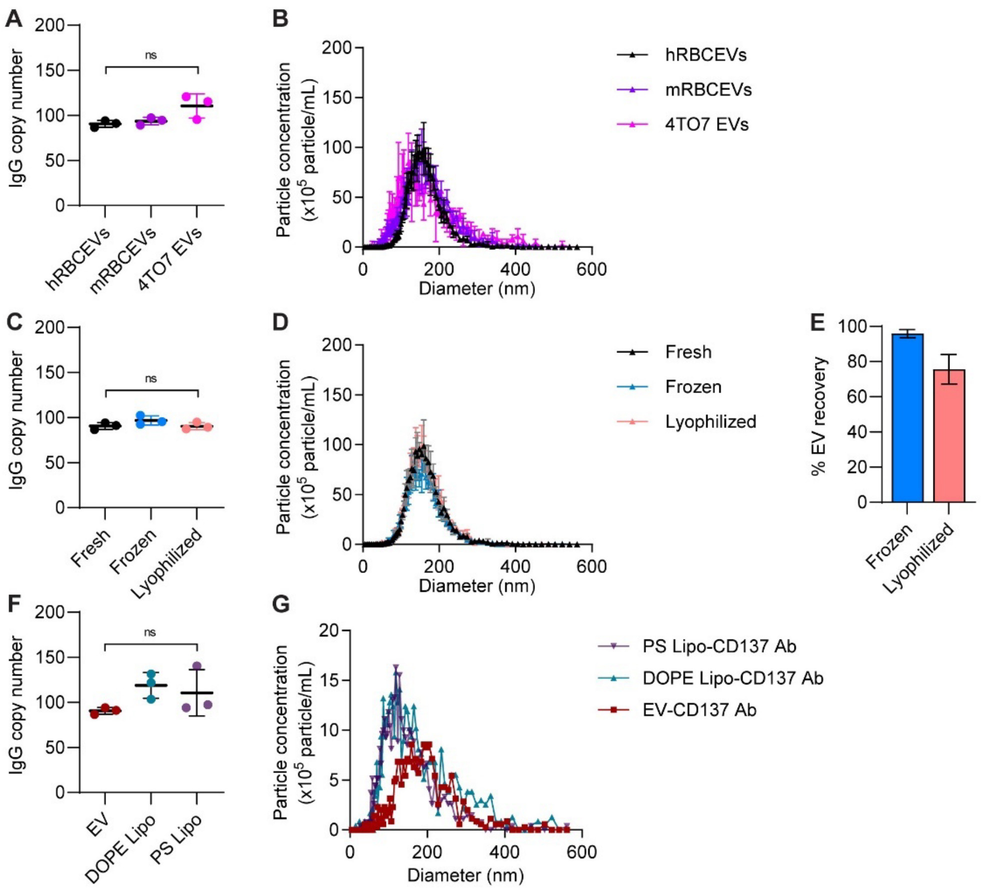

Figure S4| iEDDA-mediated conjugation is stable, versatile and translatable to EVs from other sources and nanoparticles. (A) Copy number of IgGs conjugated on EVs from different sources following the same iEDDA-mediated conjugation protocol (2.5 µM concentration, 10 minutes). (B) Size distribution of each of the EVs from (A) following iEDDA-mediated conjugation. (C) Copy number of IgGs remaining on IgG-conjugated EVs following storage under different conditions. (D) Size distribution of conjugated EVs following storage and recovery for each of the storage conditions in (C). (E) Percentage EV recovery for EVs frozen at -80 °C or lyophilized. (F) Copy number of CD137 antibody present on a single EV or liposome following iEDDA-mediated conjugation at an IgG-TCO concentration of 2.5 µM. (G) NTA analysis displaying the size distribution of DOPE and PS liposomes following conjugation to CD137 antibody using iEDDA chemistry. The size distribution of EVs conjugated with CD137 antibody is overlaid for comparison. Figures (A), (C) and (F) represent data from 3 individual replicates prepared from separate batches of EVs. TCO: trans-cyclooctene, iEDDA: inverse electron demand Diels–Alder, DOPE: dioleoylphosphatidylethanolamine, PS: phosphatidylserine. P value (***$P < 0.001$) was determined using Student's two-tailed t-test. Error bars represent standard deviation.

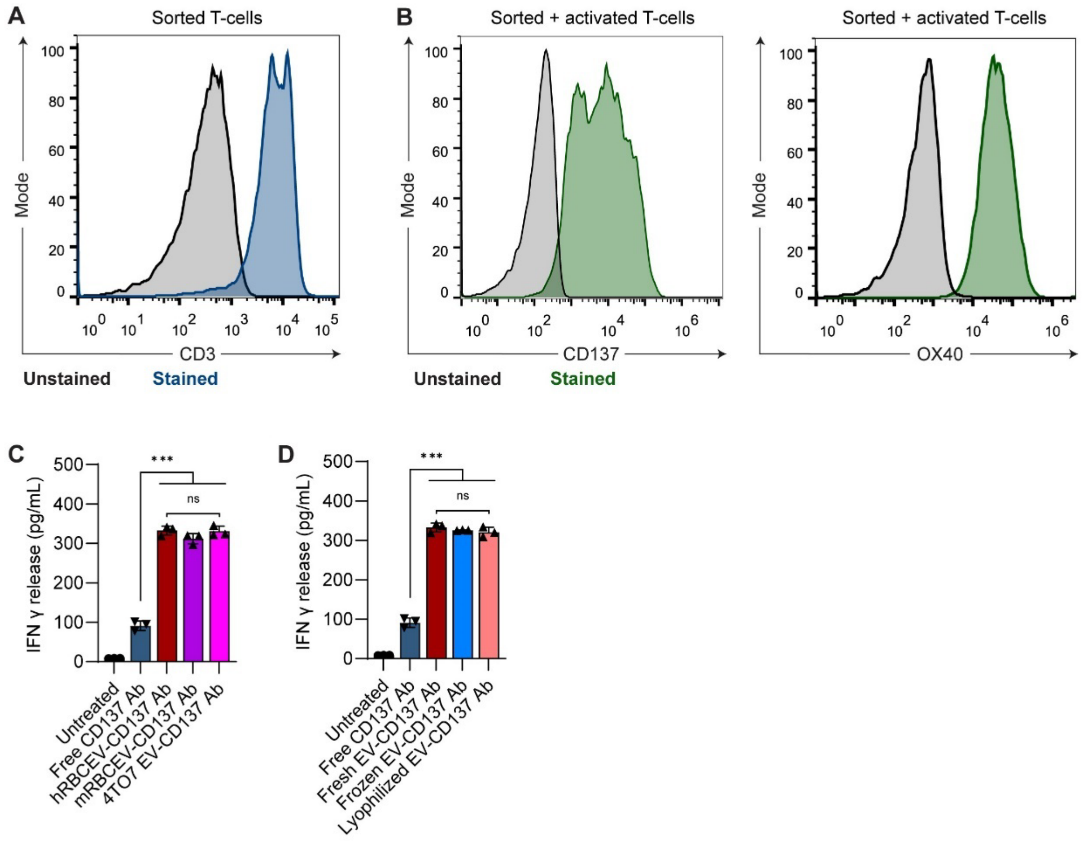

Figure S5| Characterization of *in vitro* functionality of ligand conjugated EVs. (A) Flow cytometric analysis of T-cells after negative selection for T-cells using an untouched mouse T-cells kit. (B) Expression of CD137 and OX40 on T-cells following 3 days of stimulation with mouse T-Activator CD3/CD28 Dynabeads™. (C) Relative stimulation efficiency of EVs from different sources conjugated with an agonistic CD137 antibody assessed via IFN-γ release. (D) IFN-γ release from activated T-cells following treatment with human RBCEVs conjugated with agonistic CD137 antibody that were stored under different conditions. hRBCEV: human red blood cell-derived EVs, mRBCEV: mouse red blood cell-derived EVs. Student's two-tailed t-test: ns – not significant, *P < 0.05, ***P < 0.001. Error bars represent standard deviation.

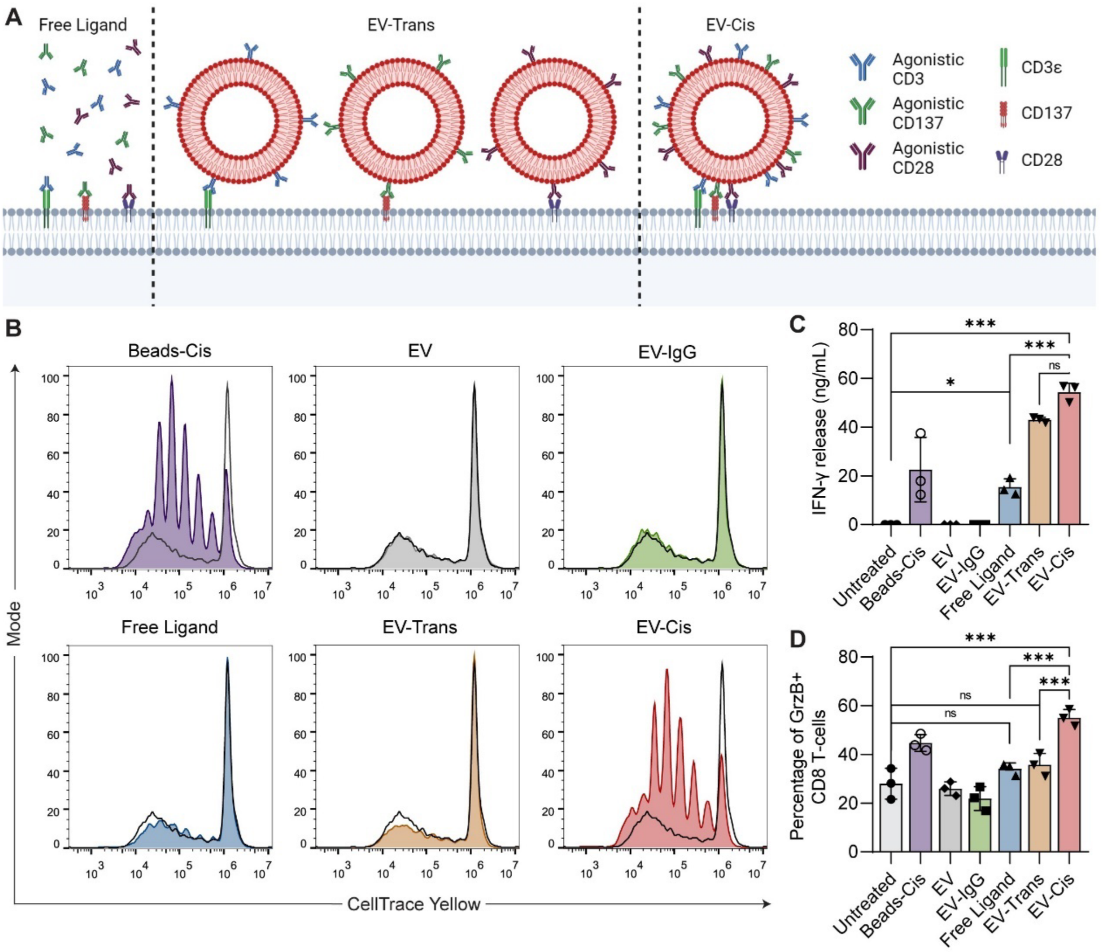

Figure S6| Display of multiple complementary ligands in *cis* is superior to *trans* display. (A) Schematic illustrating the experimental design utilized to compare the relative signalling efficiency of *cis* versus *trans* EVs as compared to free antibodies. Unactivated T-cells were used for this experiment. (B) CellTrace proliferation assay conducted on T-cells on day 3 following stimulation with each treatment. The unstimulated cells are overlaid with each histogram in black for comparison. (C) IFN-γ ELISA of the supernatant of T-cells 6 days post-treatment. (D) Percentage of Granzyme B positive CD8+ T-cells in each treatment condition on day 6 assessed using intracellular flow cytometry. For (C)-(D), each replicate was performed using EVs from individual donors and T-cells from different mice. Student's two-tailed t-test: ns – not significant, *P < 0.05, ***P < 0.001. Error bars represent standard deviation.

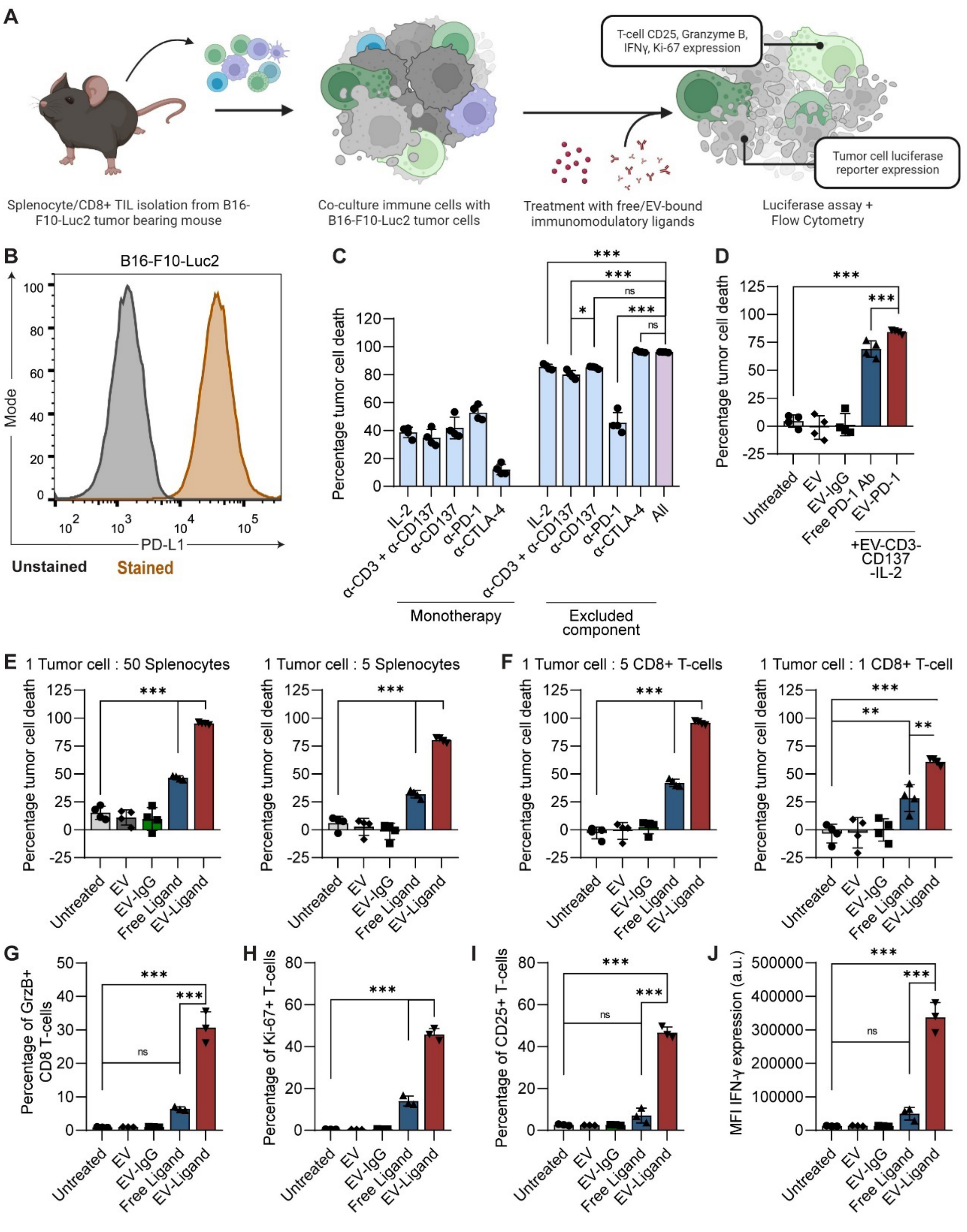

Figure S7| Multifunctional immunomodulatory EVs display superior tumor cell suppression compared to free ligands *in vitro*. (A) Design of the *ex vivo* tumor cell killing assay. Immune cells were isolated from tumor bearing mice, followed by co-cultures with B16-F10-Luc2 cells expressing a firefly luciferase reporter. (B) Expression of PD-L1 in B16-F10-Luc2 cells determined using flow cytometric analysis. (C) Evaluation of tumor cell killing efficacy of 5 candidate immunotherapeutics either alone or excluded from a combined cocktail. Efficacy was assessed via percentage tumor cell death obtained via luciferase assay. (D) *Ex vivo* tumor cell killing assay assessing the relative efficacy of PD-1 in its free form versus upon conjugation to EVs. Both treatments with the PD-1 antibody also included EVs conjugated with αCD3, αCD137 and mIL-2. (E-F) Percentage tumor cell death following co-culture with different ratios of either splenocytes (E) or sorted CD8 T-cells (F) at the stated ratios. (G-J) Expression of granzyme B in CD8-positive T-cells (G), Ki-67 in total T-cells (H), CD25 in total T-cells (I) or IFN-γ in total T-cells (J) following co-culture and treatment. For (C) and (D), each replicate was performed using EVs from individual donors. For (E)-(J), each replicate was performed using EVs from individual donors and T-cells isolated from separate mice. Student's two-tailed t-test: ns – not significant, *P < 0.05, ***P < 0.001. Error bars represent standard deviation.

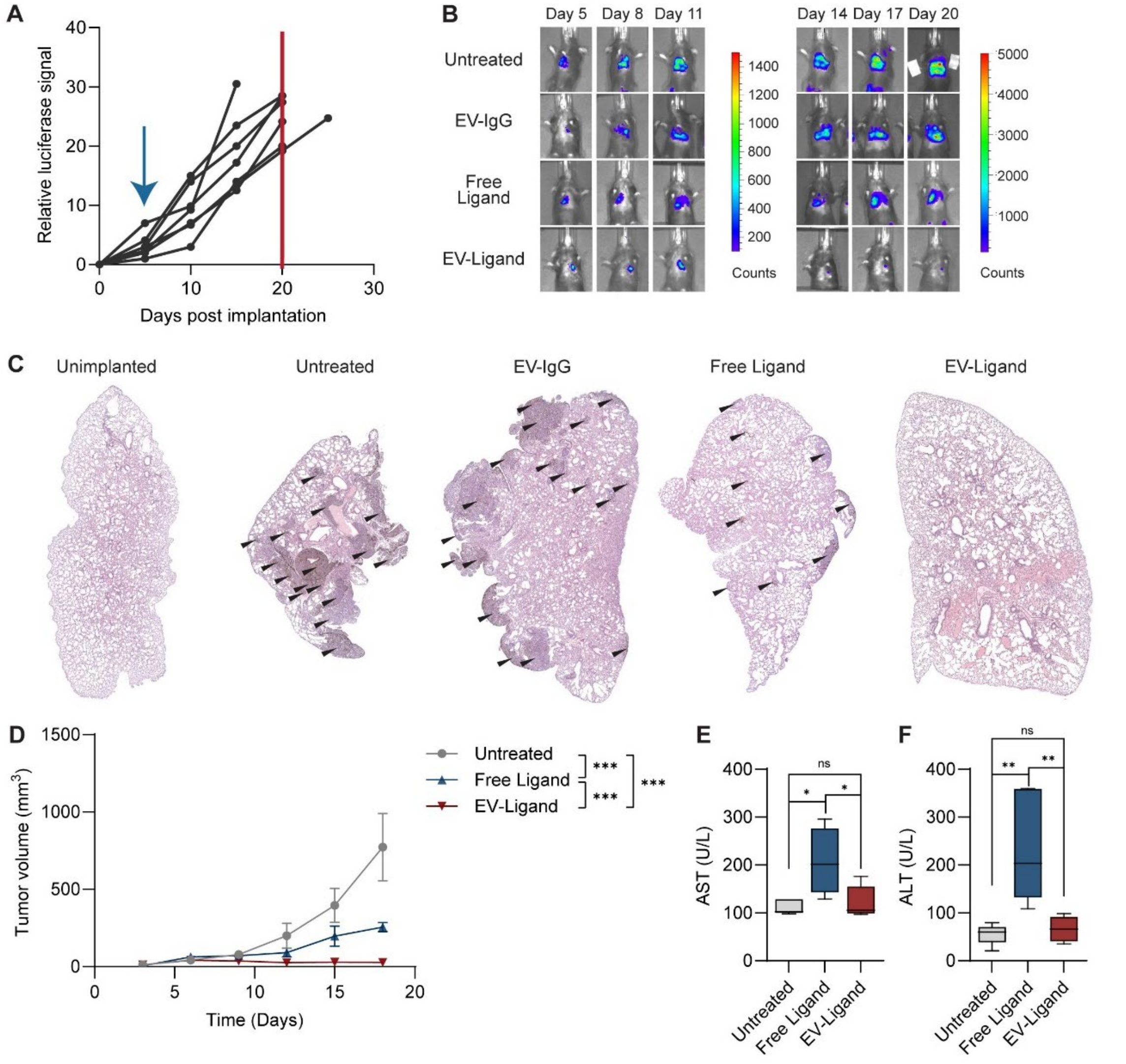

Figure S8| EV-Ligand treatments show superior *in vivo* tumor suppression in multiple models of melanoma. (A) Relative luciferase signals from mice injected intravenously with 0.5M B16-F10-Luc2 cells monitored over a period of 25 days. The blue arrow indicates the timepoint selected for starting treatment administration and the red line indicates the end point of the study, at which most mice succumbed to tumor burden. (B) Representative IVIS images of mice from each treatment group over the 20-day treatment period. (C) Representative H&E images of complete lung sections from tumor bearing mice 20 days post-treatment. Significant tumor modules are indicated with arrows. (D) Tumor volume of B16 F10 flank tumors over a treatment period of 18 days. Treatments were administered at 3 days intervals starting on day 3. (E-F) AST (E) and ALT (F) levels of B16 F10 flank tumor bearing mice across each treatment group at the endpoint of the treatment (day 18). For the B16 F10 flank tumor treatment in (D)-(F), n=3 mice and the treatment consisted of agonistic CD137 antibody either in its free form or conjugated on EVs. Student's two-tailed t-test: ns – not significant, *P < 0.05, ***P < 0.001. Error bars represent standard deviation.

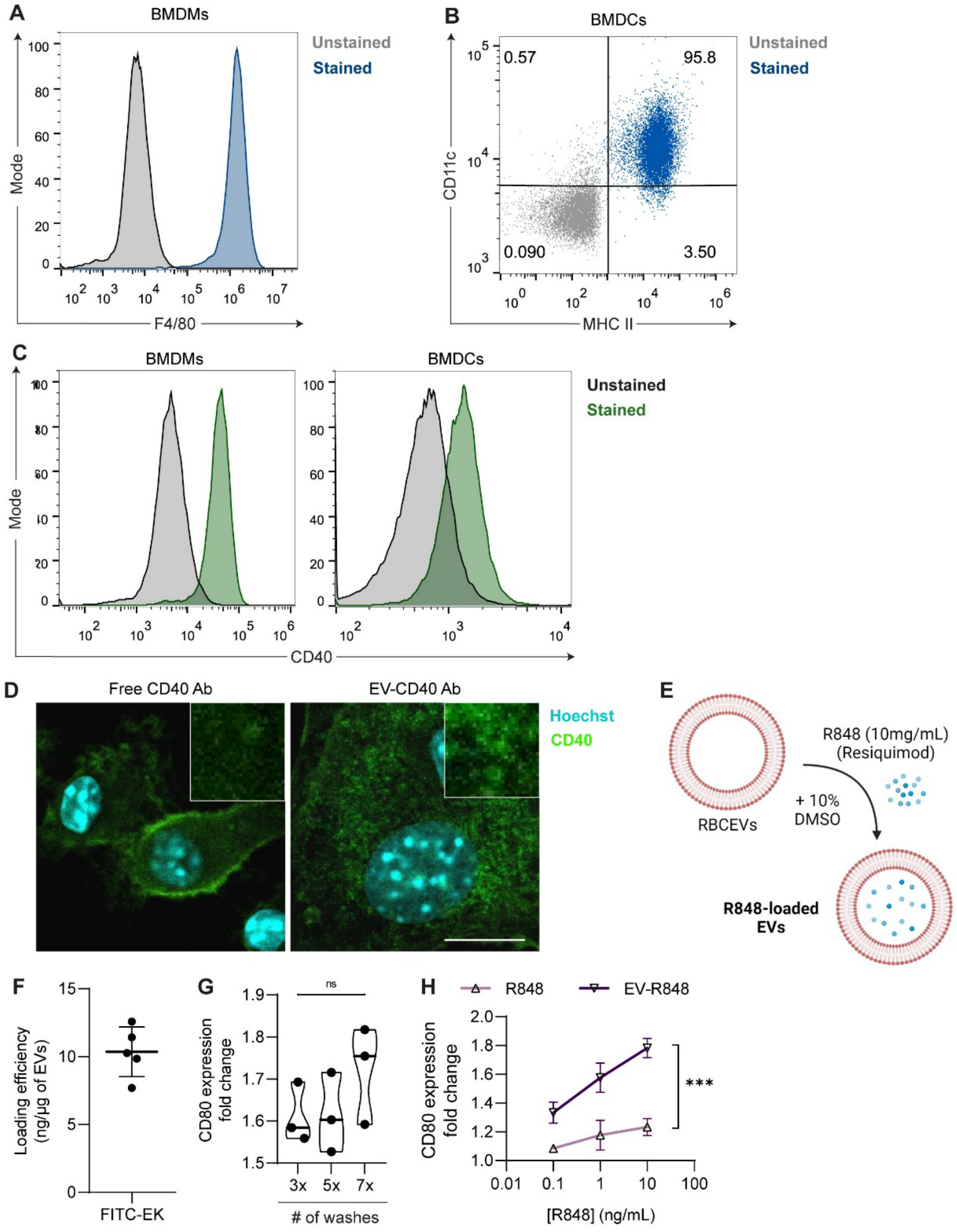

Figure S9| APC differentiation and R848 loading. (A) Flow cytometric analysis of F4/80 in murine BMDMs following differentiation and enrichment. (B) Flow cytometric analysis of DC markers CD11c and MHC II following differentiation and negative selection of murine BMDCs. (C) Flow cytometry histograms depicting CD40 expression in murine BMDMs and BMDCs. (D) High resolution image demonstrating distinct receptor clustering of CD40 in BMDMs following stimulation with free CD40 agonistic antibodies or EV-conjugated CD40 antibodies. Scale bar: 10 µm. (E) Protocol for loading R848 into EVs. EVs were co-incubated with R848 in the presence of 10% DMSO. (F) Quantity of R848 loaded per µg of EVs determined using a fluorescent probe. (G) CD80 expression fold change in RAW264.7 cells following incubation with R848-loaded EVs that were washed 3, 5 and 7 times respectively. (H) Relative increase in CD80 expression in RAW264.7 cells following treatment with increasing doses of free R848 or EV-loaded R848. For (F)-(H), each replicate was performed using EVs from individual donors. Student's two-tailed t-test: ns – not significant, *P < 0.05, ***P < 0.001. Error bars represent standard deviation.

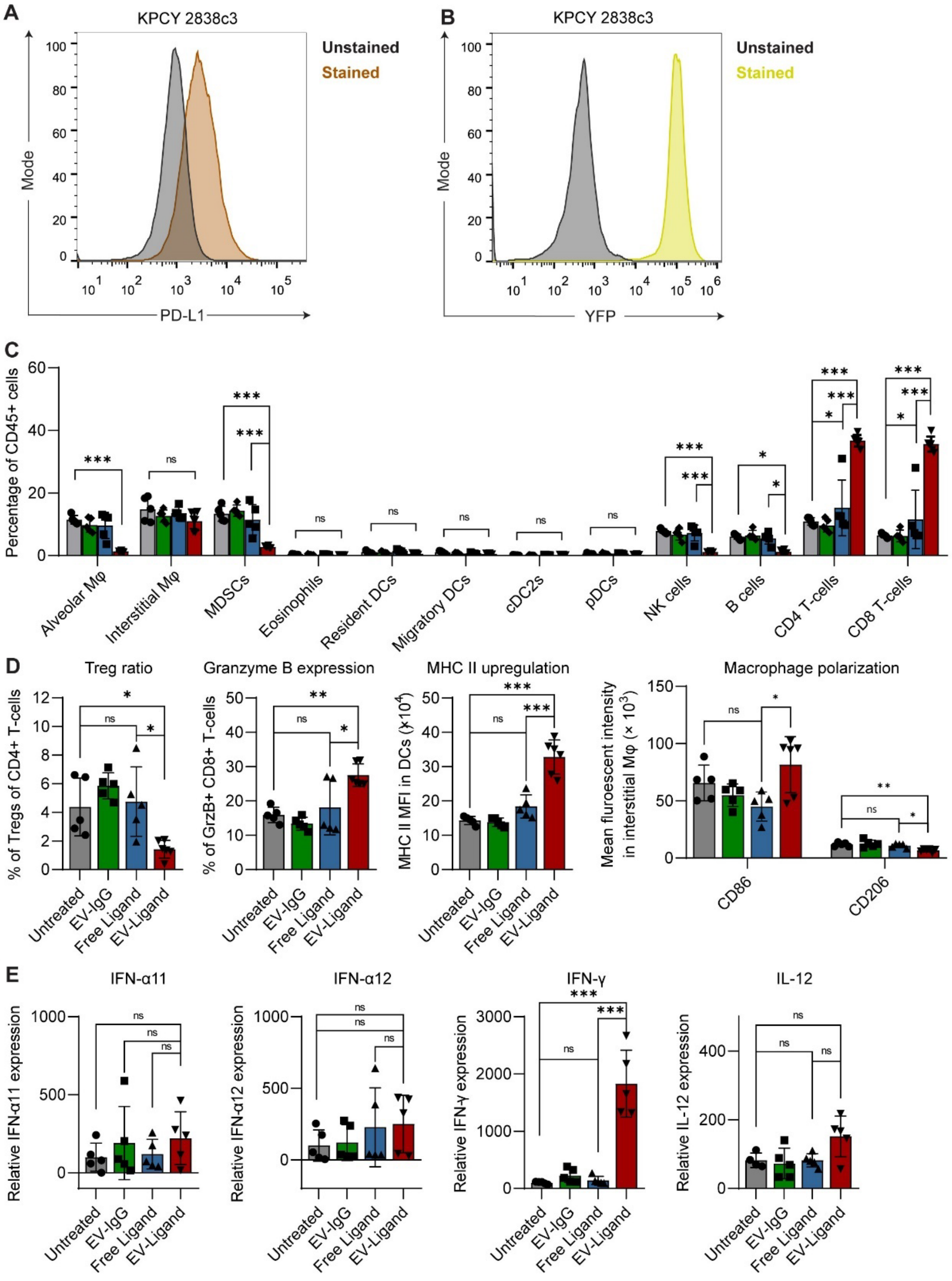

Figure S10| PDAC tumor immune composition analysis following treatment. (A) PD-L1 expression in KPCY 2838c3 cells determined using flow cytometry. (B) Expression of the fluorescent YFP reporter in KPCY 2838c3 cells determined using flow cytometry. (C) Composition of major immune cell subsets in the tumor-bearing lung 30 days post-treatment represented as a percentage of all CD45-positive cells. (D) Analysis of key immune cell subsets relevant to the generation of effective anti-cancer immune responses. (E) RT-qPCR analysis of the expression of various pro-inflammatory cytokines in lung extracts at the endpoint of the treatment. For (C)-(E) data was obtained from 5-6 mice. Student's two-tailed t-test: ns – not significant, *P < 0.05, ***P < 0.001. Error bars represent standard deviation.

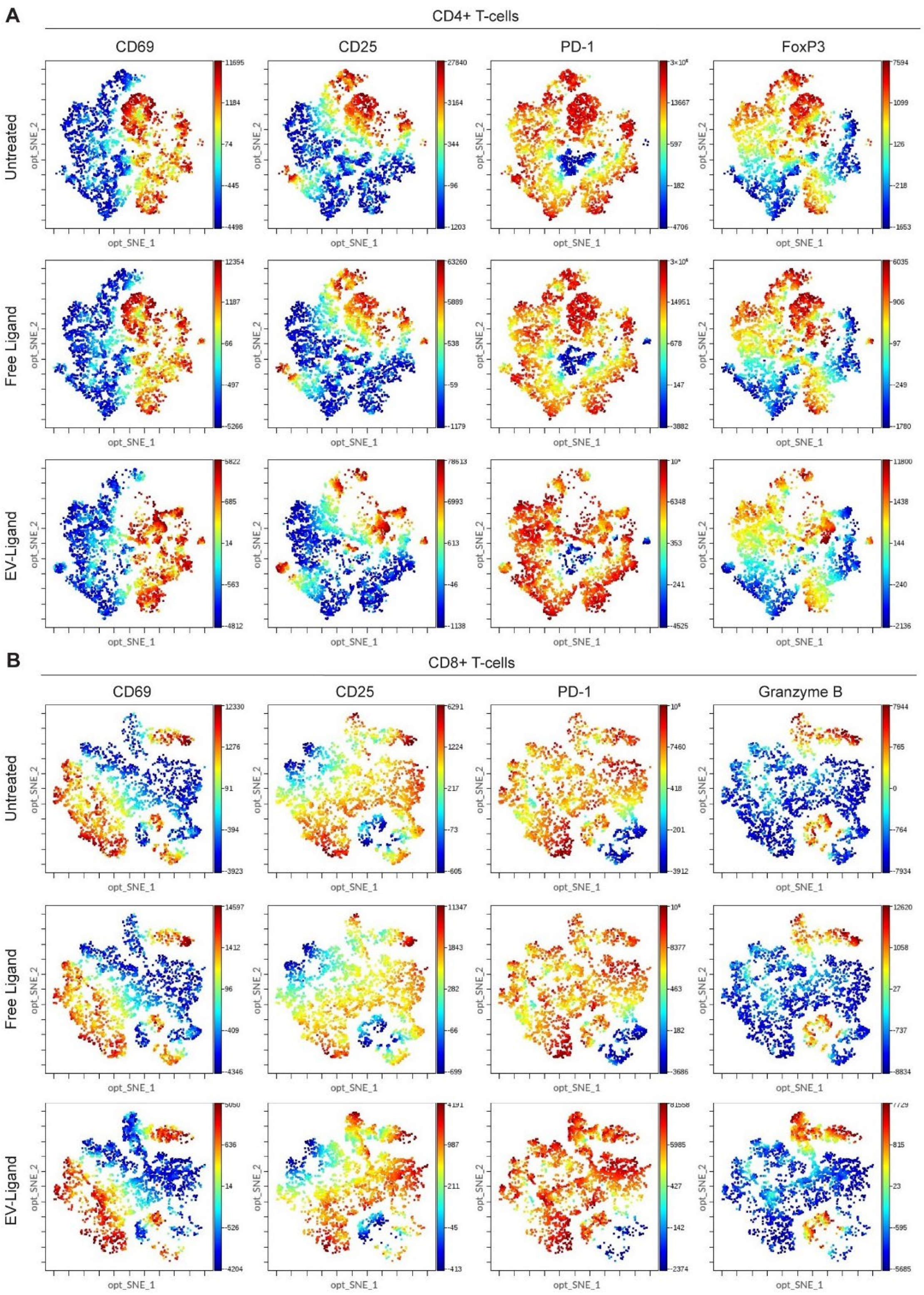

Figure S11| EV-Ligand treatments effectively remodel tumor infiltrating lymphocytes. (A) Detailed expression profiles of CD69, CD25, PD-1 and FoxP3 in CD4+ T-cells following administration with each of the indicated treatments. (B) In depth analysis of CD69, CD25, PD-1 and Granzyme B expression in CD8+ T-cells. For (A)-(B), T-cell subsets were subjected to a opt-SNE dimensionality reduction algorithm and the respective markers are indicated on the z-axis.

1 Dang, X. T. T. *et al.* Dendritic cell-targeted delivery of antigens using extracellular vesicles for anti-cancer immunotherapy. *Cell Proliferation* **n/a**, e13622 (2024). https://doi.org:https://doi.org/10.1111/cpr.13622

2 Jayasinghe, M. K. *et al.* Red Blood Cell-Derived Extracellular Vesicles Display Endogenous Antiviral Effects and Enhance the Efficacy of Antiviral Oligonucleotide Therapy. *ACS Nano* **17**, 21639-21661 (2023). https://doi.org:10.1021/acsnano.3c06803
