# Supplementary material for: Extracellular vesicle surface display enhances the therapeutic efficacy and safety profile of cancer immunotherapy
Source: Mol Ther. 2024 Jul 20;32(10):3558–79. doi: 10.1016/j.ymthe.2024.07.013 (PMC11489549; doi:10.1016/j.ymthe.2024.07.013)
Supplement: Document S2. Article plus supplemental information [file mmc2.pdf]

Molecular Therapy
Original Article

Extracellular vesicle surface display enhances the therapeutic efficacy and safety profile of cancer immunotherapy

Migara Kavishka Jayasinghe,[1,2,3] Yock Sin Lay,[1,2] Dawn Xiao Tian Liu,[1,2] Chang Yu Lee,[1,2] Chang Gao,[1,2] Brendon Zhijie Yeo,[1,2] Faith Yuan Xin How,[1,2] Rebecca Carissa Prajogo,[1,2] Dong Van Hoang,[1,2] Hong Anh Le,[1,2] Thach Tuan Pham,[1,2] Boya Peng,[1,2] Cao Dai Phung,[1,2] Daniel G. Tenen,[3,4] and Minh T.N. Le[1,2,5]

[1]Institute for Digital Medicine and Department of Pharmacology, Yong Loo Lin School of Medicine, National University of Singapore, Singapore 117600, Singapore; [2]Department of Surgery, Yong Loo Lin School of Medicine, National University of Singapore, Singapore 117600, Singapore; [3]Cancer Science Institute of Singapore, National University of Singapore, Singapore 117599, Singapore; [4]Harvard Stem Cell Institute, Harvard Medical School, Boston, MA 02138, USA; [5]Institute of Molecular and Cell Biology, A*STAR, Singapore 138673, Singapore

Immunotherapy has emerged as a mainstay in cancer therapy, yet its efficacy is constrained by the risk of immune-related adverse events. In this study, we present a nanoparticle-based delivery system that enhances the therapeutic efficacy of immunomodulatory ligands while concurrently limiting systemic toxicity. We demonstrate that extracellular vesicles (EVs), lipid bilayer enclosed particles released by cells, can be efficiently engineered via inverse electron demand Diels-Alder (iEDDA)-mediated conjugation to display multiple immunomodulatory ligands on their surface. Display of immunomodulatory ligands on the EV surface conferred substantial enhancements in signaling efficacy, particularly for tumor necrosis factor receptor superfamily (TNFRSF) agonists, where the EV surface display served as an alternative FcγR-independent approach to induce ligand multimerization and efficient receptor crosslinking. EVs displaying a complementary combination of immunotherapeutic ligands were able to shift the tumor immune milieu toward an anti-tumorigenic phenotype and significantly suppress tumor burden and increase survival in multiple models of metastatic cancer to a greater extent than an equivalent dose of free ligands. In summary, we present an EV-based delivery platform for cancer immunotherapeutic ligands that facilitates superior anti-tumor responses at significantly lower doses with fewer side effects than is possible with conventional delivery approaches.

INTRODUCTION

The advent of immunotherapy stands out as a significant milestone in the field of cancer treatment. The last decade has seen the emergence of multiple forms of cancer immunotherapy, such as immune checkpoint inhibitors, agonistic antibodies, and recombinant cytokines. While the efficacy of cancer immunotherapeutics has been widely acclaimed, it has become increasingly apparent that there are certain limitations to their effectiveness. Notably, immunotherapeutics can induce adverse side effects or immune-related adverse events that include, but are not limited to, autoimmune responses, liver toxicity, and cytokine release syndrome.[1,2] A key reason for this is the non-specific nature of many immunotherapeutics, which can lead to unintended immune activation throughout the body, causing toxicity to healthy cells outside the tumor microenvironment. Thus, the potential for off-target toxicity poses a serious dose-limiting constraint on the anti-tumor efficacy of cancer immunotherapy.

To address this issue, the development of novel delivery strategies and improved immunomodulators with higher specificity have been an area of active research. A promising approach in this regard involves the use of delivery vectors that alter the biodistribution of immunomodulatory ligands in the body, thereby limiting their off-target effects. This was demonstrated by Lewis et al., who demonstrated that extracellular vesicles (EVs) engineered to display interleukin (IL)-12 on their surface were superior to an equivalent dose of free IL-12 in terms of efficacy and toxicity.[3] Of note, both these effects were attributed to the altered biodistribution of the EVs, as the EVs limited the exposure of IL-12 to the immediate tumor microenvironment upon local administration. This resulted in a high localized concentration of IL-12 in the tumor, improving immune activation in the tumor microenvironment while concurrently minimizing systemic exposure. Since then, there have been a multitude of additional studies that utilized liposomes, engineered EVs, and other nanoparticles in an effort to improve the delivery of various immunotherapeutics to tumor sites.[4–7] While these strategies have shown promise in enhancing

Received 14 April 2024; accepted 17 July 2024;
https://doi.org/10.1016/j.ymthe.2024.07.013.

Correspondence: Minh T.N. Le, Department of Pharmacology, Yong Loo Lin School of Medicine, National University of Singapore, 16 Medical Drive, Singapore 117600, Singapore.
E-mail: phcltnm@nus.edu.sg

 © 2024 The Authors.
Published by Elsevier Inc. on behalf of The American Society of Gene and Cell Therapy.
This is an open access article under the CC BY license (http://creativecommons.org/licenses/by/4.0/).

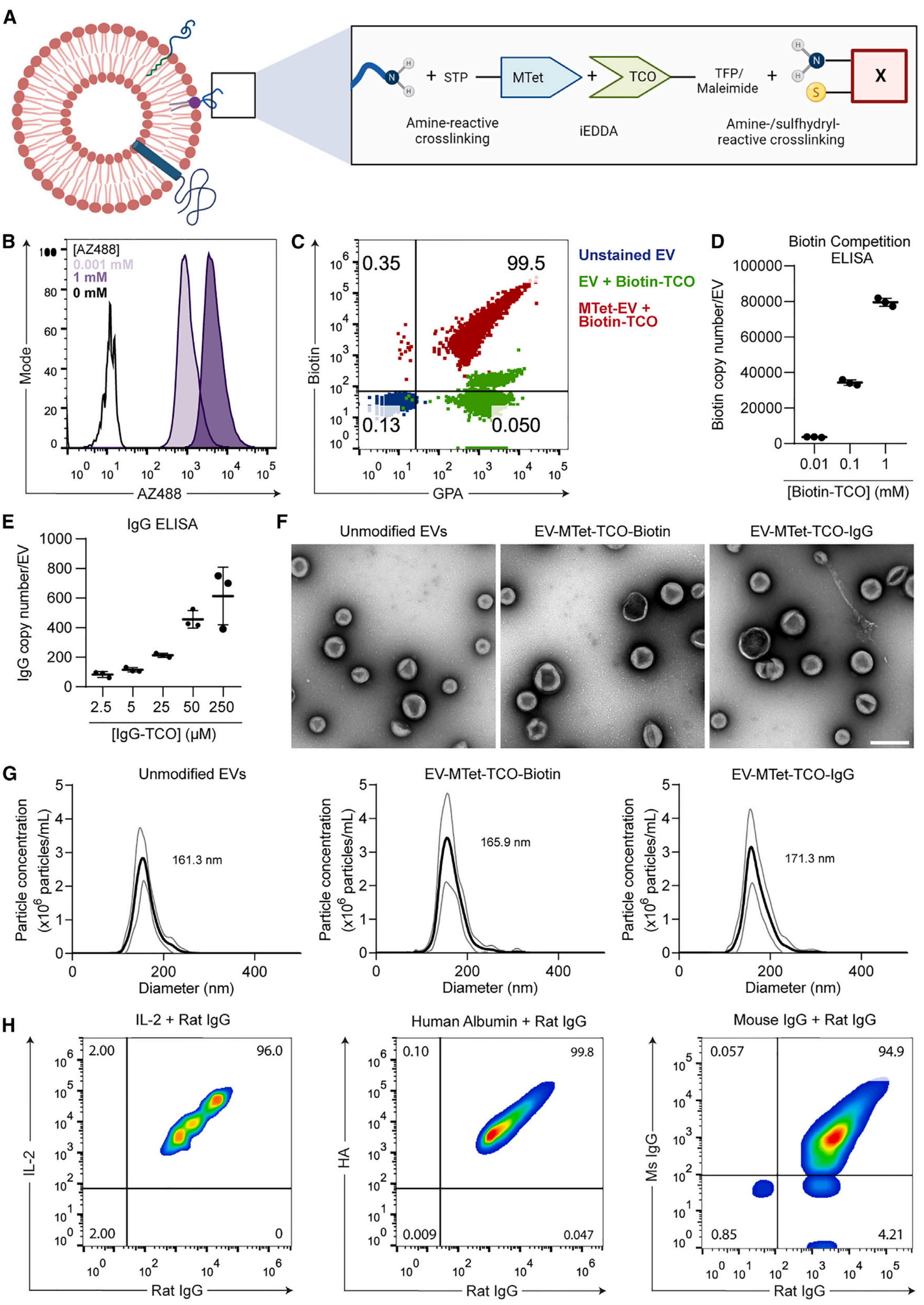

(legend on next page)

the safety profile of immunotherapeutics or enhancing their therapeutic efficacy, they often have trouble reconciling these two aspects. Indeed, more potent ligands have the potential to cause enhanced toxicity, while strategies to enhance safety compromise the efficacy of immune activation in the tumor. Thus, a more efficacious and versatile approach to decrease systemic toxicity of immunotherapeutics without compromising their anti-tumor effects is required.

In this study, we aimed to develop an improved vector capable of enhancing both the efficacy and safety profile of immunotherapeutics. We hypothesized that displaying immune-stimulatory ligands on the surface of EVs at high copy number could mediate ligand multimerization, allowing the EV to mimic cell-cell interactions akin to an immune synapse, that in turn could induce receptor crosslinking. In this way, engineered EVs could achieve FcγRIIB-independent crosslinking of receptors for more efficient signaling. Furthermore, given that locally administered EVs would remain in the immediate tumor microenvironment, we hypothesized that these immune-stimulatory effects would be localized to the vicinity of the tumor and thereby limit systemic toxicity. To maintain a clinically translatable profile, we utilized red blood cell-derived EVs (RBCEVs) as nanocarriers instead of synthetic formulations or immortalized cell-derived EVs given their higher levels of biocompatibility, safety, and scability.[8–15]

RBCEVs were functionalized with immunotherapeutic ligands using a novel EV surface engineering approach based on inverse electron demand Diels-Alder (iEDDA) chemistry that facilitated versatile and ultrafast conjugation of multiple ligands at high efficiency. Ligand functionalized EV treatments consistently outperformed equivalent doses of free ligands in stimulating immune cells both *in vitro* and *in vivo*. Remarkably, EV-mediated delivery of immunotherapeutic ligands was also superior to delivery via liposomal formulations. EV-associated ligands maintained a high local concentration in the tumor microenvironment, facilitating potent local immune activation and anti-tumor immunity at lower doses than conventionally required, while concurrently minimizing systemic toxicity. Ligand functionalized EVs efficiently modulated the tumor immune milieu toward a pro-inflammatory phenotype to generate effective anti-tumor immune responses in two models of lung metastatic cancer, resulting in improved overall survival and the development of tumor-specific immune memory, rendering mice immune to secondary tumor challenge.

RESULTS

EVs can be efficiently surface functionalized using iEDDA-mediated conjugation

RBCEVs were purified from human red blood cells as outlined in Figure S1A. Western blot analysis of RBCEVs revealed the enrichment of EV-specific markers (Alix, TSG101), lipid raft-associated proteins (Flotillin 2, Stomatin), and specific RBC membrane proteins (Glycophorin A, Band 3), along with the concurrent depletion of cytoskeletal actin (Figure S1B). Nanoparticle tracking analysis (NTA) showed that RBCEVs had an average diameter of ∼160 nm (Figure S1C), while single EV flow cytometric analysis using a NanoFCM system revealed the expression of glycophorin A (GPA, an RBC-specific protein), and phosphatidylserine (PS) exposure on RBCEVs (Figures S1D and S1E). When observed under a transmission electron microscope (TEM), the EVs displayed a characteristic cup-shaped morphology following negative staining (Figure S1F).

Methyltetrazine (MTet) click handles were installed on EVs for iEDDA reactions using 4-Sulfo-2,3,5,6-tetrafluorophenyl (STP) esters, a more efficient alternative to N-hydroxysuccinimide (NHS) esters or sulfhydryl reactive crosslinkers (Figures S2A–S2C). Figure 1A outlines the approach utilized for iEDDA-mediated conjugation of MTet-EVs with *trans*-cyclooctene (TCO)-labeled proteins. The conjugation efficiency of this reaction was verified by reacting MTet-EVs with a fluorescent AZ488-labeled TCO probe. Single EV flow cytometry revealed that concentrations as low as 0.001 mM AZ488-TCO resulted in highly efficient conjugation, evidenced by the significant rightward shift of the EV population (Figure 1B). This aligns with the reaction kinetics reported for iEDDA reactions that result in high yields even at very low substrate concentrations. The accuracy of single EV detection was confirmed via the inclusion of reagent and detergent controls as per MISEV 2018 guidelines (Figure S2D). Similar results were observed when MTet-EVs were reacted with a Biotin-TCO probe (Figures 1C and S2E). In this experiment, EVs were co-stained with GPA to confirm that the biotin-positive events were contributed by RBCEVs, as indicated by the identification of a clearly discernible double-positive EV population. Biotin-TCO-conjugated EVs were also analyzed using western blot to confirm the covalent nature of conjugation, with increasing concentrations of Biotin-TCO displaying increased yields of covalently conjugated biotin per EV (Figure S2F).

Quantification of biotin content per EV using competitive ELISA indicated a concentration-dependent increase in copy number, with

Figure 1. iEDDA-mediated conjugation facilitates efficient EV surface functionalization

(A) Schematic depicting iEDDA-mediated EV surface functionalization. (B) Single EV flow cytometric analysis assessing the conjugation efficiency of varying concentrations of fluorescent AZ488-TCO with MTet-EVs. The concentration of AZ488-TCO used is indicated in the top left corner of the histogram. (C) Single EV flow cytometric analysis assessing Biotin-TCO conjugation on MTet-EVs. EVs were co-stained with anti-GPA antibody to accurately gate RBCEVs. Percentages on the gated plot are shown for MTet-EV + Biotin-TCO. (D) Quantification of Biotin-TCO probes conjugated per EV determined using competition ELISA. (E) Copy number of IgG conjugated per EV, determined using a total Rat IgG ELISA kit. (F) Representative TEM micrographs of EVs before and after iEDDA-mediated conjugation. Scale bar, 300 nm. (G) Size distribution profiles of unmodified and iEDDA-conjugated EVs determined using NTA. The mean diameter is noted on each histogram. (H) Single EV flow cytometric analysis of MTet-EVs conjugated with pairs of TCO-labeled proteins. Figures (D) and (E) represent data from three individual replicates prepared from separate batches of EVs. For (D) and (E), the concentration in μM depicts the concentration of TCO-labeled biotin/IgG incubated with MTet-EVs for the iEDDA reaction. MTet: methyltetrazine, TCO: *trans*-cyclooctene, iEDDA: inverse electron demand Diels-Alder. In (C) molecular weights of protein markers in kDa are shown on the left. Error bars represent standard deviation.

up to 80,000 copies of a biotin probe conjugated on a single EV at the highest concentration of Biotin-TCO used (Figure 1D). The yield was consistent across EVs from different donors (Figure S2G). Remarkably, we observed that approximately 80% of the maximum yield was obtained following 1 min of incubation, while the reaction reached completion within 10 min, as assessed using western blot (Figure S3A). At the endpoint of the iEDDA reaction, we determined that 90% of the MTet molecules on the EV surface had successfully reacted with a TCO probe (Figures S3B and S3C). Western blot analysis of biotin-conjugated EVs revealed that incubation in human plasma had no significant detrimental effects on the stability of iEDDA-mediated conjugation (Figure S3D). Notably, the iEDDA-mediated conjugation approach exhibited significantly higher yields compared with existing post-isolation methods currently in use for EV surface engineering, including OaAEP1-mediated enzymatic ligation and strain promoted alkyne-azide cycloaddition (SPAAC) (Figures S3E and S3F).

After validating the functionality of our bioconjugation approach using probes, we attempted to conjugate larger proteins using the optimized protocol developed above. Single EV flow cytometric analysis and western blotting revealed efficient and covalent conjugation of TCO-labeled immunoglobulin (Ig)Gs onto MTet-EVs, confirming the translatability of this conjugation approach to larger, functional proteins (Figures S3H and S3I). Using a total Rat IgG ELISA kit, we observed that IgG concentrations as low as 2.5 μM resulted in over 80 copies of IgG per EV (Figure 1E). Increasing TCO-IgG concentrations resulted in increasing yields, with the copy number approaching saturation at ∼450–600 copies of IgG per EV. Further investigation revealed that different proteins with the same degree of labeling conjugated under the same reaction conditions resulted in comparable copy numbers of each protein conjugated per EV, regardless of the size or nature of the protein (Figure S3J). TEM and NTA analysis showed that iEDDA-mediated conjugation did not compromise the EVs' structure or significantly alter EV size or induce aggregation (Figures 1F and 1G). To determine if it was possible to conjugate multiple ligands on the EV surface, we mixed equimolar quantities of TCO-labeled rat IgG with TCO-labeled IL-2, human albumin, or mouse IgG and reacted them with MTet-EVs. Using single EV flow cytometry, we detected up to ∼95%–96% of double-positive EVs for each combination of ligands, confirming that multiple ligands can be conjugated on a single EV with high efficiency (Figure 1H).

We also investigated if the iEDDA-mediated conjugation approach was translatable to EVs from multiple sources such as human RBCEVs, mouse RBCEVs, and 4TO7 tumor cell-derived EVs. Our data demonstrated that EVs from all the sources tested had similar copy numbers of IgG per EV and displayed comparable size distributions (Figures S4A and S4B). Our data also revealed that EVs engineered via iEDDA-mediated conjugation were stable under a range of commonly used storage conditions including freezing at −80°C (with 4% trehalose) and lyophilization, displaying no loss of the conjugated proteins or any significant changes in their overall size distribution (Figures S4C and S4D). Remarkably we were able to recover over 95% of EVs following freezing and over 75% of EVs following lyophilization, suggesting that therapeutics based on this engineered EV platform would be able to be readily stored long-term and transported without the need for dedicated storage or shipping facilities (Figure S4E). For our downstream experiments we wanted to perform side-by-side comparisons to liposomal delivery of ligands. Thus, we prepared engineered liposomes displaying CD137 agonistic antibodies using a similar iEDDA-based protocol as we used for the EVs. Interestingly, comparable copy numbers were obtained when dioleoylphosphatidylethanolamine (DOPE) and phosphatidylserine (PS) liposomes were conjugated with IgG using a similar protocol (Figures S4F and S4G).

Display of immunotherapeutic ligands on the EV surface enhances signaling efficacy

We hypothesized that conjugating immune-stimulatory ligands on the EV surface could facilitate EV-mediated multimerization of ligands and induce receptor crosslinking to achieve stronger agonistic effects compared with stimulation with an equivalent dose of free ligands. This is particularly applicable for members of the tumor necrosis factor receptor superfamily (TNFRSF) members that are generally dependent on receptor crosslinking for effective signaling, with antibody-based agonists typically requiring FcγRIIB expression on accessory cells. TNFRSF members and their cognate ligands play a major role in lymphoid tissue development and homeostasis and numerous members, including CD137, CD40, and OX40, are key targets for cancer immunotherapy. To test this hypothesis, we used an agonistic CD137 antibody capable of binding to and stimulating CD137, a TNFRSF member expressed on activated T cells (Figures 2A, S5A, and S5B). CD137 antibody-conjugated EVs (EV-CD137 Ab), but not isotype antibody-conjugated EVs (EV-IgG) significantly increased the binding of EVs to activated T cells, confirming that antibodies conjugated on the EV surface maintained their binding affinity to their target antigens (Figures 2B–2D).

Next, we compared the relative efficacy of EV-conjugated CD137 to an equivalent quantity of free CD137 antibody at inducing interferon gamma (IFN-γ) release from activated T cells following 24 h of stimulation. Treatment with EV-CD137 Ab resulted in an ∼3-fold increase in IFN-γ release as compared with free CD137 antibody treatment, suggesting that conjugating immunomodulatory ligands on the EV surface can enhance their therapeutic efficacy (Figure 2E). These EV-mediated enhancements in signaling were also observed with EV-CD137 Ab treatments prepared using EVs from different sources (Figure S5C). Importantly, freezing down EVs at −80°C or lyophilization had no detrimental effect on the performance of these ligand-conjugated EVs (Figure S5D). Surface display of CD137 antibody on either DOPE or PS liposomes also resulted in a significant enhancement in IFN-γ release, but the increment was lower than that observed with EVs, despite both formulations having similar copy numbers of antibody (Figures 2E and S4E). This indicates that EVs are better suited for stimulating immune cells via the use of surface-displayed ligands as compared with synthetic liposomes.

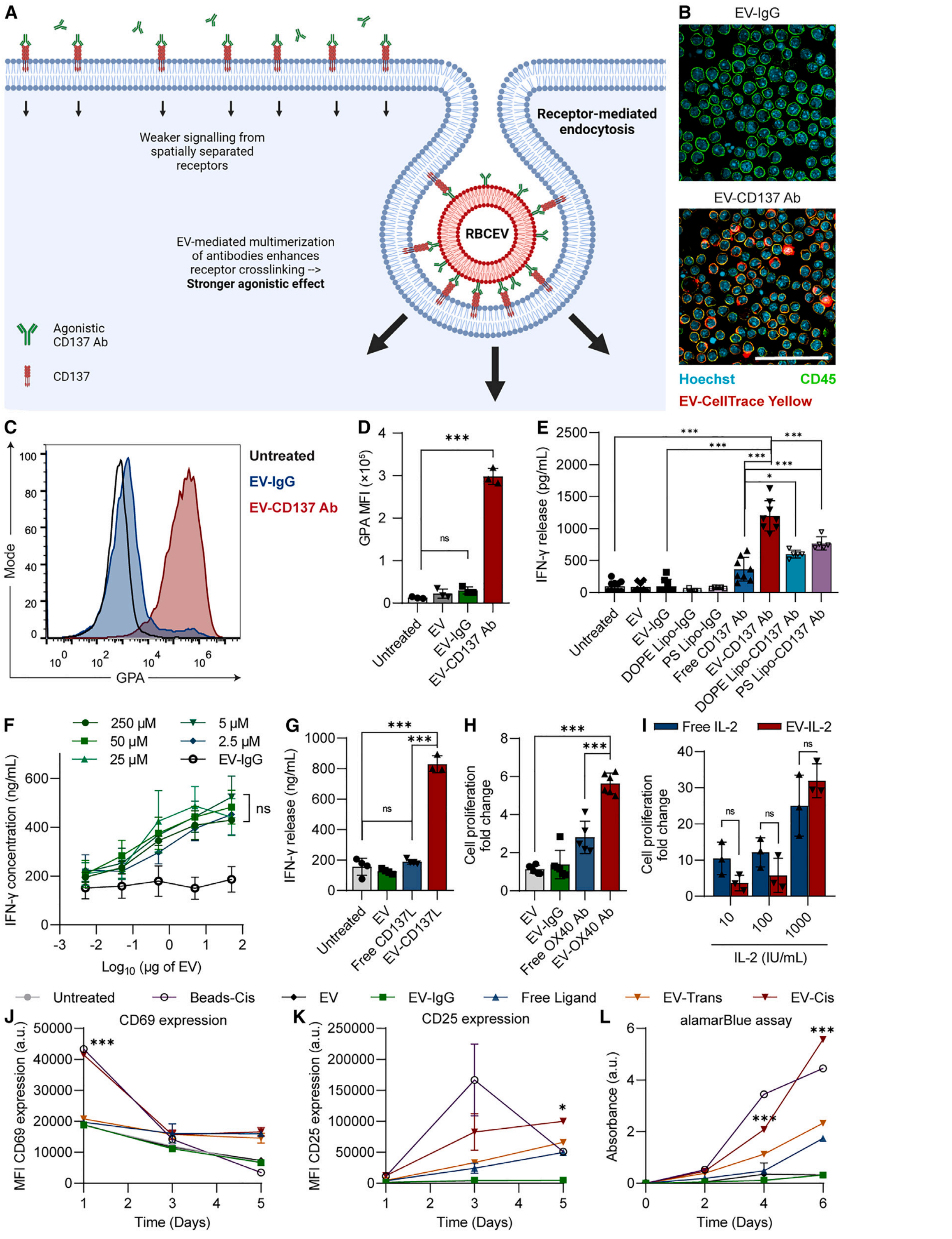

(legend on next page)

Dose-response analysis of agonistic CD137 antibody-conjugated EVs revealed a sigmoidal dose-response curve, with EV concentrations as low as 50 ng/mL resulting in detectable levels of IFN-γ release (Figure 2F). At EV concentrations approaching 50 μg/mL we observed a plateau in IFN-γ release. Remarkably, the antibody copy number obtained at the lowest TCO-antibody concentration (~80 antibodies per EV) was sufficient to achieve maximal CD137 signaling and further increases in copy number showed no additional effect. Subsequently, we investigated if EV conjugation could similarly enhance the immune-stimulatory effect of other classes of immunotherapeutics, such as ligands and cytokines. Conjugation of CD137-ligand (CD137L) on EVs displayed comparable enhancements in stimulatory activity as compared with equivalent quantities of free CD137L, evidenced by the significant increase in IFN-γ release (Figure 2G). Similar enhancements in stimulatory potential were also observed with agonistic OX40 antibody as demonstrated by the increase in T cell proliferation observed upon treatment with EV-OX40 Ab as compared with free OX40 Ab (Figure 2H). However, we did not observe significant enhancement of IL-2 signaling upon IL-2 conjugation to EVs across the range of concentrations tested (Figure 2I). Taken together, these data indicate that EV surface display-mediated enhancement of stimulatory potential was applicable to agonistic antibodies and ligands that bind to crosslinking-dependent TNFRSF members.

However, effective activation of immune cells often requires simultaneous stimulation of multiple receptors. Thus, to determine the optimal orientation for delivering multiple complementary ligands to a single cell, we utilized three agonistic antibodies that work synergistically to achieve T cell activation: CD3, CD137, and CD28. We performed a series of assays comparing the efficacy of EV-*Cis* display (all three ligands conjugated on a single EV) to EV-*Trans* display (each of the three ligands conjugated on separate EVs) or an equivalent dose of free ligands (Figure S6A). A positive control of Dynabeads conjugated with CD3, CD137, and CD28 agonistic antibodies in *cis* was included (Beads-*Cis*). Assessment of T cell activation markers CD69 and CD25 revealed that EV-*Cis* treatments resulted in greater T cell activation compared with both EV-*Trans* and free ligand treatments (Figures 2J and 2K). T cell proliferation monitored with alamarBlue assay reflected a similar trend, with *cis*-EVs resulting in significantly higher levels of T cell proliferation than all other treatment conditions on day 6 (Figure 2L). EV-*Trans* and Free Ligand treatments induced moderate levels of T cell activation and T cell proliferation, with the former demonstrating higher stimulatory effects than the latter. This trend was reflected in the CellTrace dilution assay where up to six generations of T cell division were discerned in EV-*Cis* and Beads-*Cis* treatments as early as day 3, while EV-*Trans* and free ligand treatments did not show clear generational dilution of CellTrace dye at day 3 (Figure S6B). EV-*Cis* treatments also resulted in significantly higher levels of IFN-γ release in supernatants and Granzyme B expression in cytotoxic T cells (Figures S6C and S6D). Taken together, these data suggests that *cis*-display on EVs is the optimal approach to deliver multiple complementary ligands for maximal stimulatory effect.

EV-bound ligands induce more potent anti-tumor responses than free ligands

To evaluate the efficacy of EVs in delivering immunomodulatory ligands for anti-tumor effect, we performed an *ex vivo* tumor cell killing assay utilizing the B16-F10-Luc2 mouse melanoma model—a highly metastatic, immunologically cold tumor model (Figure S7A). Immunomodulatory ligands that have shown to be effective against B16-F10-Luc2 include agonistic CD3 and CD137 antibodies, recombinant mouse IL-2, as well as antagonistic PD-1 and CTLA-4 antibodies.[16–18] The elevated expression of PD-L1 was validated and suggested that together with T cell activation, PD-L1 blockade could potently induce tumor cell killing (Figure S7B).

The candidate immunomodulatory ligands alone, or in different combinations were used to treat a co-culture of B16-F10-Luc2 cells and T cells at a 1:50 ratio to evaluate their relative tumor cell killing efficacy. The tumor cell killing assay revealed that each of the individual

Figure 2. EV-mediated surface display of ligands enhances signaling compared with equivalent doses of free ligands

(A) Schematic depicting the differences in receptor engagement between free agonistic CD137 antibodies, or a similar number of antibodies conjugated on a single EV. (B) Immunofluorescent imaging of T cells incubated with EVs conjugated with an isotype control antibody (EV-IgG) or an agonistic CD137 antibody (EV-CD137 Ab). T cells were stained for CD45 (green) to visualize the cell membrane while EVs were visualized using CellTrace Yellow labeling (red). Cells were counterstained with Hoechst (cyan). Scale bar, 50 μm. (C) EV-cell association assay comparing the relative binding affinity of CD137 antibody-conjugated EVs (EV-CD137 Ab) with EVs conjugated with an isotype control antibody (EV-IgG). EV binding was quantified using flow cytometry staining for GPA, an RBCEV-specific surface marker. (D) Mean fluorescent intensity (MFI) of GPA staining from (C). (E) IFN-γ ELISA for supernatants of T cells treated with equivalent quantities of EV-conjugated, liposome-conjugated or free CD137 agonistic antibody. (F) Effect of increasing doses of CD137 agonistic antibody-conjugated EVs on IFN-γ release. The numbers in μM depict the concentrations of TCO-labeled CD137 antibody incubated with MTet-EVs for the iEDDA reaction. (G) IFN-γ released from activated T cells following stimulation with free (soluble) mouse CD137L or EV-conjugated CD137L. (H) Relative fold change in T cell proliferation in the presence of free OX40 Ab or an equivalent dose of EV-conjugated OX40 Ab quantified using alamarBlue assay. T cells were activated with CD3/CD28 beads prior to OX40 stimulation. (I) Relative fold change in T cell proliferation compared with an untreated control at varying doses of free recombinant mouse IL-2 or EV-conjugated IL-2. (J and K) MFI of CD69 (J) and CD25 (K) on T cells over a period of 5 days following treatment with a cocktail of agonistic CD3/CD28/CD137 antibodies in their free form or conjugated on EVs in *cis*/*trans* orientation. A positive control (Beads-*Cis*) of Dynabeads conjugated with the same ligands in a similar orientation as shown in EV-*Cis* is included. (L) Proliferation of T cells following stimulation with each of the indicated treatments, using alamarBlue assay. For (J)–(L), the asterisks are shown to denote significant differences between EV-*Cis* and EV-*Trans*. For (F)–(L), data were obtained from three to four independent repeats performed using EVs from individual donors and T cells from different mice. MTet: methyltetrazine; GPA: glycophorin A; iEDDA: inverse electron demand Diels-Alder; DOPE: dioleoylphosphatidylethanolamine; PS: phosphatidylserine. Student's two-tailed t test (D) and (E), (G)–(I), two-way ANOVA test (F), (J)–(L): ns, not significant, *$p < 0.05$, ***$p < 0.001$. Error bars represent standard deviation.

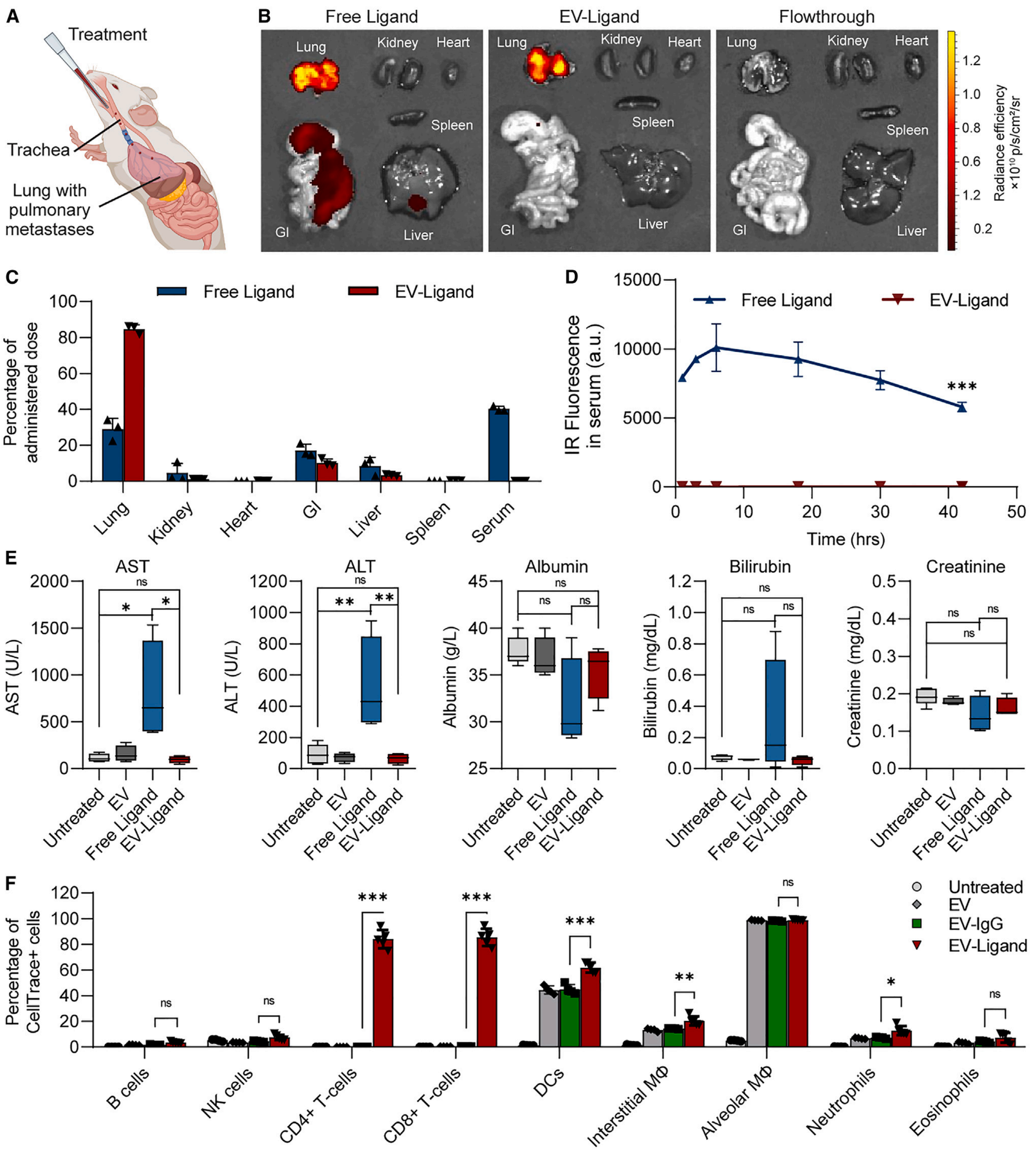

Figure 3. Locally administered EVs are well retained in the immediate tumor microenvironment

(A) Illustration of intratracheal administration in mouse models. (B) Representative near-IR fluorescent images of Free Ligand or EV-Ligand labels in organs acquired using IVIS 4 h post-administration of a single intratracheal dose into C57BL/6 mice. EV-Ligand or free ligand proteins were labeled with CF Dye TFP Ester CF750, a near-IR amine-reactive dye prior to administration to facilitate tracking of *in vivo* biodistribution. A flowthrough control is included for reference. (C) Summary of the *in vivo* biodistribution of Free Ligand and EV-Ligand treatments, represented as the relative percentage of the total administered dose present in each organ ($n = 3$ mice per condition). (D) Relative accumulation of Free Ligand and EV-Ligand treatments in the serum of the mice over a period of 42 h, monitored via measurement of near-IR signals in the serum collected at

(legend continued on next page)

ligands, except for the CTLA-4 antibody, demonstrated appreciable increases in tumor cell killing (Figure S7C). The combination of αCD3, αCD137, αPD-1, and IL-2 demonstrated the most potent effect, achieving over 90% tumor cell death following 6 days of co-culture. Exclusion of any component from the cocktail of immunomodulatory ligands resulted in decreases in tumor cell killing efficiency, while the inclusion of CTLA-4 had no additional effects, suggesting that the combination of αCD3, αCD137, αPD-1, and IL-2 worked in complementarity with non-overlapping functions. Remarkably, despite αPD-1 being an antagonistic antibody that does not depend on signaling to function, conjugating it on EVs in *cis* with the other ligands resulted in significantly improved tumor cell killing than having it in its free form. (Figure S7D). Based on these data, we decided to utilize a combination of αCD3, αCD137, αPD-1, and IL-2 for the *ex vivo* tumor cell killing assay, comparing EV-*Cis* display with equivalent doses of free ligand.

We observed that the EV-Ligand treatment induced significantly higher levels of tumor cell death as compared with an equivalent quantity of free ligand at all ratios tested with both splenocyte and CD8+ T cell co-cultures (Figures S7E and S7F). Remarkably, a 1:1 ratio of tumor cell to CD8+ T cell was able to induce significant cell death following treatment with EV-Ligand, indicating activation of potent anti-tumor cytotoxicity. Control treatments of EV or EV-IgG showed no significant difference compared with untreated co-cultures, indicating minimal effects on tumor cell viability. Of note, the comparable cytotoxicity obtained in the co-culture of CD8+ T cells suggests that much of the cytotoxic effects observed in the splenocyte co-culture were mediated via the actions of stimulated CD8+ cytotoxic T cells. We also observed significant upregulation of Granzyme B (a key marker of T cell cytotoxicity) in CD8 T cells and Ki-67 (a marker of proliferation), CD25, and IFN-γ in total T cells following treatment with EV-Ligand (Figures S7G–S7J). Free Ligand treatments induced significantly lower upregulation of these markers while the control treatments were unable to induce detectable levels of expression. Thus, we can confirm that EV-Ligand treatments can induce superior immune activation and anti-tumor effects *ex vivo*.

Local administration of ligand-conjugated EVs limits their exposure to the immediate tumor microenvironment and abrogates systemic toxicity

To assess the *in vivo* efficacy of our ligand functionalized EVs, we used a lung metastatic model of melanoma that forms pulmonary metastases upon intravenous injection. This model mimics the metastasis of primary tumors into the lung, which are responsible for most fatalities in cancers such as melanoma. Treatments were administered directly into the lungs of mice via intratracheal administration (Figure 3A). We and other groups have previously demonstrated that this method of administration can efficiently distribute EV suspensions throughout the lung at high coverage with a single dose.[9,13,19] It also results in a high local concentration of the treatment in the immediate vicinity of the tumor metastases in the lung. Moreover, in the context of EV-based treatments, it has the advantage of overcoming clearance by the reticuloendothelial system, a phenomenon often observed with intravenous administration.

To assess the *in vivo* biodistribution of each treatment, we labeled Free Ligand and EV-Ligand treatments with a fixable near-infrared (IR) dye and administered a single dose intratracheally. Four hours post-administration, the distribution of each treatment was assessed by tracking the near-IR signal using an *in vivo* imaging system (IVIS). We observed that the EV-Ligand treatment was almost completely localized to the lungs, while the free ligand treatment accumulated prominently in the serum and to a lesser extent in the gastrointestinal tract and liver (Figures 3B and 3C). This is presumably due to the larger size of the EVs slowing down their permeation through tissues, thereby limiting their exposure to systemic circulation. Free ligands and antibodies are smaller and can diffuse through the lung tissue much faster and enter the blood stream more readily. Further analysis revealed that the free ligand accumulated in the circulation of mice and remained at high levels for up to 42 h, possibly owing to the Fc domains on the antibodies (Figure 3D). Remarkably, we did not detect the presence of EV-Ligand in the serum at any of the tested time points. These data indicate that ligands conjugated onto EVs would be well retained near the site of administration, reducing exposure to systemic circulation and other organs. Consequently, this decreases the likelihood of side effects by preventing ligands from interacting with tissues elsewhere in the body.

Given the broad biodistribution of the free ligands, we performed a series of toxicity studies to detect possible adverse effects induced by repeated doses of Free Ligand or EV-Ligand treatments. We observed that free ligand-treated mice showed significant elevations in serum transaminase levels including serum aspartate aminotransferase (AST) and alanine aminotransferase (ALT), indicative of liver toxicity. We also observed non-significant perturbations in albumin, bilirubin, and creatinine content in free ligand-treated mice (Figure 3E). None of the EV-Ligand-treated mice showed any changes in these toxicity parameters from baseline levels, indicating that EV-associated ligands display an improved safety profile. This is in line with other EV studies that report improved toxicity profiles due to the altered biodistribution of ligands displayed on the EV surface.[3] Further investigation into the precise cellular uptake of EV-Ligand treatments (conjugated with αCD3, αCD137, αPD-1, and mIL-2) via flow cytometric immunophenotyping of lung cells revealed high levels of EV-Ligand accumulation in CD4+ and CD8+

each time interval. (E) Blood chemistry analysis of mice administered with six doses of free ligand or an equivalent dose of EV-conjugated ligands, assessed 20 days after the first administration. Mice were administered the treatments intratracheally at 3-day intervals. (F) Cellular uptake of control (EV/EV-IgG) or T cell-targeted EV-Ligand treatments by immune cell subsets in the lung assessed 5 h after administration of a single dose of EVs. For (C)–(F), data were obtained from three to four independent repeats performed using EVs from individual donors. The ligand combination used in panels (B)–(F) consisted of αCD3, αCD137, αPD-1, and mIL-2. Student's two-tailed t test (E) and (F), two-way ANOVA test (D): ns, not significant, *$p < 0.05$, ***$p < 0.001$. Error bars represent standard deviation.

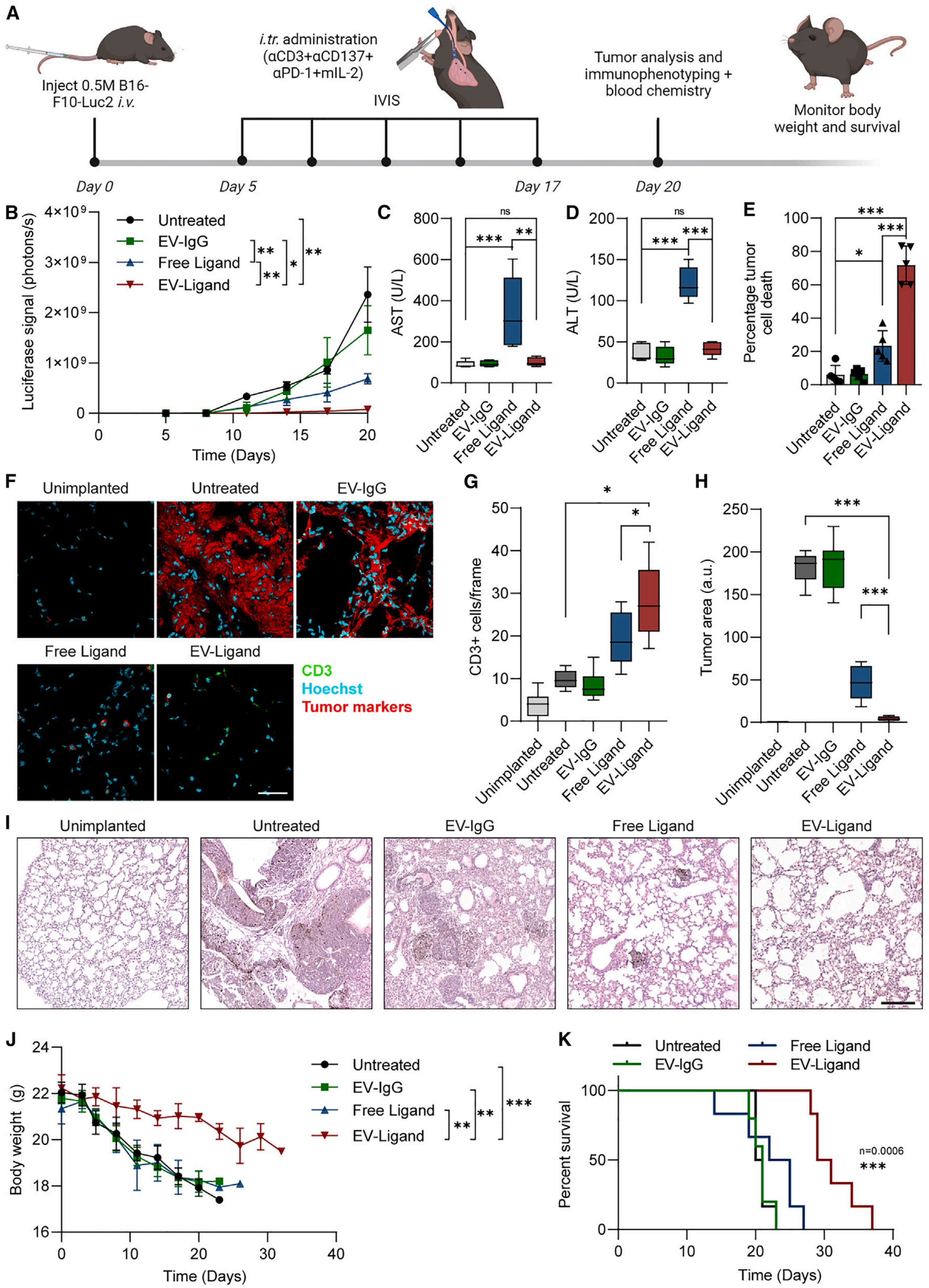

(legend on next page)

T cells (Figure 3F). In contrast, EVs conjugated with a control antibody (EV-IgG) showed little to no accumulation in T cells. These data establish that EV-Ligand treatments can specifically interact with target cells displaying cognate receptors. We also observed that in general, EVs were taken up predominantly by alveolar macrophages, interstitial macrophages, and dendritic cells.

EV-bound ligands display improved tumor suppression and enhanced safety profiles over free ligands *in vivo*

To assess the *in vivo* functionality of EV-Ligand treatments, we developed a lung metastatic B16-F10 melanoma allograft expressing a firefly luciferase reporter by injecting 0.5 M B16-F10-Luc2 cells intravenously. The tumor cells were allowed to form micro-metastases in the lungs of mice before treatments of EV-conjugated immunomodulatory ligands (EV-Ligand) or equivalent quantities of free ligand were administered intratracheally from day 5 (Figures 4A and S8A). Monitoring tumor progression over a period of 20 days via IVIS revealed that EV-Ligand-treated mice showed significantly better tumor suppression compared to free ligand-treated mice (Figures 4B and S8B). Interestingly, the modest tumor suppression effect of the free ligand treatment was accompanied by marked increases in AST and ALT levels (Figures 4C and 4D). In contrast, EV-Ligand treatments did not increase serum AST or ALT levels. On day 20, T cells were isolated from the lungs of treated mice and assessed their tumor-specific cytotoxicity using an *ex vivo* tumor cell killing assay. We observed that T cells isolated from EV-Ligand-treated mice resulted in ~70% tumor cell death, in contrast to only ~20% tumor cell death by T cells from the free ligand-treated mice (Figure 4E), suggesting that EV-Ligand treatments induced higher tumor cell-specific cytotoxicity. Immunofluorescence imaging of lung tumor sections following treatment also revealed significantly higher abundance of CD3-positive cells in the EV-Ligand group compared with free ligand-treated mice or control mice (Figures 4F and 4G). Tumor burden at the endpoint of the experiment was verified by immunofluorescent staining for tumor cell markers and hematoxylin and eosin (H&E) staining, with both readouts corresponding to the IVIS data obtained previously (Figures 4F, 4H, 4I, and S8C).

We repeated the experiment over a longer duration, without the administration of further treatments to determine if the treatments could extend the overall survival of tumor-bearing mice. We observed that EV-Ligand-treated mice showed a significantly slower decline in body weight over the duration of the study. In comparison, untreated, EV-IgG and free ligand-treated mice showed significant decreases in body weight (Figure 4J). In line with this, EV-Ligand-treated mice showed significantly improved survival, although all the mice eventually succumbed to the tumor burden by day 33 post-implantation. Free Ligand-treated mice showed a moderate improvement in overall survival, albeit much lower than EV-Ligand-treated mice (Figure 4K). Together, these data suggest that EV-Ligand treatments can induce enhanced immune activation and tumor suppression, while simultaneously mitigating many of the toxic side effects associated with immunotherapeutics, thereby promoting enhanced overall survival.

To demonstrate that our EV-based delivery approach is also applicable for the treatment of other cancer models where local administration is an option, we established a B16 F10 flank tumor model by injecting 0.5 M cells subcutaneously into mice and treated them with free CD137 agonistic antibody or EV-conjugated CD137 antibody via intratumoral administration. Monitoring tumor progression over 18 days revealed that the EV-Ligand treatment displayed superior suppression of tumor burden as compared to equivalent doses of the free ligand (Figure S8D). We also observed that serum AST and ALT levels were slightly elevated in the mice treated with the free ligand, supporting the hypothesis that not only is the EV-based delivery approach developed here therapeutically more effective and safer, but that it is also applicable to multiple models of cancer (Figures S8E and S8F).

Intraluminally loaded immune agonists benefit from EV-mediated delivery and synergize with surface display of immunomodulatory ligands

We had previously observed that EVs were readily taken up by phagocytic cells upon intratracheal administration, principally macrophages and dendritic cells (Figure 3F). These cells express CD40, a crosslinking-dependent member of the TNFRSF, which plays a major role in modulating anti-tumor immune responses and is a popular target for immunotherapy.[20] Thus, we hypothesized that EVs conjugated with CD40 agonists could be used to achieve effective CD40 signaling in these cells. We prepared primary murine macrophages and dendritic cells from bone marrow isolates and validated CD40 expression in these cells (Figures S9A–S9C). Our data demonstrated

Figure 4. T cell-targeted immunomodulatory EVs improve tumor suppression in a mouse model of lung metastatic melanoma

(A) Outline of the *in vivo* experiment utilized to assess the relative efficacy of EV-Ligand treatments compared with equivalent doses of free ligands. A luciferase reporter was used to monitor the implantation of B16-F10-Luc2 cells and tumor progression in the lung. (B) Luciferase signals from the lungs of mice administered with each of the indicated treatments over a 20-day period, quantified using IVIS. (C and D) AST (C) and ALT (D) levels in the serum of mice on day 20. (E) *Ex vivo* tumor cell killing assay performed using T cells isolated from mouse lungs at the end of the 20-day treatment period. (F) Immunofluorescent staining of lung sections from each treatment condition, assessing T cell infiltration and tumor burden. A cocktail of melanoma-specific markers that are overexpressed in B16 F10-Luc2 cells was used to detect tumor cells (pan-cytokeratin, podoplanin, PD-L1 and firefly luciferase). Scale bar, 50 μm. (G) Quantification of T cells in the lung across each of the conditions in (F). Quantification is based on 20 images from each treatment condition, acquired in a double-blinded manner. (H) Quantification of tumor burden based on 20 tile scans of parasagittal lung sections from each treatment condition. (I) Representative H&E images of lung sections from each treatment condition. Scale bar, 250 μm. (J) Body weight of mice from each treatment group monitored over an extended duration until mice succumbed to the tumor or reached the conditions for symptom-free survival. (K) Survival of mice administered with each treatment over a period of 38 days. (B), (J), and (K) include data from five to seven mice per treatment condition. (C)–(E) include data from five to six mice for each condition. Images in (F) were acquired in a blinded manner. Two-way ANOVA test (B), (J), Log rank (Mantel-Cox) test (K), and Student's two-tailed t test (C)–(E), (G), and (H): $^*p < 0.05$, $^{**}p < 0.01$, $^{***}p < 0.001$. Error bars represent standard deviation.

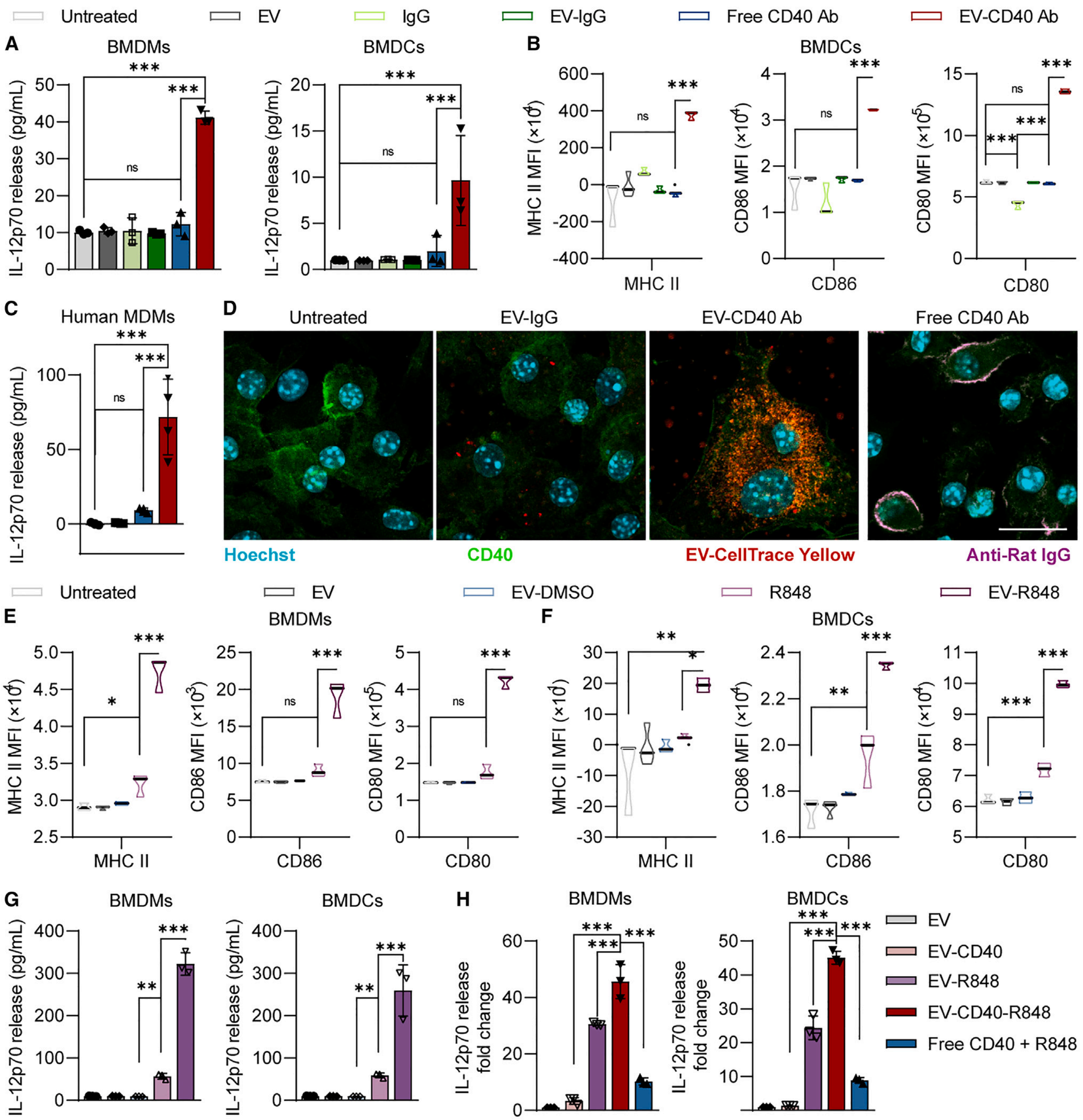

Figure 5. Surface display of ligands synergizes with intraluminal loading of innate immune agonists

(A) IL-12p70 levels in cell culture supernatants determined using ELISA following treatment of bone marrow-derived macrophages (BMDMs) or bone marrow-derived dendritic cells (BMDCs) with free murine CD40 agonistic antibody or equivalent doses of EV-conjugated CD40 antibody. (B) MFI of MHC II and co-stimulatory molecules on the surface of BMDCs following CD40 stimulation as in (A). (C) IL-12p70 release in human monocyte-derived macrophages following stimulation with free agonistic human CD40 antibody or an equivalent dose of EV-conjugated antibodies. (D) Immunofluorescent imaging of BMDMs incubated with control EVs, CD40-conjugated EVs or an equivalent dose of free CD40 antibody. EVs were visualized using CellTrace Yellow labeling (red) and cells were counterstained using Hoechst (cyan). Free CD40 antibody was visualized using a secondary anti-rat IgG (magenta). Cells were stained with a separate CD40 antibody that detected the cytoplasmic domain of CD40 (green). Scale bar, 20 μm. (E and F) MFI of MHC II, CD80, and CD86 on the surface of BMDMs (E) or BMDCs (F) following stimulation with a fixed dose of free R848 or EV-loaded R848. EV-DMSO is included as a vehicle control. (G) IL12p70 release by BMDMs or BMDCs quantified using ELISA after 24 h stimulation with R848 or EV-R848. (H) Fold change in IL-

(legend continued on next page)

that conjugation of an agonistic CD40 antibody on the surface of EVs significantly enhanced its activity compared with equivalent doses of the free antibody as evidenced by the significant increases in IL-12 release and upregulation of MHC II and co-stimulatory molecules on primary murine bone marrow-derived macrophages (BMDMs) and bone marrow-derived dendritic cells (BMDCs), respectively (Figures 5A and 5B).

To confirm that EV-mediated enhancements were translatable to human models, we repeated CD40 stimulation in human monocyte-derived macrophages (MDMs) using a human-specific CD40 agonistic antibody. Our data revealed similar enhancements in IL-12 release by human MDMs upon EV-CD40 Ab treatment. We further investigated the effects of CD40-conjugated EVs on CD40 clustering using confocal imaging. Using a polyclonal CD40 antibody raised against the cytoplasmic domain of CD40, we verified that CD40-conjugated EVs effectively engaged CD40 receptors and induced clustering and internalization (Figure 5D). At high magnification we were able to observe the formation of receptor superclusters in punctate patterns in the cytoplasm upon EV-CD40 Ab treatment (Figure S9D). In contrast, free CD40 antibody showed minimal clustering of CD40 receptors. This suggests that ligand-conjugated EVs can effectively engage cognate receptors and induce clustering and subsequent crosslinking to achieve more efficient signaling.

Our immunofluorescent data had revealed that upon receptor engagement, EVs were eventually endocytosed by the target cells (Figure 5D). Thus, we investigated if we could load innate immune agonists into the lumen of EVs that would activate innate immune pathways in recipient cells upon endocytosis. Studies have shown that CD40 signaling synergizes well with Toll-like receptor (TLR) agonists.[21,22] Thus, we investigated the utility of R848 (Resiquimod), an innate immune agonist of the TLR7/8 pathway that is commonly used in cancer immunotherapy, in enhancing the immune-stimulatory effects of CD40 agonism. Of note, TLR7/8 receptors are present in the endosomal membrane compartments, making EVs ideal vectors to deliver R848. R848 was loaded into EVs via the use of DMSO-mediated permeabilization of the EV membrane, resulting in approximately 10 ng of R848 encapsulated per microgram of EV (Figures S9E and S9F). We demonstrated that the loaded R848 was well retained in the EVs as washing the loaded EVs three, five, or seven times did not significantly decrease stimulation efficiency (Figure S9G). We subsequently compared the efficacy of RBCEV-loaded R848 to an equivalent dose of free R848 by quantifying the increase in expression of CD80, a co-stimulatory molecule expressed on RAW264.7 cells. Our data revealed that the EV-loaded R848 outperformed the free R848 across all tested doses (Figure S9H). More detailed investigation in primary BMDMs and BMDCs revealed that EV-R848 resulted in greater increases in the expression of MHC II, CD86, CD80, and the release of IL-12 as compared with equivalent doses of free R848 (Figures 5E–5G). This is attributed to the high phagocytic capacity of macrophages and dendritic cells for EVs, particularly those containing antibodies targeting cellular receptors, which results in efficient accumulation of loaded R848 in the endosomal compartments of these cells.

Last, we examined the effect of co-treating cells with both R848 and CD40. CD40-conjugated EVs loaded with R848 (EV-CD40-R848) elicited significantly higher IL-12 release from both BMDMs and BMDCs compared with the combined IL-12 release from cells treated with EVs loaded with R848 alone or EVs conjugated with CD40 antibody alone, suggesting a synergistic relationship between R848 and CD40 agonism. Of note, treatment with EV-CD40-R848 was significantly more effective than co-treatment with equivalent doses of free CD40 and R848 (Figure 5H).

EV-mediated delivery of immunomodulatory ligands results in superior anti-tumor immune responses and improved overall survival in an autochthonous cell-derived pancreatic ductal adenocarcinoma model

To further validate the efficacy of our EV-based strategy for the delivery of immunomodulatory ligands, we conducted an *in vivo* experiment using a lung metastatic autochthonous cell-derived pancreatic ductal adenocarcinoma (PDAC) model. KPCY 2838c3 tumor cells were found to express PD-L1 and high levels of a YFP reporter (Figures S10A and S10B). Previous studies using similar models of PDAC have demonstrated effective tumor control via the use of a combination of agonistic CD137 antibodies, PD-1 blockade, agonistic CD40 antibodies, and TLR stimulation. Thus, we developed two distinct EV treatments: (1) EVs displaying αCD137 and αPD-1 antibodies targeted to T cells; and (2) EVs displaying αCD40 antibodies and loaded with R848 targeted to dendritic cells and macrophages. Mice were treated every other day with five doses of EV-based or free ligand formulations 10 days post-implantation. Immune cell composition, tumor burden, and toxicity were assessed on day 30 (Figure 6A).

We hypothesized that EV-mediated delivery of CD40 agonists and R848 would induce IL-12 secretion, which in turn would enhance T cell activation, proliferation, cytotoxicity, and IFN-γ production. This would synergize with T cell-targeted CD137 agonists and PD-1 blockade to augment T cell-mediated anti-tumor responses. Immunophenotyping via multicolor flow cytometry revealed significant changes in immune cell composition following EV-Ligand treatments that were in line with the expected immune activation profile, as illustrated in the t-distributed stochastic neighbor embedding (tSNE) plots in Figure 6B. Most notably, mice treated with the EV-Ligand treatment displayed significant increases in both CD4 and

12p70 expression following treatment with either CD40-conjugated EVs, R848-loaded EVs, or CD40-conjugated R848-loaded EVs. A co-treatment with equivalent doses of free CD40 antibody and R848 is included for comparison. For (A)–(C) and (E)–(H), data were obtained from three to four independent repeats performed using EVs from individual donors and macrophages/dendritic cells from different mice. Student's two-tailed t test: ns, not significant, $^{*}p < 0.05$, $^{***}p < 0.001$. Error bars represent standard deviation.

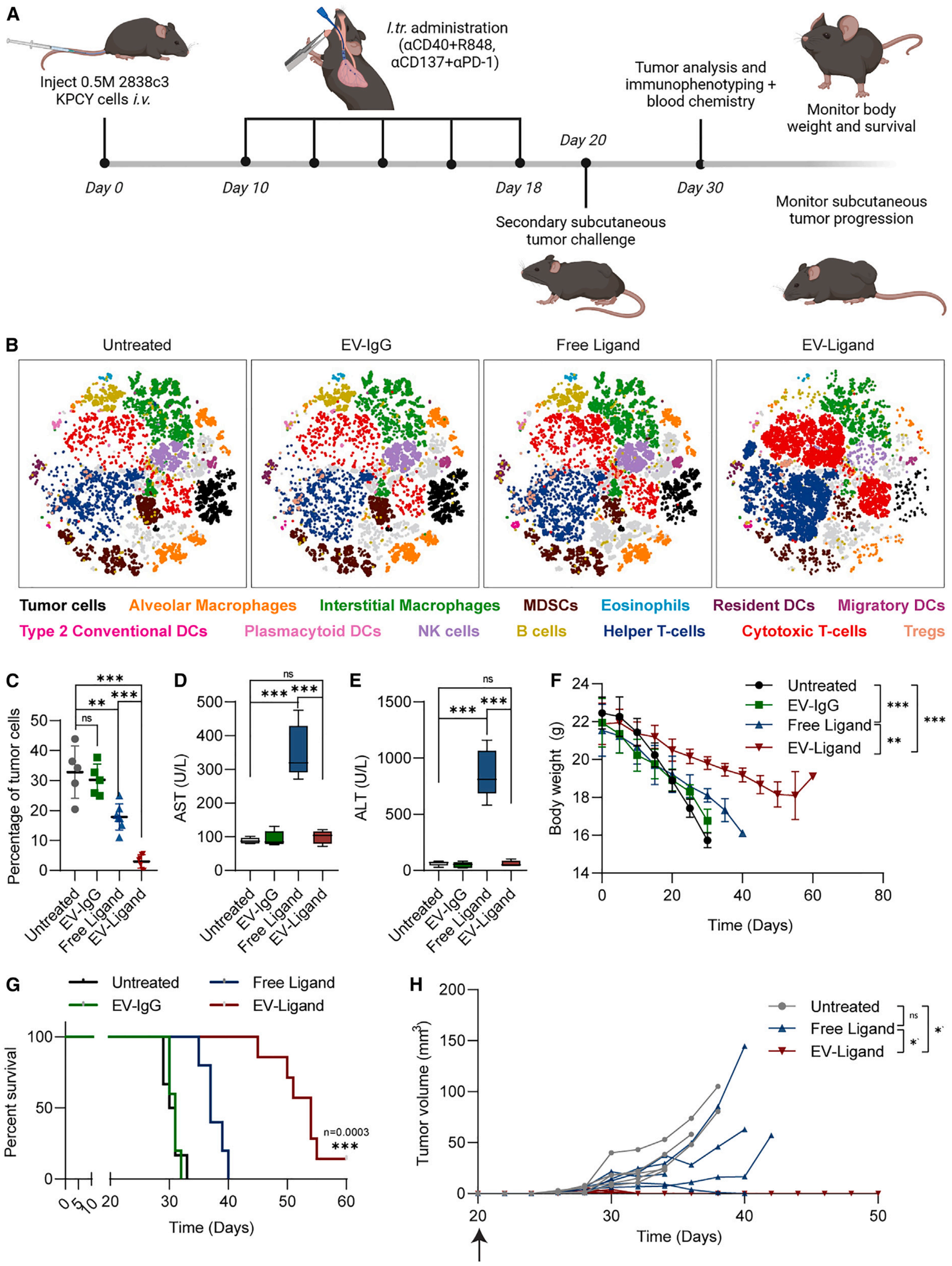

(legend on next page)

CD8 T cells (Figures 6B and S10C). However, we did not observe significant changes in the percentage of dendritic cells (DCs) or interstitial macrophages upon treatment. The increase in T cells was accompanied by significant decreases in immune suppressive immune cell subsets: MDSCs and T regulatory cells (Figure S10D). Interestingly, we also observed a decrease in the relative percentage of B cells, natural killer cells, and alveolar macrophages following EV-Ligand treatment. We presumed that this was attributed more to a substantial increase in the proportion of T cells rather than an actual reduction in these immune cell types. Other changes to the tumor immune milieu upon treatment with EV-Ligand included increased expression of Granzyme in CD8+ T cells, upregulation of MHC II in DC subsets, increased M1 polarization of interstitial macrophages and enhanced expression of *IFN-*γ mRNA, all of which were significantly elevated compared with treatment with equivalent doses of free ligand treatments (Figures S10D and S10E). Interestingly, we were unable to detect significant increases in IFN-α11, IFN- α12, or IL-12, although we did observe a minor increment in IL-12 mRNA levels following EV-Ligand treatment.

Taking into account the significant alterations to the percentage of T cells in the tumor between different treatment groups, we performed further analysis of key T cell markers involved in the generation of anti-cancer immune responses. Detailed analysis of CD69, CD25, PD-1, FoxP3, and Granzyme B expression in T cells revealed that EV-Ligand-treated mice displayed enhanced T cell activation, higher T cell cytotoxicity, and depleted regulatory T cells (Figures S11A and S11B). Taken together, these data suggest that EV-mediated delivery of immunomodulatory ligands can effectively remodel the tumor immune environment.

The changes in immune cell composition observed in the EV-Ligand treatment group were accompanied by a significantly lower tumor burden compared with mice treated with an equivalent dose of the free ligand or control EVs (Figure 6C). While free ligand-treated mice displayed moderate suppression of tumor growth compared with control mice, this was accompanied by an increase in serum AST and ALT levels, indicating significant levels of liver toxicity (Figures 6D and 6E). An extended study also revealed that EV-Ligand-treated mice consistently maintained a higher body weight throughout the study duration compared with all other treatment groups (Figure 6F). This was also reflected in the survival study, where EV-Ligand-treated mice showed a pronounced improvement in overall survival, with one mouse showing complete tumor remission by day 60 (Figure 6G). Meanwhile, free ligand treatments only conferred moderate improvements in survival over control mice. In a separate experiment, we also assessed the development of tumor-specific immune memory by injecting KPCY 2838c3 cells subcutaneously on day 15 and monitoring tumor volume over the next 20 days. Remarkably, none of the EV-Ligand-treated mice developed flank tumors, indicative of the generation of tumor-specific immune activation induced by EV-Ligand treatments (Figure 6H). In contrast, only one of the five free ligand-treated mice showed complete remission upon secondary tumor challenge, while the remaining mice displayed tumors that were generally smaller than the control mice. These data demonstrate that EV-based delivery enhances the efficacy of immunomodulatory ligands to achieve more potent tumor-specific immune responses while concurrently abrogating the onset of systemic toxicity, conferring significant improvements in overall survival.

DISCUSSION

As our understanding of the complex interactions between the immune system and cancer has progressed, so has the discovery of previously unknown targets and ligands that modulate effective anti-cancer immune responses. However, the delivery of these immunomodulatory ligands has remained relatively unchanged, wherein they are typically administered intravenously in their soluble form. Studies have shown that this infusion of immunomodulatory ligands is often suboptimal, resulting in unfavorable biodistributions, the onset of systemic immune overactivation, or off-target toxicity.[1] Recent studies have demonstrated that nanoparticle-based delivery systems could enhance the efficacy of immunotherapies.[23] This is supported by evidence that EVs naturally released by several cell types can effectively modulate the tumor microenvironment.[4,24,25]

The EV-based delivery approach we developed in this manuscript builds upon this knowledge, utilizing our well-validated RBCEV platform together with ultrafast iEDDA-mediated surface functionalization to enhance the performance of clinically approved immunotherapeutic ligands. Our findings demonstrated that EV-based delivery significantly enhanced the signaling efficacy of agonistic ligands, displayed specific biodistribution profiles that enhanced tumor

Figure 6. Multifunctional immunomodulatory EVs result in enhanced tumor suppression in an autochthonous cell-derived model of PDAC
(A) Schematic of the *in vivo* experiment used to assess the anti-tumor efficacy of different treatments. Following intravenous injection of KPCY cells and subsequent intratracheal treatments, mice were euthanized on day 30 to determine tumor burden, measure toxicity parameters, and perform immunophenotyping or alternatively maintained until they reached the criteria for symptom-free survival. In a separate experiment, lung-metastasis-bearing mice were subcutaneously injected with 0.5 M KPCY cells to generate a secondary tumor challenge and the tumor burden was monitored for up to 20 days. (B) tSNE plots based on multicolor flow cytometric analysis illustrating the changes in immune cell composition in tumor-bearing mouse lungs on day 30. An opt-SNE algorithm was used for automated clustering and visualization of immune cell subsets and manually gated immune cell populations were overlaid on the tSNE plots after dimensionality reduction. Unidentified cell populations are left in gray color and the proportion of tumor cells were included in the plot for reference (black). (C) Percentage of tumor cells in mouse lungs in each treatment group at the endpoint (day 30). Tumor cells were gated using flow cytometry based on their expression of YFP. (D and E) AST (D) and ALT (E) levels in the serum of mice on day 30. (F) Body weight of mice monitored over an extended period of time until the mice reached the criteria for symptom-free survival. (G) Survival of mice administered with each treatment over a period of 60 days. (H) Volume of the flank tumor used to assess response to secondary tumor challenge. Mice were injected with tumor cells on day 15 and tumor volume was monitored for 20 days post-implantation as shown in (A). Each line indicates one mouse. (C)–(H) include data from five to seven mice per treatment condition. Two-way ANOVA test (F), mixed effects analysis (H), Log rank (Mantel-Cox) test (G), and Student's two-tailed t test (C–E): *$p < 0.05$, **$p < 0.01$, ***$p < 0.001$. Error bars represent standard deviation.

retention, and abrogated the induction of systemic toxicity, resulting in improved overall survival. Detailed phenotypic analysis of tumors post-treatment revealed significant remodeling of the tumor immune microenvironment toward a pro-inflammatory phenotype suggestive of the generation of strong anti-tumor immunity. Taken together, these data support the use of engineered RBCEVs as potent nanoparticulate vectors for immunomodulatory ligands that offer a range of advantages over conventional delivery approaches such as liposomes and free soluble protein complexes. Indeed, the data presented in this manuscript show that engineered EVs consistently outperformed free ligands and liposomes in terms of efficacy. This along with the enhanced biodistribution profiles and high biocompatibility and safety offered by EVs is indicative that the EV-based approaches developed here have higher potential for superior state of the art.

From a therapeutic perspective, our study establishes engineered EVs as potent and biocompatible nanocarriers that can substantially enhance the therapeutic efficacy and safety of associated immunomodulatory ligands. This enhancement was shown to be mediated through several different mechanisms. First, we demonstrated that conjugation of multiple copies of a single ligand on the EV surface mediated effective multimerization of said ligand, which in turn facilitated efficient endocytosis and receptor crosslinking. Certain receptors, particularly those of the TNFRSF, are heavily dependent on receptor crosslinking for efficient signaling; crosslinked receptors in close proximity can induce significantly stronger signaling, as opposed to stimulation of an equivalent number of spatially separated receptors.[26,27] Our immunofluorescent imaging data support the initial hypothesis that surface display of a sufficient copy number of agonistic antibodies on EVs can induce the formation of higher-order receptor clustering and crosslinking, resulting in enhanced signaling. Indeed, we observed more intense punctate patterns of CD40 receptor clusters upon EV-mediated CD40 stimulation, which has been previously shown in the literature to correlate to higher agonistic activity.[28–30] Notably, all three crosslinking-dependent TNFRSF members used in this study (CD137, OX40, and CD40) displayed pronounced enhancements in signaling upon stimulation with EV-conjugated agonistic antibodies as compared with equivalent doses of the free agonistic antibody. Remarkably, we also observed that EV surface display of immunotherapeutic ligands was superior to liposome surface display, possibly due to the complex composition of EVs such as the presence of functional membrane proteins, a protein corona, a diverse lipidome, and the presence of a glyocalyx, which may positively impact cellular interactions.[31] Indeed, it has been reported in the literature that naive EVs can bind to specific immune cell subsets, often resulting in changes in effector gene expression signatures.[32–34]

We further demonstrated that surface display of multiple complementary immunomodulatory ligands in *cis* configuration on the EV surface results in significantly improved immune activation as compared with *trans* display or stimulation with free ligands. This is presumably due to the concurrent stimulation of the T cell receptor and its co-receptor in close proximity leading to effective T cell activation, which is more effective than disparate stimulation of receptors that are spatially and temporally separated.[26] Indeed, the binding of ligand-conjugated EVs to immune cells could lead to the formation of immune synapses that are reminiscent of the natural cell-cell interactions formed between immune cells, which have been shown to enhance signaling. In addition to enhancing the functionality of ligands displayed on the surface of EVs, we were also able to demonstrate that EVs improve the functionality of ligands loaded inside the EV by enhancing their cellular uptake into specific cell populations. This has been demonstrated before by Peng et al. and Jang et al., who reported that EV-mediated delivery of RIG-I and STING agonists inside EVs enhanced their anti-cancer effects.[9,35] However, in our study, we utilized EVs to deliver R848 into cellular endosomes where the R848 can directly interact with TLR7/8 receptors in the endosomal membrane. This removes the requirement for endosomal escape of ligands required by cytosolic sensors such as RIG-I and STING. Moreover, we were able to demonstrate that encapsulation in EVs not only enhanced the efficacy of the TLR7/8 agonist, but also synergized with surface-displayed CD40 antibodies to achieve significantly enhanced stimulation and cytokine release.

Our data also revealed that conjugation of immunomodulatory ligands on the EV surface significantly altered their biodistribution, resulting in higher levels of retention in the immediate vicinity of the site of administration. This has two advantages from a therapeutic standpoint: (1) It facilitates the retention of a high local concentration of the immunomodulatory ligands at the site of administration, allowing for lower doses to achieve significantly higher therapeutic efficacy; and (2) it prevents the exposure of non-target organs to the immunomodulatory ligands, preventing the onset of systemic toxicity. This was reflected in our *in vivo* treatment experiment, where the EV-Ligand-treated mice displayed significantly better therapeutic outcomes at much lower doses than conventionally used for systemic administration, while simultaneously displaying no detectable levels of liver toxicity. Conversely, treatment with equivalent doses of the free ligand resulted in suboptimal tumor suppression and significant levels of liver toxicity. These data agree with other reports utilizing nanoparticle-based delivery systems to improve the safety and therapeutic effects of immunomodulatory ligands via enhancements in biodistribution.[3,6]

The enhanced tumor-favored distribution of EV-Ligand treatments combined with the enhancements in signaling proffered by EV-mediated crosslinking of receptors, the formation of immune synapses, and the enhanced delivery of intraluminally loaded synergistic immune agonists resulted in our EV treatments consistently outperforming free ligands, displaying effective suppression of two metastatic models of cancer *in vivo*. Remarkably, upon treatment with the EV-Ligand combination, we observed consistent development of tumor-specific immune memory as observed by the resistance of all EV-Ligand-treated mice to secondary tumor challenge, a key factor in preventing tumor recurrence. The implications of the generation of tumor-specific immune memory are significant and offer a glimpse into how the EV-based delivery approach can significantly enhance the generation of tumor-specific immune responses. At the

same time, it is important to recognize that continuous stimulation of immune cells, particularly in the context of cancer, could lead to immune exhaustion. As a result, the combination of ligands used, the dosages, and the frequency of dosing of EV-Ligand treatments would need to be optimized in future studies to ensure the generation of potent, sustainable, and long-lasting anti-tumor immune responses.

In addition to validating the therapeutic efficacy of our approach, we have also investigated several key areas essential for the clinical translation of this delivery platform such as scalability, batch-to-batch variability, safety, and storage. In terms of scalability, we have demonstrated that each element of the EV-based delivery platform presented in this study is clinically scalable. We and others have previously demonstrated the remarkable reproducibility, cost-effectiveness, clinical scalability, safety, and biocompatibility of EVs isolated from human red blood cells.[8,10] We can obtain 2.5×10^{14} RBCEVs on average per unit of human blood, which provides sufficient quantities of EVs for the complete treatment of several human patients in a 5- to 10-dose regimen. The introduction of advanced purification techniques such as tangential flow filtration further enhances yield and automation, making EV purification significantly more convenient and efficient. RBCEVs complement well with iEDDA-based bioconjugation, which also can be linearly scaled up and does not involve the use of large immunogenic proteins or chemical crosslinkers. Furthermore, we have demonstrated that ligand-conjugated EVs show high levels of reproducibility in both their copy number and functionality across different batches, EV donors, and ligands. Last, we have also demonstrated that ligand-conjugated EVs can be safely frozen or lyophilized without loss of functionality and negligible loss of EVs upon recovery. This is a key point in making any therapeutics based on this technology widely accessible as lyophilized EVs can be readily stored at room temperature indefinitely.

Importantly, the EV-based delivery approaches described here are modular and versatile and can be applied to many other agonistic ligands, cytokines, and innate immune agonists for the treatment of cancer or other diseases that require targeting of immune cells, such as inflammatory or autoimmune diseases. At the same time, the therapeutic efficacy of this approach is limited by the availability and nature of the ligands that are available for immunomodulation. Looking to the future, as we better understand the interplay between the immune system and cancer and the nature of immunomodulatory ligands, it is reasonable to expect that there will be more potent molecules capable of enhanced immune modulation, possibly with modifications such as humanization that would simultaneously decrease immunogenicity and enhance biocompatibility. These ligands would still benefit from the delivery approach developed in this study, allowing for significantly improved therapies.

Additionally, this study also makes a significant contribution to the field of EV engineering in the form of the iEDDA-mediated surface functionalization approach we developed. One of the major bottlenecks in the successful application of EV-based therapies has been the lack of effective EV engineering approaches. EV membranes are significantly harder to functionalize than synthetic nanoparticles, with the most popular and efficient approach currently available being parental transfection of immortalized cells. The EV surface functionalization approach we developed is significantly more efficient than any existing approach reported in the literature thus far, pre-isolation or post-isolation, thereby opening new avenues for surface functionalized EV-based applications. Moreover, this modular and versatile approach allows any TCO-labeled molecule to be conjugated efficiently, paving the way for further applications of this approach in EV studies. In essence, our EV surface functionalization approach allows us to imbue EVs with an artificial functional protein corona that confers a variety of therapeutic effects. Notably, it does not involve the use of immunogenic or toxic molecules nor compromise the EVs' endogenous properties, ensuring that the end product is fully biocompatible. This biocompatible profile is maintained by our vector of choice, the RBCEVs, which are biocompatible, non-immunogenic, and can be easily scaled to meet clinical requirements. This is in contrast to other nanoparticulate vectors currently in use in the field, such as synthetic nanoparticles that display poor biocompatibility and are typically immunogenic, or immortalized cell-derived EVs that usually confer risks of tumorigenesis and are harder to translate and scale up to meet requirements for clinical applications. Regardless, we have demonstrated that the EV engineering techniques presented here are translatable to EVs from other sources with comparable efficiency and functionality.

There are, however, certain drawbacks introduced by this EV-based delivery approach that could adversely affect the performance of immunomodulatory ligands. We observed that administered EVs, along with their conjugated immunomodulatory ligands, were cleared from tissues within 8 h post-administration, particularly upon systemic administration. This is in comparison with certain free antibody-based therapeutics that boast a circulatory half-life of many days, a consequence of nFcR recycling. This indicates that treatments would need to be administered more frequently than is typically required for free ligand treatments, making it challenging to treat cancers that may have already spread to multiple organs in the body. It is worth noting that we have observed that our treatments can effectively develop anti-tumor responses following a few administrations and the generated immune memory may be sufficient to achieve tumor regression in more physiologically relevant and less aggressive models of cancer, such as the PDAC model we used in our study. Despite this, we recognize that increased circulatory half-life and tumor homing could significantly enhance this delivery approach further and we are working on further advancing our technology to realize this potential by developing methods to (1) slow down the clearance of EVs upon systemic administration and (2) enhance tumor homing capabilities upon systemic administration. Improvements in these areas would allow us to take advantage of this delivery approach to treat a wider range of cancers at multiple stages and locations in the body.

MATERIALS AND METHODS

RBCEV purification and characterization

RBCEVs were purified and isolated from human RBCs as described in our previous publication.[8] This study exclusively utilizes EVs purified from human RBCs, and the words RBCEV and EV are used interchangeably throughout the study. RBCs were obtained from donors with informed consent following all relevant institutional guidelines. Purified EVs were characterized using western blot, nanoparticle analysis, and single EV flow cytometry.

For western blot analysis, equal quantities of total protein from RBCEV lysates and RBC lysates were electrophoresed in 15%–20% polyacrylamide gels and probed with relevant antibodies. NTA was performed using a NanoSight NS3000 system (Malvern Panalytical, UK) or a Zetaview Particle analyzer (Particle Metrix, Germany) following appropriate dilutions. Single EV flow cytometric analysis was performed using a NanoFCM system (NanoFCM, UK). EVs were stained for 30 min with antibodies/fluorescent probes before being diluted 10-fold and washed to remove excess antibody. The resulting EVs were resuspended, diluted 50-fold, and analyzed using the NanoFCM system, recording all events in a fixed time duration.

EV surface functionalization

Purified RBCEVs were surface functionalized with relevant click handles via the use of amine-reactive STP esters. For iEDDA conjugation, the EVs underwent reaction with methyltetrazine-PEG4-STP ester (Click Chemistry Tools, USA). The optimal degree of labeling was obtained at an EV concentration of 20 mg/mL at an STP ester concentration of 2 mM reacted for 1 h in amine-free PBS at pH 8.5 at room temperature. Similarly, target proteins that were to be conjugated onto the EV surface were labeled with a complementary click handle using either STP/NHS/TFP amine-reactive esters or Maleimide crosslinkers depending on the availability of free amine groups or free thiol side chains from cysteine residues. For iEDDA reactions, proteins were reacted with sulfo TCO-maleimide or TCO-PEG4-TFP ester (Click Chemistry Tools) to install TCO on the protein of interest. The degree of labeling was controlled by varying the concentration of the protein or the reactive TCO label, to ensure that over labeling did not compromise the functionality of the protein of interest.

Following installation of click handles, labeled EVs and proteins were repurified to remove unreacted esters and click handles. EVs were washed by centrifugation a total of four times. Proteins were subjected to buffer exchange via centrifugal filter (Merck Millipore, Germany), Zeba spin buffer exchange columns (Thermo Fisher Scientific, USA), or dialysis chambers (Thermo Fisher Scientific). Purified RBCEVs labeled with methyltetrazine click handles were incubated with proteins labeled with the corresponding TCO click handle to facilitate the iEDDA click reaction to form EVs functionalized with the desired protein. For the conjugation of multiple ligands on the EV surface, proteins were incubated at concentrations corresponding to the desired copy number ratio. αCD137, αCD40, αOX40, and αCD28 antibodies were conjugated at 2.5 μM, αPD-1 antibody was conjugated at 50 μM, αCD3e antibody was conjugated at 0.2 μM, CD137L was conjugated at a concentration of 0.5 μM, and IL-2 was conjugated at a concentration of 0.3 μM. The iEDDA reaction was allowed to proceed at room temperature for 20 min to ensure completion of the reaction, before the EVs were washed via centrifugation and/or SEC. For comparison of iEDDA reactions to SPAAC or OaAEP1 ligation, protocols were adopted from previous publications.[13] Biotin or fluorescent probes conjugated to click handles were utilized (Biotin-DBCO, AZDye 488 TCO [Click Chemistry Tools], Biotin-TCO [Biotium]) to characterize, optimize, and quantify conjugation reactions. Copy numbers of conjugated molecules were determined using ELISA as described in the supplemental information.

Western blot analysis

Western blot analysis on EVs was performed as described in the previous sections. In brief, unmodified or surface functionalized EVs were lysed in RIPA buffer and electrophoresed on 10% SDS PAGE gels. The proteins were subsequently transferred to PVDF membranes, blocked with biotin-free 5% non-fat milk (Bio-Rad, USA), and probed with streptavidin-horseradish peroxidase (HRP) (Thermo Fisher Scientific, USA) (for detection of biotin conjugated on EVs) or HRP-conjugated secondary antibodies (Vector Laboratories, USA) (for the detection of conjugated antibodies). In the experiments performed in this section, the labeling of EV proteins with methyltetrazine was non-specific owing to the nature of amine- and thiol-reactive crosslinking resulting in a non-specific banding pattern representing conjugated proteins. Relative conjugation efficiency in each blot was determined by comparing the total signal in each lane with each other, regardless of molecular weight.

Single EV flow cytometry

Flow cytometric analysis of single EVs was performed as per the MISEV2018 guidelines outlined by the International Society of Extracellular Vesicles[36] and MIFlowCyt-EV framework.[37] EVs were stained with appropriate Alexa Fluor-labeled antibodies (Thermo Fisher Scientific) or streptavidin conjugates (Thermo Fisher Scientific) or stained with EV dyes (CFSE, Thermo Fisher Scientific) or Alexa Fluor 488-labeled Annexin V (Thermo Fisher Scientific). The EVs were washed twice in PBS using centrifugation, diluted to a concentration of 1 μg/mL, and analyzed using a NanoFCM system. The laser power of the 488 was fixed at 8 mW while the 647 laser was set at 20 mW with the SS decay at 1%. The sampling pressure was fixed at 1.0 kPa prior to acquisition and events were recorded for a fixed duration for each sample. All relevant controls including buffer only, antibody controls, detergent controls, and isotype antibody-stained EV controls were included to ensure the authenticity of the observed signals.

Liposome preparation

PS liposomes (Clipos Natural Phosphatidylserine [PS] Lipid Liposomes) made from naturally occurring brain PS and cholesterol in a 70:30 ratio were obtained from CD Bioparticles (CDECPS-1605).

The liposomes had an estimated lipid composition of 42% 18:0 lipid, 30% 18:1 lipid, 2% 20:4 lipid, and 11% 22:6 lipid.

DOPE liposomes were prepared in-house. Briefly, DOPE and cholesterol were mixed at a 4:1 w/w ratio and topped up with absolute ethanol to a concentration of 0.5 mg/mL. The lipids were transferred to a round-bottom flask and placed on a rotary evaporator at 50 mBar, 50 RPM, 25°C until all the ethanol had evaporated. The resulting lipid film that had formed along the wall of the flask was rehydrated with 2.5 mL of distilled water. The suspension was transferred to a falcon tube and subjected to probe sonication on ice for 10 s at 50 kHz. Resulting liposomes were concentrated using an ultrafiltration tube with a 100 kDa molecular weight cutoff. Liposomes were washed via size exclusion chromatography or centrifugation at 60,000 × *g* for 30 min as required. For long-term storage, the liposomes were resuspended in 4% Trehalose and frozen at −80°C.

Liposomes were reacted with methyltetrazine-STP esters using the same protocol as used for conjugating EVs, utilizing the abundant primary amines present on the liposome surface. Liposomes were conjugated with antibodies using iEDDA chemistry using the same protocol outlined previously for EVs. Quantification of IgG copy number, characterization of conjugated liposomes, and subsequent *in vitro* assays were also performed in a similar manner.

Flow cytometry

Flow cytometric analysis for the detection of surface antigens on cells was performed as described before. Cells were washed in fluorescence-activated cell sorting (FACS) buffer, counted, and stained with recommended quantities of antibody. Immune cells expressing Fc receptors were pre-incubated with 0.25 μg of TruStain FcX PLUS (anti-mouse CD16/32) antibody (BioLegend, USA) per million cells for 5 min on ice prior to staining with antibodies. CellBlox Blocking Buffer (Thermo Fisher Scientific) was added to the antibody master mix at 5 μL/test when cell suspensions containing monocytes or macrophages were used in conjunction with NovaFluor dyes. Brilliant Stain Buffer (BD Biosciences, USA) was added into the antibody master mix when more than one Brilliant dye was used in one cocktail. Cells were surface stained on ice for 30 min and washed three times before proceeding to analysis or further processing. Antibodies used for staining are listed in Table S1.

For viability staining, cells were stained directly with the viability dyes SYTOX Blue/PI (Thermo Fisher Scientific) or with the fixable dye ViaKrome 808 (Beckman Coulter, USA). SYTOX Blue and PI were added directly to the cells at a 1,000-fold dilution prior to analysis, vortexed, and immediately run on a flow cytometer. For ViaKrome staining, cells were washed in PBS, before staining with the fixable dye for 5 min at room temperature. The viability dye was subsequently quenched with the addition of 1 mL of FACS buffer, before being washed to remove excess viability dye.

For intracellular staining, cells were fixed with Fixation solution prepared from the True-Nuclear Transcription Factor Buffer set (Thermo Fisher Scientific). Cells were fixed for 1 h at room temperature before being washed twice in True-Nuclear 1X Perm Buffer. The cell pellet was resuspended in 100 μL of the True-Nuclear 1X Perm Buffer and stained with a cocktail of antibodies for intracellular staining for 30 min at room temperature in the dark. The cells were subsequently washed three times before being analyzed using a CytoFLEX LX system (Beckman Coulter). Filter configurations used for each fluorophore are displayed in Table S1. Compensation controls for complex multicolor panels with overlapping fluorophores/spill over between channels was performed using VersaComp Antibody Capture Bead Kit (Beckman Coulter) following the manufacturer's instructions. Acquired data were analyzed using CytExpert Acquisition and Analysis Software Version 2.5 (Beckman Coulter), FlowJo v10 (BD Biosciences, USA) or the Cytobank platform (Beckman Coulter). For large multicolor analyses, dimensionality reduction algorithms tSNE-CUDA or opt-SNE were applied with recommended settings and manually gated immune cell subsets overlaid for verification. Further detailed phenotypic analysis of key surface markers are shown in the z axis of tSNE plots for easy visualization, with biological replicates from each condition concatenated into a single plot.

ELISA

ELISA was utilized to quantitatively determine the copy number of biotin and antibodies on the surface of EVs post-functionalization. To quantify the copy number of biotin per EV, a biotin competition ELISA was utilized.[13] To quantify the copy number of antibodies per EV a commercial sandwich ELISA kit for quantifying total rat IgG was utilized (IgG [Total] Rat Uncoated ELISA Kit with Plates, Invitrogen, USA) was utilized, following the manufacturer's instructions. ELISA for cytokines such as IFN-γ was performed using commercial ELISA kits from BioLegend (ELISA MAX Deluxe Set Mouse IFN-γ, BioLegend, USA).

T cell sorting, activation, and expansion

Splenocytes for *ex vivo* experimentation were obtained from the spleens of healthy mice. In brief, spleens were excised from mice immediately after euthanization and subjected to enzymatic digestion using a Miltenyi dissociator and Collagenase IV using the recommended program. The resulting cell suspension was strained to remove debris and undigested tissue. RBCs were lysed using ACK lysis buffer. The resulting cell pellet was washed and cultured in RPMI-1640 supplemented with 10% heat-inactivated FBS, 2 mM L-glutamine, 100 U/mL mIL-2, and 0.05 mM 2-mercaptoethanol. T cells were sorted from the splenocytes using an untouched T cell isolation kit (Thermo Fisher Scientific) following the manufacturer's instructions. T cells were activated using Dynabeads Mouse T-Activator CD3/CD28 (Thermo Fisher Scientific) following the recommended protocol by the manufacturer. In brief, sorted T cells were incubated with activation beads at a 1:1 ratio for 3 days. Media were topped up on day 2 and on day 3 the concentration of IL-2 was increased up to 1,000 U/mL. The beads-cells suspension was resuspended to detach T cells from beads on day 5 and a DynaMag-5 Magnet (Thermo Fisher Scientific) was used to remove the activation beads

from the expanded T cells. Activated T cells were assessed for the expression of CD69, CD25, and CD137 using flow cytometry.

Binding assay for EVs

EV-cell association assays to verify the affinity of functionalized EVs for target cells were performed as described previously. In brief, control EVs or EVs conjugated with antibodies against specific targets were incubated with cells expressing cognate receptors at 4°C for 30 min. The cells were then washed to remove unbound EVs before being stained with a human RBCEV-specific GPA (CD235a) antibody to detect the presence of RBCEVs on the surface of cells via flow cytometric analysis.

T cell proliferation assays

T cell proliferation was assayed using alamarBlue assay (Thermo Fisher Scientific) following the manufacturer's instructions. Briefly, cells were seeded in 96-well plates and treated. Following treatment, 10 μL of alamarBlue reagent was added per well. The plate was incubated for up to 2 h to allow for development. The plate was analyzed using a plate reader, measuring absorbance at 570 nm. Alternatively, CellTrace dye dilution assay was used to visually confirm the presence of multiple generations of T cells. T cells were labeled with CellTrace Yellow (Thermo Fisher Scientific) by incubating washed T cells at a concentration of 10^6 cells/mL with 5 μM of CellTrace Yellow dye for 20 min at 37°C, protected from light in a water bath. The labeling was subsequently quenched by adding an excess of complete medium containing 10% FBS. The labeled T cells were spun down and washed two times to remove any free dye. The resulting cells were seeded and treated as desired. Following treatment, the cells were analyzed using flow cytometric analysis. New T cell generations were visualized by the dilution of the CellTrace Yellow dye, detectable by the appearance of new peaks at lower intensity. The percentage of cells in each generation was determined by gating on each peak preceding the original peak of undivided cells.

Ex vivo T cell/BMDM/BMDC/MDM stimulation

To assess cytokine release and surface markers on immune cells following stimulation with different treatments, cells were pre-seeded 24 h prior and analyzed at appropriate time points post-treatment. For CD137 stimulation, activated T cells were treated for 24 h prior to measuring IFN-γ release using a LEGEND MAX Mouse IFN-γ ELISA Kit (BioLegend, USA). For OX40 stimulation, T cells were stimulated 48 h after T cell activation, and T cell division was assessed 72 h later. For IL-2 stimulation, activated T cells were cultured with 100 IU/mL IL-2 for 4 days for maximal CD25 expression, before being treated. T cell proliferation was tracked using alamarBlue assay and CellTrace dilution assay following the manufacturers' instructions.

BMDMs, BMDCs, and human MDMs were treated for 24 h prior to RT-qPCR analysis and surface marker analysis. IL-12p70 release was quantified using an ELISA MAX Deluxe Set Mouse IL-12 (p70) (BioLegend). The concentration of the ligands used for *in vitro* stimulation were 2 ng/mL IL-2, 0.1 μg/mL of CD137L (BioLegend), 0.5 μg/mL of αCD137 antibody (clone 3H3), 2 μg/mL of αPD-1 antibody (clone RMP1-14), 0.5 μg/mL of αOX40 antibody (clone OX-86), 0.5 μg/mL of αCD40 antibody (clone FGK4.5/FGK45), 0.5 μg/mL of αCD40 antibody (clone G28.5) (Bio X Cell, USA), 0.5 μg/mL of αCD28 antibody (clone 1C6), 0.1 μg/mL of αCD3 antibody (clone 145-2C11) (Thermo Fisher Scientific), and 10 ng/mL R848 (InvivoGen, USA). An InVivoMAb isotype control raised against trinitrophenol was used as a control IgG for all relevant experiments. To compare the relative efficacy of EVs to liposomes, doses of liposomes corresponding to an equivalent quantity of antibody as were present in the Free Ligand and EV-Ligand treatments were used.

Immunofluorescent imaging

Immunostaining of BMDMs was performed on eight-well chamber slides (Thermo Fisher Scientific). Briefly, 100,000 cells were preseeded per well and cells were treated with fluorescently labeled CellTrace Yellow EVs. Following treatment, cells were washed twice in PBS, fixed in 4% PFA for 20 min, and stained with a polyclonal anti-mouse CD40 antibody (Catalog # 500–3704, Thermo Fisher Scientific) that recognizes an epitope of CD40 that does not overlap with the FGK4.5 monoclonal antibody. Free FGK4.5 anti-CD40 antibody was detected using a fluorescently labeled secondary anti-rat IgG antibody. T cells were treated with EVs in suspension, stained with Alexa Fluor 488 anti-mouse CD45 antibody (103122, BioLegend) to visualize cell membranes, and subsequently fixed in suspension for 30 min at 4°C. The cells were washed to remove excess fixative and immobilized on slides using Cytospin (800 RPM, 5 min). Cells were counterstained with Hoechst 33342, mounted, and imaged using an FV3000 confocal system (Olympus, Japan).

RNA extraction and RT-qPCR

RNA extraction was carried out using TRIzol (Thermo Fisher Scientific) following the manufacturer's instructions. Total isolated RNA was converted to cDNA using a high-capacity cDNA reverse-transcription kit (Applied Biosystems, USA) following the manufacturer's protocol. mRNA levels were quantified using SsoAdvanced universal SYBR Green qPCR kit (Bio-Rad), normalized to *Gapdh*. Primer sequences are included in Table S2. All qPCR reactions were performed using a QuantStudio 6 Flex Real-Time PCR System (Life Technologies, USA).

Generation and maintenance of cell lines

B16-F10-Luc2 cells were obtained from ATCC (USA). B16-F10-Luc2 cells were maintained in DMEM supplemented with 10% heat-inactivated FBS, 1% PenStrep, and 10 μg/mL Blasticidin for selection. KPCY 2838c3 cells were obtained from Kerafast (USA) and maintained in DMEM supplemented with 10% heat-inactivated FBS, 1% PenStrep, and Glutamax.

Murine macrophage differentiation, enrichment, and culture

Primary murine BMDMs were obtained by differentiation of mouse bone marrow isolates. Briefly, C57BL/6 mice were euthanized and their femurs excised under sterile conditions. Femurs were exposed on either end, and a syringe fitted with a 25-gauge needle containing

RPMI was used to flush the bone marrow cells from the bone shaft into a falcon tube. The cell suspension was strained through a 0.45-μm cell strainer. RBC lysis was performed using ACK buffer and the resulting cell suspension was washed twice in RPMI. The cells were subsequently cultured in IMDM supplemented with 1% PenStrep, 100 U/mL M-CSF (BioLegend), and 10% heat-inactivated FBS. Cells were cultured for 7 days, with media being replenished on day 3. On day 7, the cells were washed twice using cold PBS to remove non-adherent cells and fresh M-CSF added to promote proliferation. Cells were detached using Macrophage Detachment Solution (PromoCell, Germany) or a cell scraper and seeded for further experiments.

Human macrophage differentiation and culture

Cryopreserved human PBMCs obtained from donors with informed consent via the Health Science Authority of Singapore were thawed at 37°C in a water bath and treated with DNase I (New England Biolabs, USA) at a final concentration of 100 μg/mL for 15 min at room temperature. The resulting cells were washed and recovered overnight in RPMI-1640 containing 10% heat-inactivated FBS. CD14+ cells were sorted using CD14 MicroBeads, human (Miltenyi Biotec) following the manufacturer's instructions. Sorted CD14+ cells were cultured in RPMI-1640 containing 10% heat-inactivated FBS and 20 ng/mL human M-CSF (BioLegend) for 7 days. Non-adherent cells were washed off on day 7 and the remaining differentiated macrophages were detached using Macrophage Detachment Solution (PromoCell) or a cell scraper and seeded for further experiments.

Murine dendritic cell differentiation, sorting, and culture

Mouse bone marrow was obtained as described above; 1×10^7 cells were cultured in RPMI-1640 containing 10% heat-inactivated FBS, 1% PenStrep, and 50 ng/mL GM-CSF for 7 days. Fresh media was gently added on day 3 and media was replenished on day 6. Non-adherent and loosely adherent cells in the culture supernatant were collected by gentle pipetting and pooled together. A Dynabeads Mouse DC (Dendritic Cell) Enrichment kit was used to enrich DCs using negative selection and F4/80 Microbeads were used to remove macrophages. The resulting cells were stained for CD11c and MHC II and analyzed using flow cytometry.

EV loading

Lyophilized R848 was reconstituted in DMSO to a concentration of 30 mg/mL. EVs were incubated with R848 (Resiquimod) at a final concentration of 1 mg/mL in a solution of 10% DMSO for 30 min at 37°C. EVs were subsequently washed using four rounds of centrifugation to remove free R848.

Generation of lung metastatic cancer allografts

All *in vivo* experiments were conducted according to protocols approved by the Institutional Animal Care and Use Committee under the National University of Singapore. Mice were injected intravenously with 0.5 million B16-F10-Luc2 cells or 0.5 million KPCY 2838c3 cells to generate lung metastatic melanoma and PDAC models, respectively. For the generation of flank tumors, mice were injected subcutaneously with 0.5 M cells in a volume of 50 μL. Tumor volume was calculated using the equation Volume = $(\text{length} \times \text{width}^2)/2$.

EV administration and *in vivo* treatment

Mice were grouped randomly into each treatment group and treated every 2 or 3 days as described in the schematics. The total volume of each treatment was made up to 60 μL and administered intratracheally as described previously.[13] Quantity of each immunomodulatory ligand in a single administered dose are as follows: IL-2: 100 ng, αCD137 antibody (clone 3H3): 25 μg, αPD-1 antibody (clone RMP1-14): 100 μg, αCD40 antibody (clone FGK4.5/FGK45): 25 μg, αCD3 antibody (clone 145-2C11): 5 μg, R848: 4 μg.

Tumor burden (using IVIS) and body weight were monitored at 3-day/5-day intervals. At the end of the study on day 20, the lungs of mice were excised for tumor and immune cell analysis. Blood was collected into serum-separator tubes from mice via cardiac puncture and the serum assayed for the levels of liver transaminase levels. For survival studies, mice were monitored following the conclusion of the treatment until they reached the criteria set out for symptom-free survival (a 20% drop in body weight or severe loss of mobility or cachexia).

Biodistribution assessment

To determine the distribution of free ligands and EV-conjugated ligands following administration, treatments were labeled with an amine-reactive near-IR dye, CF Dye TFP Ester CF750 (Biotium). Free Ligands were directly labeled following the manufacturer's instructions. EV-Ligand treatments were labeled post-conjugation by incubating EVs with 0.25 mM of dye. The fluorescently labeled EVs and ligands were repurified to remove excess fluorophore and administered intratracheally. For organ biodistribution, mice were euthanized 4 h post-administration and their organs excised and imaged using an IVIS. To measure the relative abundance of the treatment in serum, 50 μL blood was collected via cheek bleed at the indicated intervals and near-IR fluorescence measured using a plate reader. For the calculation of total fluorescent signal present in blood, we assumed that each mouse had 58.5 mL of blood per kg of body weight. The percentage of administered dose present in the serum was calculated by comparison with the fluorescent signal of the total administered dose. Specific cellular uptake of EVs was assessed similarly by administering CellTrace-labeled EVs. Flow cytometric analysis was performed to determine EV uptake by specific immune cell subsets.

Immunohistochemistry

Cryopreservation and sectioning of the lungs from tumor-bearing mice was performed as described previously.[13] Sections were fixed in 4% PFA for 20 min before being blocked and permeabilized (10% normal donkey serum, 1% Triton X-100). Tissue sections were stained with a mouse FITC anti-mouse CD3ε antibody (100305, BioLegend) and a cocktail of tumor-specific antibodies: recombinant anti-pan-cytokeratin antibody [AE1/AE3 + 5D3] (ab86734, Abcam), PE anti-mouse podoplanin antibody (127407, BioLegend), PE anti-mouse CD274 (B7-H1, PD-L1) antibody (155403, BioLegend), and

Aati-Firefly Luciferase antibody (ab21176, Abcam). Secondary staining was performed using a donkey anti-Mouse IgG (H + L) highly cross-adsorbed secondary antibody, Alexa Fluor 647 (Thermo Fisher Scientific). Tissues were counterstained with Hoechst 33342 and mounted using VECTASHIELD Vibrance Antifade Mounting Medium (Vector Laboratories). Sections were imaged using an FV3000 confocal microscope (Olympus, Japan) in a double-blinded manner. Images were quantified using ImageJ software.

Histology

Lungs from treated mice were fixed overnight in 10% neutral buffered formalin. Tissues were subsequently transferred to 75% ethanol for storage. Tissues were sequentially dehydrated with increasing concentrations of ethanol using a Leica TP1020 tissue processor (Leica, Germany). Samples were cleared in two baths of Histo-Clear (National Diagnostics, USA) for 90 min each at 37°C, followed by three baths of paraffin wax for 1 h each at 62°C. Tissues were then embedded in paraffin wax and sectioned at a thickness of 5 μm using a Leica RM2255 microtome. Sections were collected on Epredia SuperFrost Microscope Slides (Epredia, USA) and dried overnight. Sections were deparaffinized in three paths of Histo-Clear, rehydrated, and stained using an H&E staining kit (Abcam) following the manufacturer's instructions. Images were acquired using a Leica THUNDER Imager Tissue (Leica). Image analysis was performed using ImageJ v1.8.0 software.

Toxicity assessment

AST and ALT levels were measured in mouse serum following administration of treatments on day 20 post-implantation. Briefly, blood was collected from mice via cardiac puncture. The blood was immediately collected in a serum-separator tube. The serum collected from the serum-separator tubes was serially diluted and quantified using an Aspartate Aminotransferase Activity Assay Kit or an Alanine Transaminase Activity Assay Kit (Colorimetric/Fluorometric) (Abcam, USA). Activity of AST and ALT is represented in U/L, calculated based on a standard curve. Complete blood count and detailed blood chemistry analysis was performed by the NUS Comparative Medicine Diagnostic Lab.

Statistical analysis

GraphPad Prism 8 was used to conduct all statistical analyses. One-tailed Student's t test was used to assess significance levels between controls and experimental samples. For analyzing the difference among multiple treatment groups, two-way ANOVA was utilized. Throughout this study, a p value <0.05 was considered significant. Data in the graphs are represented as the mean, with error bars indicating the standard deviation. Each experiment was repeated at least three times using RBCEVs from independent donors and/or cells from different passages. All data pertaining to animal work are presented as the mean from a minimum of five individual mice.

DATA AND CODE AVAILABILITY

All data and methods pertaining to this manuscript have been presented in the main manuscript file and the accompanying supplemental files. All raw data files are available from the corresponding author upon request.

SUPPLEMENTAL INFORMATION

Supplemental information can be found online at https://doi.org/10.1016/j.ymthe.2024.07.013.

ACKNOWLEDGMENTS

The authors are grateful to Prof. Harvey F. Lodish (MIT/Whitehead Institute) for his insight and guidance. Special thanks to Dr. Waqas Muhammad Usman (Carmine Therapeutics) for valuable advice, Trinh Tran, Nguyen Phuong, and Eric Yeo (NUS) for carrying out RBCEV purification; and Xuan Dang, Hong Anh Le, and Brenda Lam (NUS) for breeding mice and handling the IACUC/IRB protocols. The authors also value the assistance provided by Dr. Cao Dai Phung, Teo Mei Hui, and Annie Hsu (NUS). Schematics and figures were generated at BioRender.com. The authors appreciate the financial support from the Singapore Ministry of Health (MOH-001269). M.K.J. is supported by the SINGA PhD fellowship, funded by A*STAR Singapore.

AUTHOR CONTRIBUTIONS

M.K.J.: Conceptualization, methodology, validation, formal analysis, investigation, writing, review, editing, visualization; Y.S.L.: Validation, investigation, review, editing; D.X.T.L.: Validation, investigation, review; C.Y.L.: Investigation, review, editing; C.G.: Investigation, review, editing; B.Z.J.Y.: Investigation, review, editing; F.Y.X.H.: Investigation, review, editing; R.C.P.: Investigation, review, editing; D.V.H.: Investigation, review; C.D.P: Investigation, review, editing; D.G.T.: Project administration, funding acquisition; M.T.N.L.: Conceptualization, supervision, visualization, project administration, funding acquisition, review, editing.

DECLARATION OF INTERESTS

M.T.N.L. is a scientific co-founder and advisor of Carmine Therapeutics, a start-up company that develops EV-based gene therapy.

REFERENCES

1. Conroy, M., and Naidoo, J. (2022). Immune-related adverse events and the balancing act of immunotherapy. Nat. Commun. *13*, 392. https://doi.org/10.1038/s41467-022-27960-2.

2. Morris, E.C., Neelapu, S.S., Giavridis, T., and Sadelain, M. (2022). Cytokine release syndrome and associated neurotoxicity in cancer immunotherapy. Nat. Rev. Immunol. *22*, 85–96. https://doi.org/10.1038/s41577-021-00547-6.

3. Lewis, N.D., Sia, C.L., Kirwin, K., Haupt, S., Mahimkar, G., Zi, T., Xu, K., Dooley, K., Jang, S.C., Choi, B., et al. (2021). Exosome Surface Display of IL12 Results in Tumor-Retained Pharmacology with Superior Potency and Limited Systemic Exposure Compared with Recombinant IL12. Mol. Cancer Ther. *20*, 523–534. https://doi.org/10.1158/1535-7163.MCT-20-0484.

4. Cheng, Q., Dai, Z., Smbatyan, G., Epstein, A.L., Lenz, H.J., and Zhang, Y. (2022). Eliciting anti-cancer immunity by genetically engineered multifunctional exosomes. Mol. Ther. *30*, 3066–3077. https://doi.org/10.1016/j.ymthe.2022.06.013.

5. Fitzgerald, W., Freeman, M.L., Lederman, M.M., Vasilieva, E., Romero, R., and Margolis, L. (2018). A System of Cytokines Encapsulated in ExtraCellular Vesicles. Sci. Rep. *8*, 8973. https://doi.org/10.1038/s41598-018-27190-x.

6. Kwong, B., Gai, S.A., Elkhader, J., Wittrup, K.D., and Irvine, D.J. (2013). Localized immunotherapy via liposome-anchored Anti-CD137 + IL-2 prevents lethal toxicity and elicits local and systemic antitumor immunity. Cancer Res. *73*, 1547–1558. https://doi.org/10.1158/0008-5472.CAN-12-3343.

7. Zhang, Y., Li, N., Suh, H., and Irvine, D.J. (2018). Nanoparticle anchoring targets immune agonists to tumors enabling anti-cancer immunity without systemic toxicity. Nat. Commun. *9*, 6. https://doi.org/10.1038/s41467-017-02251-3.

8. Usman, W.M., Pham, T.C., Kwok, Y.Y., Vu, L.T., Ma, V., Peng, B., Chan, Y.S., Wei, L., Chin, S.M., Azad, A., et al. (2018). Efficient RNA drug delivery using red blood cell extracellular vesicles. Nat. Commun. *9*, 2359. https://doi.org/10.1038/s41467-018-04791-8.

9. Peng, B., Nguyen, T.M., Jayasinghe, M.K., Gao, C., Pham, T.T., Vu, L.T., Yeo, E.Y.M., Yap, G., Wang, L., Goh, B.C., et al. (2022). Robust delivery of RIG-I agonists using extracellular vesicles for anti-cancer immunotherapy. J. Extracell. Vesicles *11*, e12187. https://doi.org/10.1002/jev2.12187.

10. Zhang, G., Huang, X., Xiu, H., Sun, Y., Chen, J., Cheng, G., Song, Z., Peng, Y., Shen, Y., Wang, J., and Cai, Z. (2020). Extracellular vesicles: Natural liver-accumulating drug delivery vehicles for the treatment of liver diseases. J. Extracell. Vesicles *10*, e12030. https://doi.org/10.1002/jev2.12030.

11. Dang, X.T.T., Kavishka, J.M., Zhang, D.X., Pirisinu, M., and Le, M.T.N. (2020). Extracellular Vesicles as an Efficient and Versatile System for Drug Delivery. Cells *9*, 2191. https://doi.org/10.3390/cells9102191.

12. Pham, T.C., Jayasinghe, M.K., Pham, T.T., Yang, Y., Wei, L., Usman, W.M., Chen, H., Pirisinu, M., Kong, J., Kim, S., et al. (2021). Covalent Conjugation of Extracellular Vesicles with Peptides and Nanobodies for Targeted Therapeutic Delivery. J. Extracellular Vesicles *10*, e12057.

13. Jayasinghe, M.K., Pirisinu, M., Yang, Y., Peng, B., Pham, T.T., Lee, C.Y., Tan, M., Vu, L.T., Dang, X.T., Pham, T.C., et al. (2022). Surface-engineered extracellular vesicles for targeted delivery of therapeutic RNAs and peptides for cancer therapy. Theranostics *12*, 3288–3315. https://doi.org/10.7150/thno.68667.

14. Chen, H., Jayasinghe, M.K., Yeo, E.Y.M., Wu, Z., Pirisinu, M., Usman, W.M., Pham, T.T., Lim, K.W., Tran, N.V., Leung, A.Y.H., et al. (2022). CD33-targeting extracellular vesicles deliver antisense oligonucleotides against FLT3-ITD and miR-125b for specific treatment of acute myeloid leukaemia. Cell Prolif. *55*, e13255. https://doi.org/10.1111/cpr.13255.

15. Nguyen, P.H.D., Jayasinghe, M.K., Le, A.H., Peng, B., and Le, M.T.N. (2023). Advances in Drug Delivery Systems Based on Red Blood Cells and Their Membrane-Derived Nanoparticles. ACS Nano *17*, 5187–5210. https://doi.org/10.1021/acsnano.2c11965.

16. Parin, S., Marie-Andrée, F., Meredith, L.F., Peixin, J., Donastas, S.-T., Lorenzo, F., Roohussaba, K., Chantal Alexia, N., Ignacio, W., Chi-Wan, B.C., et al. (2022). Combined IL-2, agonistic CD3 and 4-1BB stimulation preserve clonotype hierarchy in propagated non-small cell lung cancer tumor-infiltrating lymphocytes. J. ImmunoTherapy Cancer *10*, e003082. https://doi.org/10.1136/jitc-2021-003082.

17. Muth, S.T., Saung, M.T., Blair, A.B., Henderson, M.G., Thomas, D.L., and Zheng, L. (2021). CD137 agonist-based combination immunotherapy enhances activated, effector memory T cells and prolongs survival in pancreatic adenocarcinoma. Cancer Lett. *499*, 99–108. https://doi.org/10.1016/j.canlet.2020.11.041.

18. Zhang, J., Jiang, H., and Zhang, H. (2018). In situ administration of cytokine combinations induces tumor regression in mice. EBioMedicine *37*, 38–46. https://doi.org/10.1016/j.ebiom.2018.09.050.

19. Liu, M., Hu, S., Yan, N., Popowski, K.D., and Cheng, K. (2024). Inhalable extracellular vesicle delivery of IL-12 mRNA to treat lung cancer and promote systemic immunity. Nat. Nanotechnol. *19*, 565–575. https://doi.org/10.1038/s41565-023-01580-3.

20. Beatty, G.L., Li, Y., and Long, K.B. (2017). Cancer immunotherapy: activating innate and adaptive immunity through CD40 agonists. Expert Rev. Anticancer Ther. *17*, 175–186. https://doi.org/10.1080/14737140.2017.1270208.

21. Ahonen, C.L., Doxsee, C.L., McGurran, S.M., Riter, T.R., Wade, W.F., Barth, R.J., Vasilakos, J.P., Noelle, R.J., and Kedl, R.M. (2004). Combined TLR and CD40 triggering induces potent CD8+ T cell expansion with variable dependence on type I IFN. J. Exp. Med. *199*, 775–784. https://doi.org/10.1084/jem.20031591.

22. Bullock, T.N.J. (2022). CD40 stimulation as a molecular adjuvant for cancer vaccines and other immunotherapies. Cell. Mol. Immunol. *19*, 14–22. https://doi.org/10.1038/s41423-021-00734-4.

23. Martin, J.D., Cabral, H., Stylianopoulos, T., and Jain, R.K. (2020). Improving cancer immunotherapy using nanomedicines: progress, opportunities and challenges. Nat. Rev. Clin. Oncol. *17*, 251–266. https://doi.org/10.1038/s41571-019-0308-z.

24. Markov, O., Oshchepkova, A., and Mironova, N. (2019). Immunotherapy Based on Dendritic Cell-Targeted/-Derived Extracellular Vesicles-A Novel Strategy for Enhancement of the Anti-tumor Immune Response. Front. Pharmacol. *10*, 1152. https://doi.org/10.3389/fphar.2019.01152.

25. Buzas, E.I. (2023). The roles of extracellular vesicles in the immune system. Nat. Rev. Immunol. *23*, 236–250. https://doi.org/10.1038/s41577-022-00763-8.

26. Otano, I., Azpilikueta, A., Glez-Vaz, J., Alvarez, M., Medina-Echeverz, J., Cortés-Domínguez, I., Ortiz-de-Solorzano, C., Ellmark, P., Fritzell, S., Hernandez-Hoyos, G., et al. (2021). CD137 (4-1BB) costimulation of CD8(+) T cells is more potent when provided in cis than in trans with respect to CD3-TCR stimulation. Nat. Commun. *12*, 7296. https://doi.org/10.1038/s41467-021-27613-w.

27. Mayes, P.A., Hance, K.W., and Hoos, A. (2018). The promise and challenges of immune agonist antibody development in cancer. Nat. Rev. Drug Discov. *17*, 509–527. https://doi.org/10.1038/nrd.2018.75.

28. Zapata, J.M., Perez-Chacon, G., Carr-Baena, P., Martinez-Forero, I., Azpilikueta, A., Otano, I., and Melero, I. (2018). CD137 (4-1BB) Signalosome: Complexity Is a Matter of TRAFs. Front. Immunol. *9*, 2618. https://doi.org/10.3389/fimmu.2018.02618.

29. Martinez-Forero, I., Azpilikueta, A., Bolaños-Mateo, E., Nistal-Villan, E., Palazon, A., Teijeira, A., Perez-Chacon, G., Morales-Kastresana, A., Murillo, O., Jure-Kunkel, M., et al. (2013). T Cell Costimulation with Anti-CD137 Monoclonal Antibodies Is Mediated by K63–Polyubiquitin-Dependent Signals from Endosomes. J. Immunol. *190*, 6694–6706. https://doi.org/10.4049/jimmunol.1203010.

30. Yu, X., James, S., Felce, J.H., Kellermayer, B., Johnston, D.A., Chan, H.T.C., Penfold, C.A., Kim, J., Inzhelevskaya, T., Mockridge, C.I., et al. (2021). TNF receptor agonists induce distinct receptor clusters to mediate differential agonistic activity. Commun. Biol. *4*, 772. https://doi.org/10.1038/s42003-021-02309-5.

31. van der Koog, L., Gandek, T.B., and Nagelkerke, A. (2022). Liposomes and Extracellular Vesicles as Drug Delivery Systems: A Comparison of Composition, Pharmacokinetics, and Functionalization. Adv. Healthc. Mater. *11*, 2100639. https://doi.org/10.1002/adhm.202100639.

32. Rausch, L., Flaskamp, L., Ashokkumar, A., Trefzer, A., Ried, C., Buchholz, V.R., Obst, R., Straub, T., Brocker, T., and Kranich, J. (2023). Phosphatidylserine-positive extracellular vesicles boost effector CD8(+) T cell responses during viral infection. Proc. Natl. Acad. Sci. USA *120*, e2210047120. https://doi.org/10.1073/pnas.2210047120.

33. Weiss, R., Gröger, M., Rauscher, S., Fendl, B., Eichhorn, T., Fischer, M.B., Spittler, A., and Weber, V. (2018). Differential Interaction of Platelet-Derived Extracellular Vesicles with Leukocyte Subsets in Human Whole Blood. Sci. Rep. *8*, 6598. https://doi.org/10.1038/s41598-018-25047-x.

34. Rausch, L., Lutz, K., Schifferer, M., Winheim, E., Gruber, R., Oesterhaus, E.F., Rinke, L., Hellmuth, J.C., Scherer, C., Muenchhoff, M., et al. (2021). Binding of phosphatidylserine-positive microparticles by PBMCs classifies disease severity in COVID-19 patients. J. Extracell. Vesicles *10*, e12173. https://doi.org/10.1002/jev2.12173.

35. Jang, S.C., Economides, K.D., Moniz, R.J., Sia, C.L., Lewis, N., McCoy, C., Zi, T., Zhang, K., Harrison, R.A., Lim, J., et al. (2021). ExoSTING, an extracellular vesicle loaded with STING agonists, promotes tumor immune surveillance. Commun. Biol. *4*, 497. https://doi.org/10.1038/s42003-021-02004-5.

36. Théry, C., Witwer, K.W., Aikawa, E., Alcaraz, M.J., Anderson, J.D., Andriantsitohaina, R., Antoniou, A., Arab, T., Archer, F., Atkin-Smith, G.K., et al. (2018). Minimal information for studies of extracellular vesicles 2018 (MISEV2018): a position statement of the International Society for Extracellular Vesicles and update of the MISEV2014 guidelines. J. Extracell. Vesicles *7*, 1535750.

37. Welsh, J.A., Van Der Pol, E., Arkesteijn, G.J.A., Bremer, M., Brisson, A., Coumans, F., Dignat-George, F., Duggan, E., Ghiran, I., Giebel, B., et al. (2020). MIFlowCyt-EV: a framework for standardized reporting of extracellular vesicle flow cytometry experiments. J. Extracell. Vesicles *9*, 1713526. https://doi.org/10.1080/20013078.2020.1713526.

YMTHE, Volume *32*

Supplemental Information

Extracellular vesicle surface display enhances the therapeutic efficacy and safety profile of cancer immunotherapy

Migara Kavishka Jayasinghe, Yock Sin Lay, Dawn Xiao Tian Liu, Chang Yu Lee, Chang Gao, Brendon Zhijie Yeo, Faith Yuan Xin How, Rebecca Carissa Prajogo, Dong Van Hoang, Hong Anh Le, Thach Tuan Pham, Boya Peng, Cao Dai Phung, Daniel G. Tenen, and Minh T.N. Le

Supplemental Materials and Methods

Ex vivo tumor cell killing assay

Splenocytes or CD8+ TILs were isolated from the lung homogenates of B16-F10-Luc2 tumor bearing mice using CD8 (TIL) MicroBeads, mouse (Miltenyi Biotec, Germany). Splenocytes or sorted CD8-positive T-cells were subsequently co-cultured with B16-F10-Luc2 cells expressing a firefly luciferase reporter and incubated with desired treatments. Tumor cell death was assayed at 3- and 5-days using luciferase assay (Promega, USA) following the manufacturer's instructions.

RBCEV lyophilization

Lyophilization mixtures of RBCEVs suspended in PBS containing 100 mM trehalose were placed inside a CoolCell® container (Corning, USA) and left to freeze in a -80°C freezer for 4 hours. The frozen samples were then transferred to a FreeZone Plus 4.5 Liter Cascade Benchtop Freeze Dry System (Labconco, USA) for freeze-drying at -84°C, 0.120 mbar. To recover the lyophilised RBCEVs, the vial was reconstituted in deionized water, washed once with PBS by centrifugation at 21,000 g, and resuspended in PBS. The particle count and size distribution were measured using a Zetaview NTA Instrument to determine percentage recovery and size distribution (Particle Metrix, Germany).

EV purification

RBCEVs from mice were purified using similar protocols as outlined in Dang et al.[1] 4TO7 tumor cell-derived EVs were purified as described by Jayasinghe and colleagues.[2]

B16 F10 flank tumor model generation and treatment

8-week old female mice were injected subcutaneously with 0.5 M B15 F10 cells. Tumors were detected by day 3 and mice were treated with 20 µg αCD137 antibody (clone 3H3) either in its free form or conjugated on EVs via intratumoral injection. Flank tumor length and width was measured every 3 days using calipers and the formula 0.5 × Length × Width^2 used to calculate tumor volume. At the endpoint of the experiment (day 18), AST and ALT levels were measured as described previously.

Supplemental Tables

Table S1| List of antibodies used for flow cytometry.

Marker/antigen	Conjugate	Clone	CAT#	Laser/Channel	Emission filter
CD86	BUV395	GL1	564199 (BD Bioscience)	UV405	405/30
CD8a	BUV496	53-6.7	750024 (BioLegend)	UV525	525/40
CD25	BUV737	PC61.5	367-0251-82	UV740	740/35
CD3e	BV421	145-2C11	100341 (BioLegend)	V450	450/45
CD11c	BV510	N418	117337 (BioLegend)	V525	525/40
CD170 (Siglec F)	BV605	E50-2440	740388 (BioLegend)	V610	610/20
CD69	BV650	H1.2F3	104541 (BioLegend)	V660	660/10
CD206	BV785	C068C2	141729 (BioLegend)	V780	780/60
CD45	Alexa Fluor 488	30-F11	103122 (BioLegend)	B525	525/40
MHC II	NovaFluor Blue 610-70S	M5/114.15.2	# M024T02B06 (Thermo Fisher Scientific)	B610	610/20
F4/80	BB700	T45-2342	746070 (BD Biosciences)	B690	690/50
Gr-1	PE	RB6-8C5	108408 (BioLegend)	Y585	585/42
CD370	PE	7H11	143504 (BioLegend)	Y585	585/42
CD45	StarBright Yellow 605	YW62.3	MCA1031SBY605 (Bio-Rad)	Y610	610/20
CD11b	PE-Cy5	M1/70	101210 (BioLegend)	Y675	675/30
CD19	NovaFluor Yellow 700	eBio1D3 (1D3)	M004T02Y06 (Thermo Fisher Scientific)	Y710	710/50
CD279 (PD-1)	PE-Cy7	RMP1-30	109110 (BioLegend)	Y780	780/60
Siglec H	PE-Cy7	eBio440c	# 25-0333-82 (eBioscience)	Y780	780/60
CD49b	APC	HMα2	103516 (BioLegend)	R660	660/10

CD161b/c (NK1.1)	APC	PK136	108708 (BioLegend)	R660	660/10
CD103	APC	2E7	121413 (BioLegend)	R660	660/10
CD235ab	APC	HIR2	306608 (BioLegend)	R660	660/10
FoxP3	Alexa Fluor 700	MF-14	126422 (BioLegend)	R712	712/25
CD4	APC-Fire 750	RM4-5	100568 (BioLegend)	R763	763/43
Granzyme B	CF820	QA16A02	372202 (BioLegend/Biotium)	IR840	840/20

Table S2| List of primers used in this study

**Target**	**Primer sequence (5' → 3')**	
Mouse *IL-2*	Forward	GTGCTCCTTGTCAACAGCG
	Reverse	GGGGAGTTTCAGGTTCCTGTA
Mouse *NF-κB*	Forward	GCTGCCAAAGAAGGACACGACA
	Reverse	GGCAGGCTATTGCTCATCACAG
Human *TNF-α*	Forward	CTCTTCTGCCTGCTGCACTTTG
	Reverse	ATGGGCTACAGGCTTGTCACTC
Mouse *GAPDH*	Forward	AGGTCGGTGTGAACGGATTTG
	Reverse	TGTAGACCATGTAGTTGAGGTCA
Human *GAPDH*	Forward	GGAGCGAGATCCCTCCAAAAT
	Reverse	GGCTGTTGTCATACTTCTCATGG
Mouse *IFN-α11*	Forward	GGTCCTGGCACAAATGAGGA
	Reverse	TCCAAGCAGCAGATGAGTCC
Mouse *IFN-α12*	Forward	AAGACTGAGTGAGAAGGAGTGAG
	Reverse	GAGATGCCAGAATTTGAGCAGTG
Mouse *IFN-γ*	Forward	GCCACGGCACAGTCATTGA
	Reverse	TGCTGATGGCCTGATTGTCTT
Mouse *IL-12*	Forward	TGGTTTGCCATCGTTTTGCTG
	Reverse	ACAGGTGAGGTTCACTGTTTCT

Supplemental Data

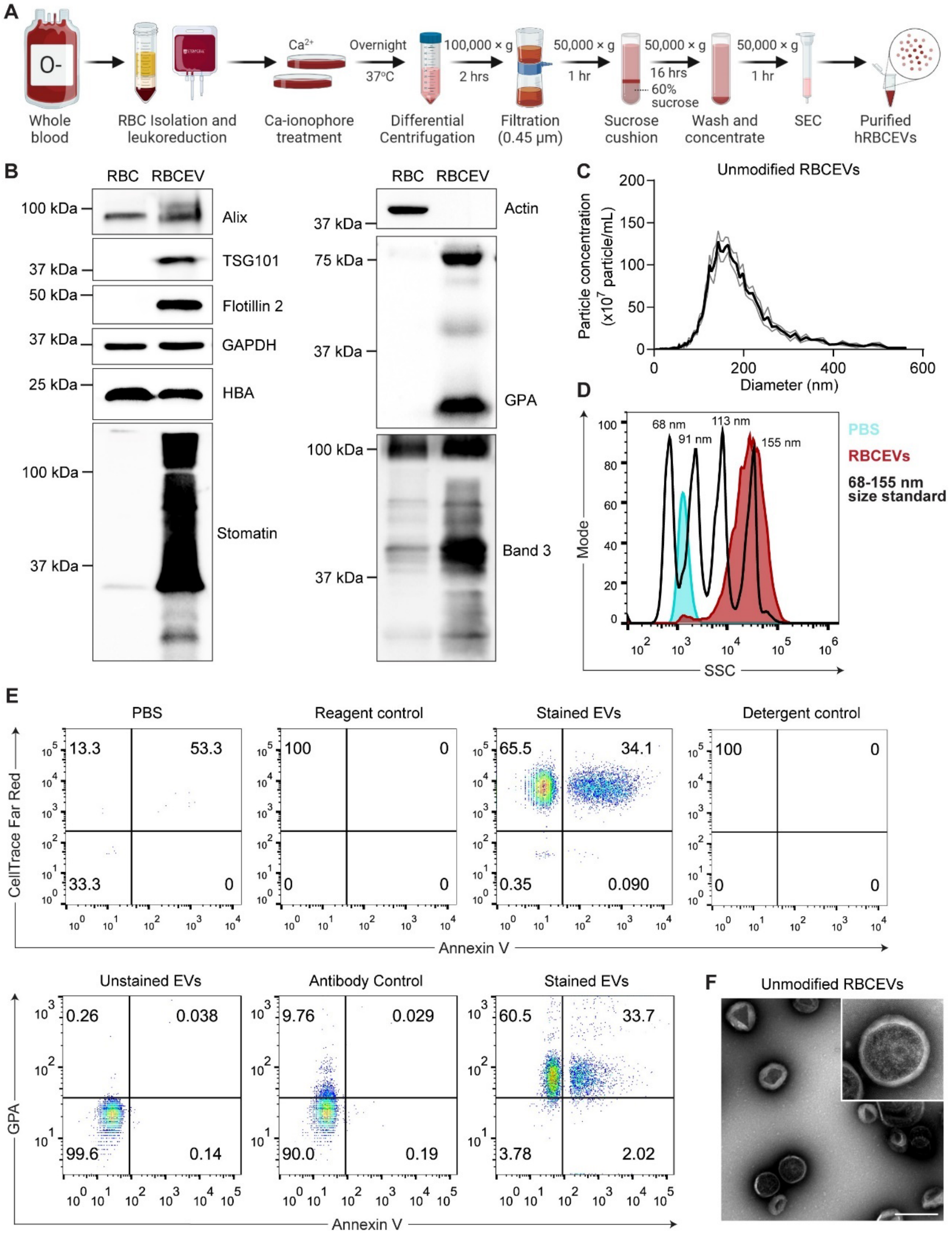

Figure S1| RBCEV purification and characterization. (A) Experimental schema used for the induction, isolation and purification of RBCEVs. (B) Western blot characterization of RBCEVs, comparing relative protein content compared to parental RBCs. (C) Size distribution of unmodified RBCEVs determined using NTA. (D) Gating strategy used to gate out RBCEVs when performing single EV flow cytometric analysis. A buffer control and size standard are included for comparison. Reference diameters for the size standard silica nanospheres are included above each peak. (E) Single EV flow cytometric characterization of RBCEVs, staining for GPA, PS (using Annexin V) and CellTrace Far Red. (F) TEM images of unmodified RBCEVs. Scale bar: 400 nm. NTA: nanoparticle tracking analysis, GPA: Glycophorin A, PS: phosphatidylserine. In (B) molecular weights of protein markers in kDa are shown on the left.

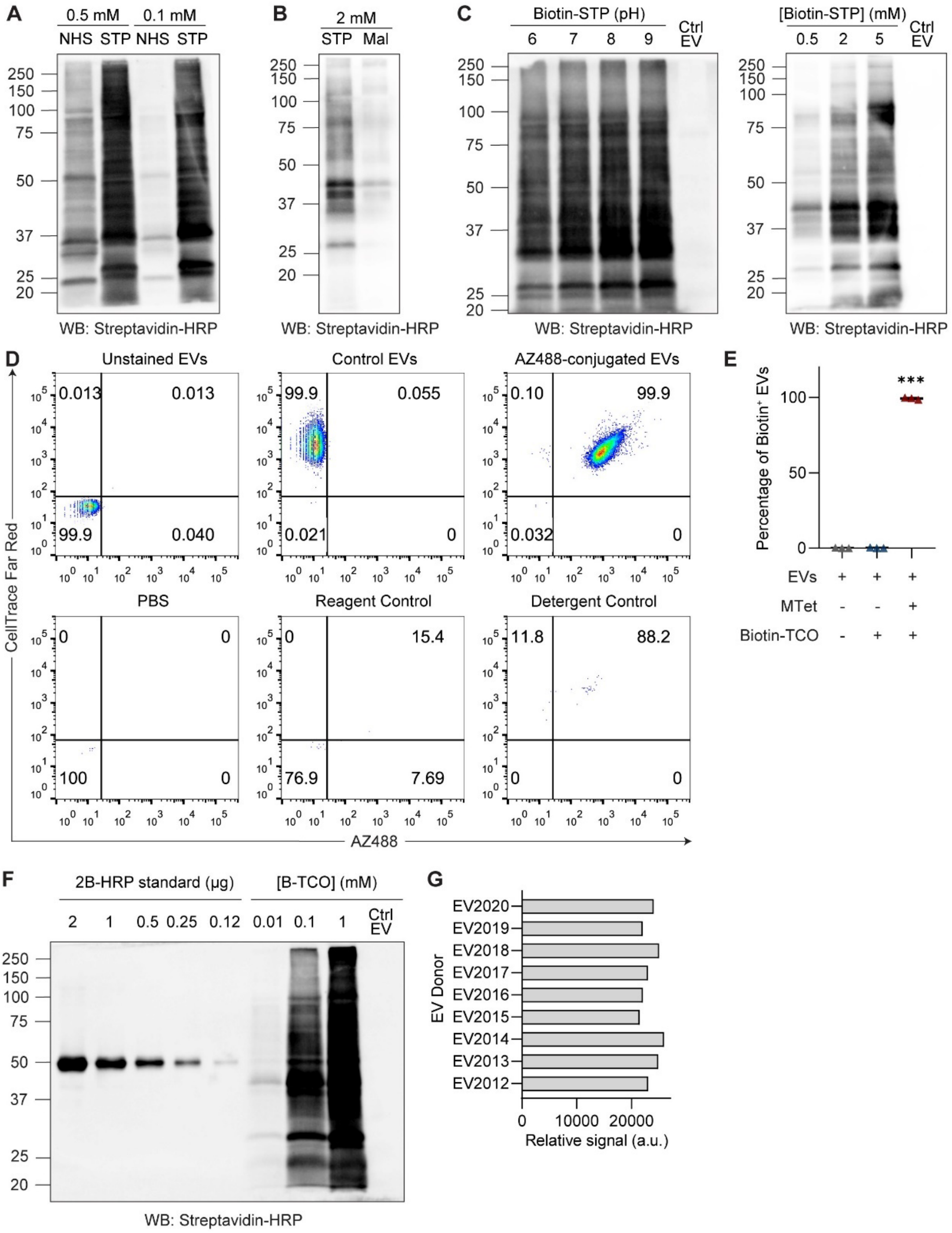

Figure S2| Optimization of ester-activated crosslinking and iEDDA reactions. (A-B) Comparison of relative EV surface modification efficiency of STP ester compared to equivalent concentrations of NHS ester (A) or Maleimide (Mal) (B). Modification efficiency was assessed using reactive esters/maleimide carrying a biotin probe which was detected via western blot. (C) Western blot analysis assessing the relative yield of Biotin-STP reacted with EV surface proteins under different pH or biotin-STP concentration. (D) Flow cytometry dot plots depicting AZ488-TCO conjugation onto MTet-EVs acquired using a NanoFCM system. PBS, reagent controls and detergent controls are included to demonstrate the accuracy of EV detection and all samples were co-stained with CellTrace Far Red to identify EV populations more accurately. (E) Percentage of RBCEVs conjugated with biotin as assessed by single EV flow cytometry. (F) Western blot assessing the conjugation efficiency of increasing concentrations of Biotin-TCO with MTet-EVs. (G) Relative biotin signal of MTet-EVs sourced from separate blood donors that were reacted with Biotin-TCO, quantified using western blot. NHS: N-Hydroxysuccinimide, STP: 4-Sulfo-2,3,5,6-tetrafluorophenyl, TCO: trans-cyclooctene, iEDDA: inverse electron demand Diels–Alder. In (A)-(C) and (F), molecular weights of protein markers in kDa are shown on the left. Error bars represent standard deviation.

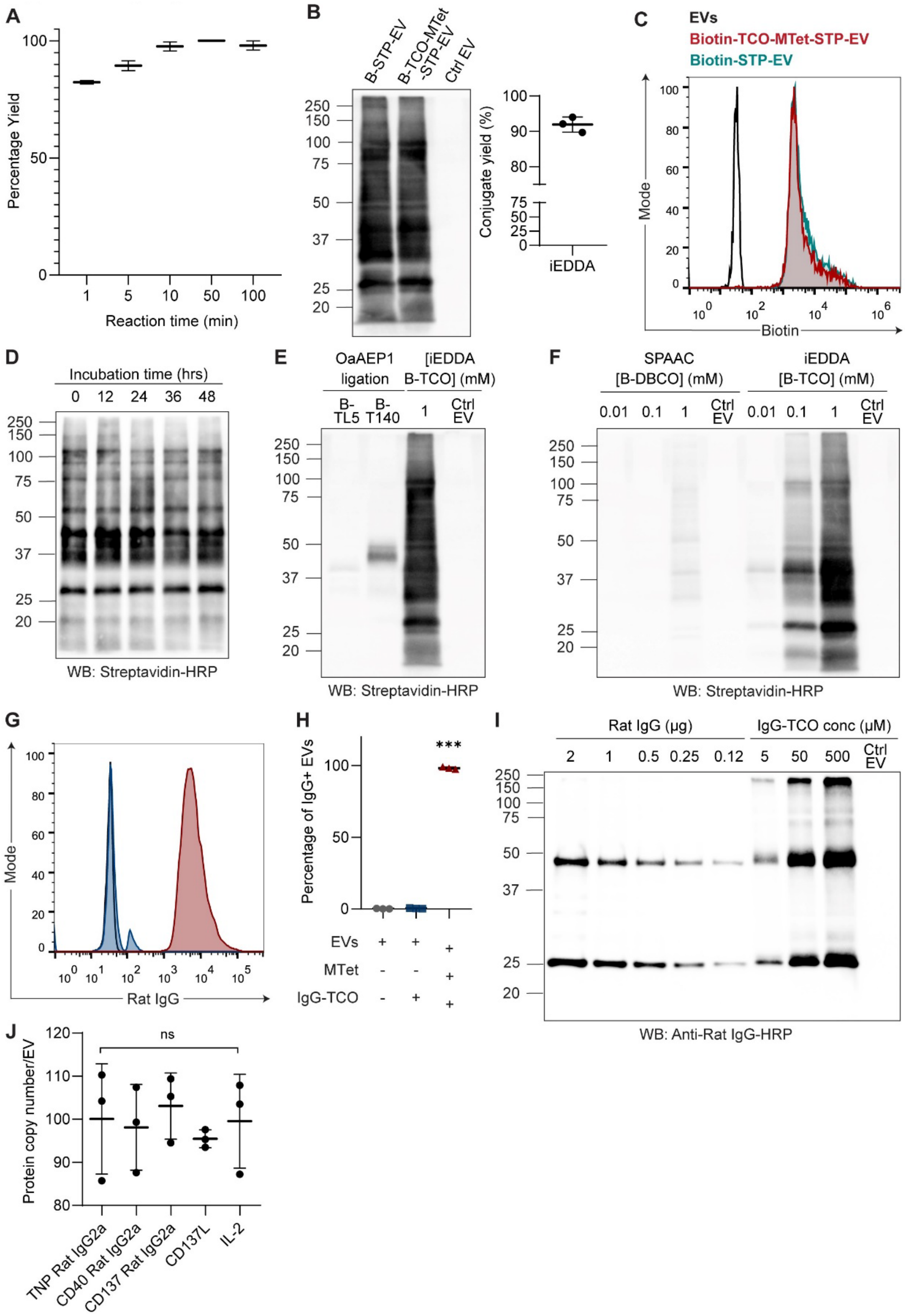

Figure S3| Characterizationon of iEDDA-mediated EV surface functionalization. (A) Percentage yield of the iEDDA reaction over time assessed by the reaction between MTet-EVs with Biotin-TCO. (B) Relative efficiency of iEDDA-mediated conjugation assessed using western blot comparing banding intensity between Biotin-STP-EVs and Biotin-TCO-MTet-EVs. (C) Single EV flow cytometric analysis of Biotin-TCO conjugated EVs as compared to EVs directly reacted with Biotin-STP or control unconjugated EVs. (D) Relative stability of iEDDA-conjugated EVs over a period of 48 hours following incubation in human serum at 37 °C. (E-F) Comparison of iEDDA-mediated conjugation with enzymatic ligation (E) or SPAAC-mediated click chemistry (F). (G) Single EV flow cytometry histogram depicting the conjugation of a TCO-labelled rat IgG onto MTet-labelled EVs. (H) Percentage of EVs conjugated with rat IgG as determined in (G). (I) Western blot analysis assessing the conjugation of IgG onto EVs via iEDDA-

mediated conjugation. Western blot was run under reducing conditions. (J) Copy number of different proteins conjugated on EVs using the same reactions conditions (2.5 µM concentration, 10 minutes). Figures (A), (B), (H) and (J) represent data from 3 individual replicates prepared from separate batches of EVs. MTet: Methyltetrazine, TCO: trans-cyclooctene, iEDDA: inverse electron demand Diels–Alder, SPAAC: strain-promoted azide–alkyne cycloaddition. P value (***$P < 0.001$) was determined using Student’s two-tailed t-test. In (B), (D)-(F) and (I) molecular weights of protein markers in kDa are shown on the left. Error bars represent standard deviation.

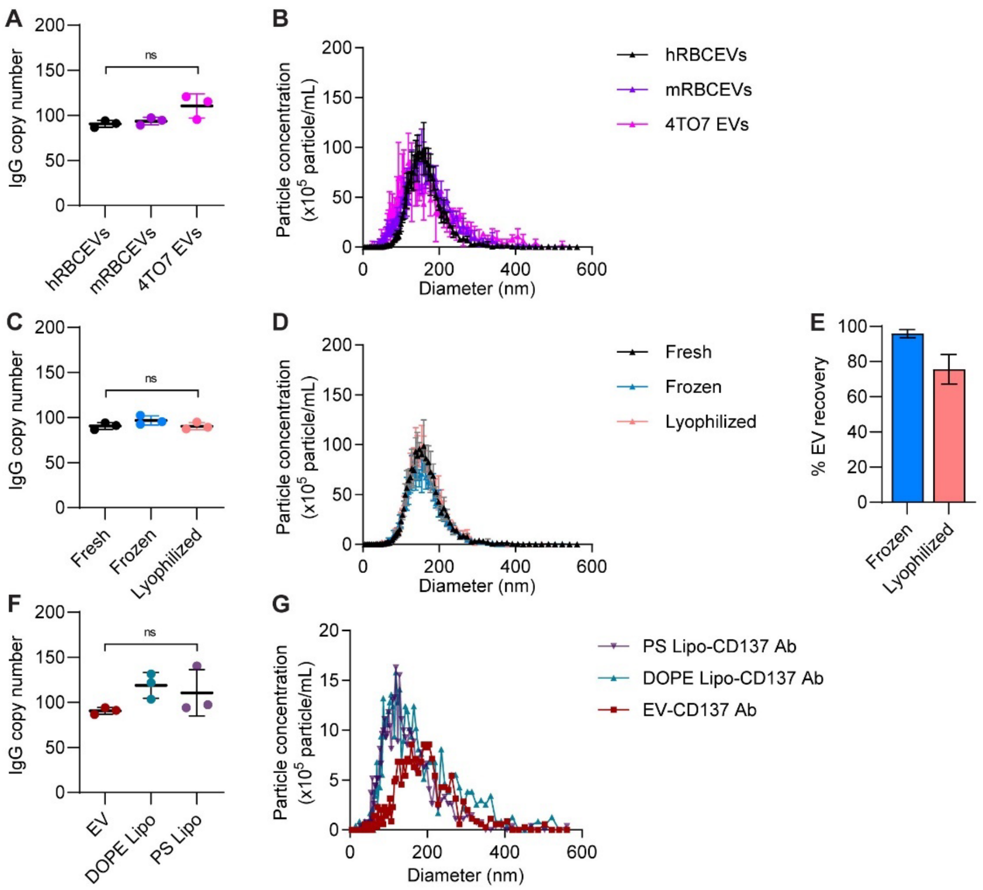

Figure S4| iEDDA-mediated conjugation is stable, versatile and translatable to EVs from other sources and nanoparticles. (A) Copy number of IgGs conjugated on EVs from different sources following the same iEDDA-mediated conjugation protocol (2.5 µM concentration, 10 minutes). (B) Size distribution of each of the EVs from (A) following iEDDA-mediated conjugation. (C) Copy number of IgGs remaining on IgG-conjugated EVs following storage under different conditions. (D) Size distribution of conjugated EVs following storage and recovery for each of the storage conditions in (C). (E) Percentage EV recovery for EVs frozen at -80 °C or lyophilized. (F) Copy number of CD137 antibody present on a single EV or liposome following iEDDA-mediated conjugation at an IgG-TCO concentration of 2.5 µM. (G) NTA analysis displaying the size distribution of DOPE and PS liposomes following conjugation to CD137 antibody using iEDDA chemistry. The size distribution of EVs conjugated with CD137 antibody is overlaid for comparison. Figures (A), (C) and (F) represent data from 3 individual replicates prepared from separate batches of EVs. TCO: trans-cyclooctene, iEDDA: inverse electron demand Diels–Alder, DOPE: dioleoylphosphatidylethanolamine, PS: phosphatidylserine. P value (***P < 0.001) was determined using Student's two-tailed t-test. Error bars represent standard deviation.

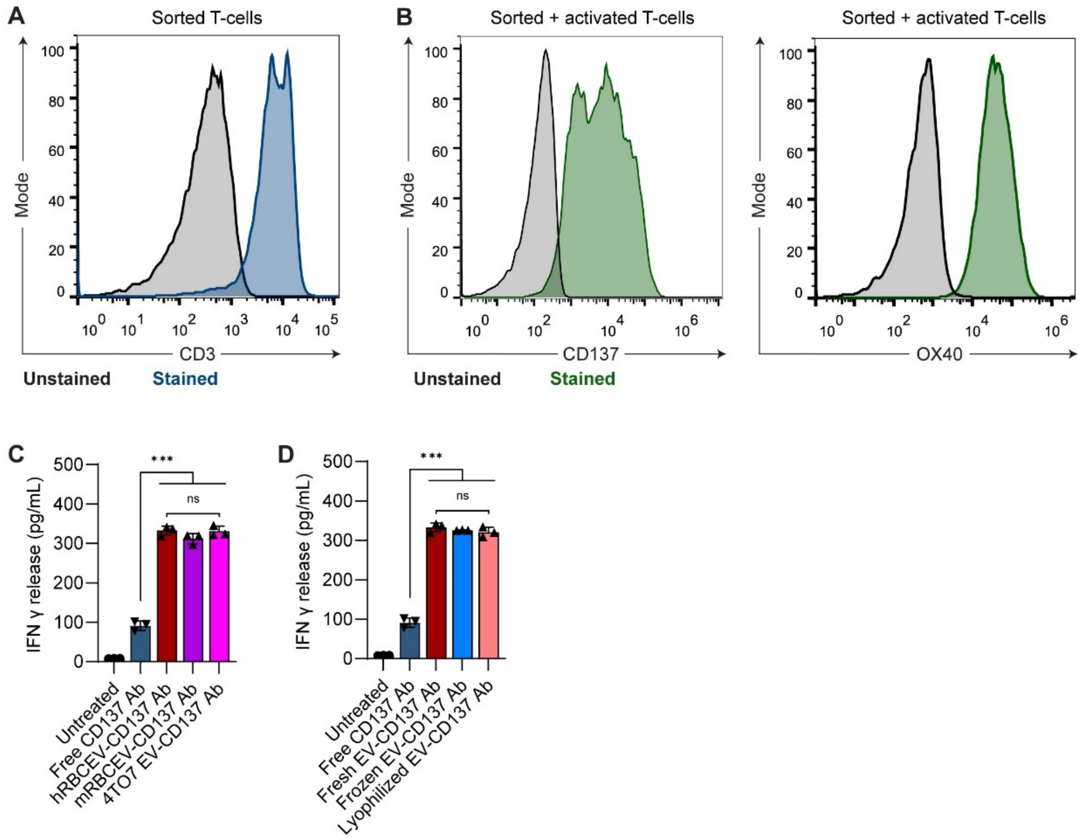

Figure S5| Characterization of *in vitro* functionality of ligand conjugated EVs. (A) Flow cytometric analysis of T-cells after negative selection for T-cells using an untouched mouse T-cells kit. (B) Expression of CD137 and OX40 on T-cells following 3 days of stimulation with mouse T-Activator CD3/CD28 Dynabeads™. (C) Relative stimulation efficiency of EVs from different sources conjugated with an agonistic CD137 antibody assessed via IFN-γ release. (D) IFN-γ release from activated T-cells following treatment with human RBCEVs conjugated with agonistic CD137 antibody that were stored under different conditions. hRBCEV: human red blood cell-derived EVs, mRBCEV: mouse red blood cell-derived EVs. Student's two-tailed t-test: ns – not significant, *P < 0.05, ***P < 0.001. Error bars represent standard deviation.

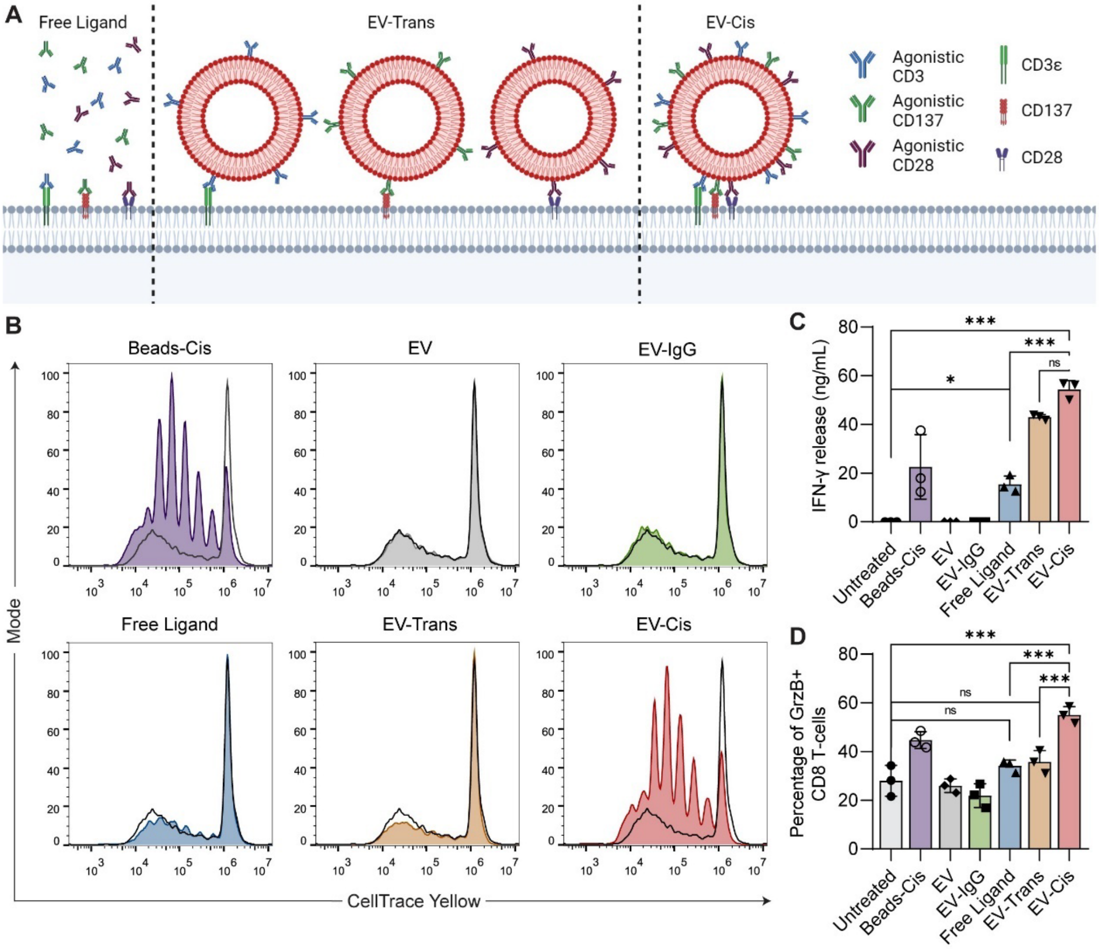

Figure S6| Display of multiple complementary ligands in *cis* is superior to *trans* display. (A) Schematic illustrating the experimental design utilized to compare the relative signalling efficiency of *cis* versus *trans* EVs as compared to free antibodies. Unactivated T-cells were used for this experiment. (B) CellTrace proliferation assay conducted on T-cells on day 3 following stimulation with each treatment. The unstimulated cells are overlaid with each histogram in black for comparison. (C) IFN-γ ELISA of the supernatant of T-cells 6 days post-treatment. (D) Percentage of Granzyme B positive CD8+ T-cells in each treatment condition on day 6 assessed using intracellular flow cytometry. For (C)-(D), each replicate was performed using EVs from individual donors and T-cells from different mice. Student's two-tailed t-test: ns – not significant, *$P < 0.05$, ***$P < 0.001$. Error bars represent standard deviation.

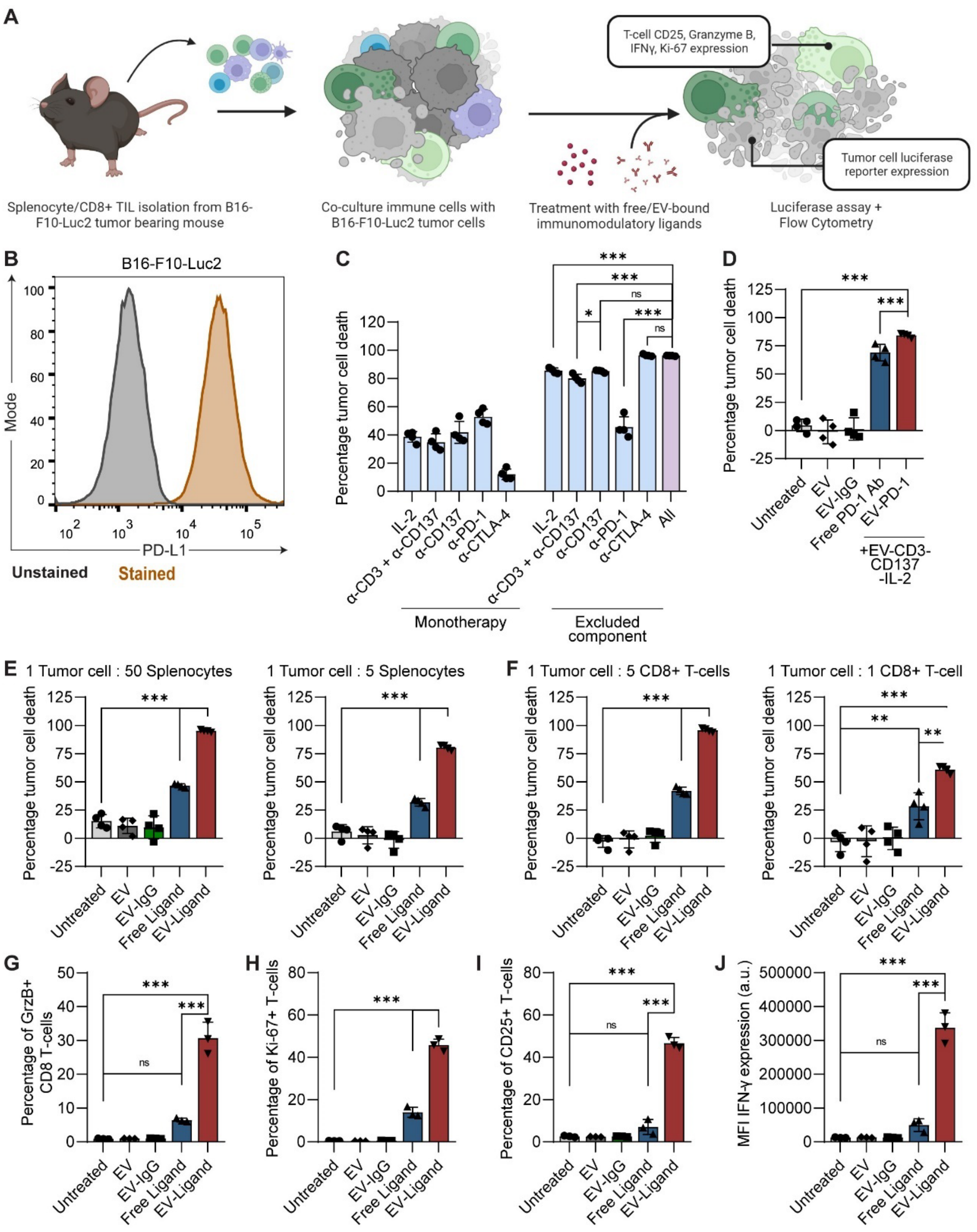

Figure S7| Multifunctional immunomodulatory EVs display superior tumor cell suppression compared to free ligands *in vitro*. (A) Design of the *ex vivo* tumor cell killing assay. Immune cells were isolated from tumor bearing mice, followed by co-cultures with B16-F10-Luc2 cells expressing a firefly luciferase reporter. (B) Expression of PD-L1 in B16-F10-Luc2 cells determined using flow cytometric analysis. (C) Evaluation of tumor cell killing efficacy of 5 candidate immunotherapeutics either alone or excluded from a combined cocktail. Efficacy was assessed via percentage tumor cell death obtained via luciferase assay. (D) *Ex vivo* tumor cell killing assay assessing the relative efficacy of PD-1 in its free form versus upon conjugation to EVs. Both treatments with the PD-1 antibody also included EVs conjugated with αCD3, αCD137 and mIL-2. (E-F) Percentage tumor cell death following co-culture with different ratios of either splenocytes (E) or sorted CD8 T-cells (F) at the stated ratios. (G-J) Expression of granzyme B in CD8-positive T-cells (G), Ki-67 in total T-cells (H), CD25 in total T-cells (I) or IFN-γ in total T-cells (J) following co-culture and treatment. For (C) and (D), each replicate was performed using EVs from individual donors. For (E)-(J), each replicate was performed using EVs from individual donors and T-cells isolated from separate mice. Student's two-tailed t-test: ns – not significant, *P < 0.05, ***P < 0.001. Error bars represent standard deviation.

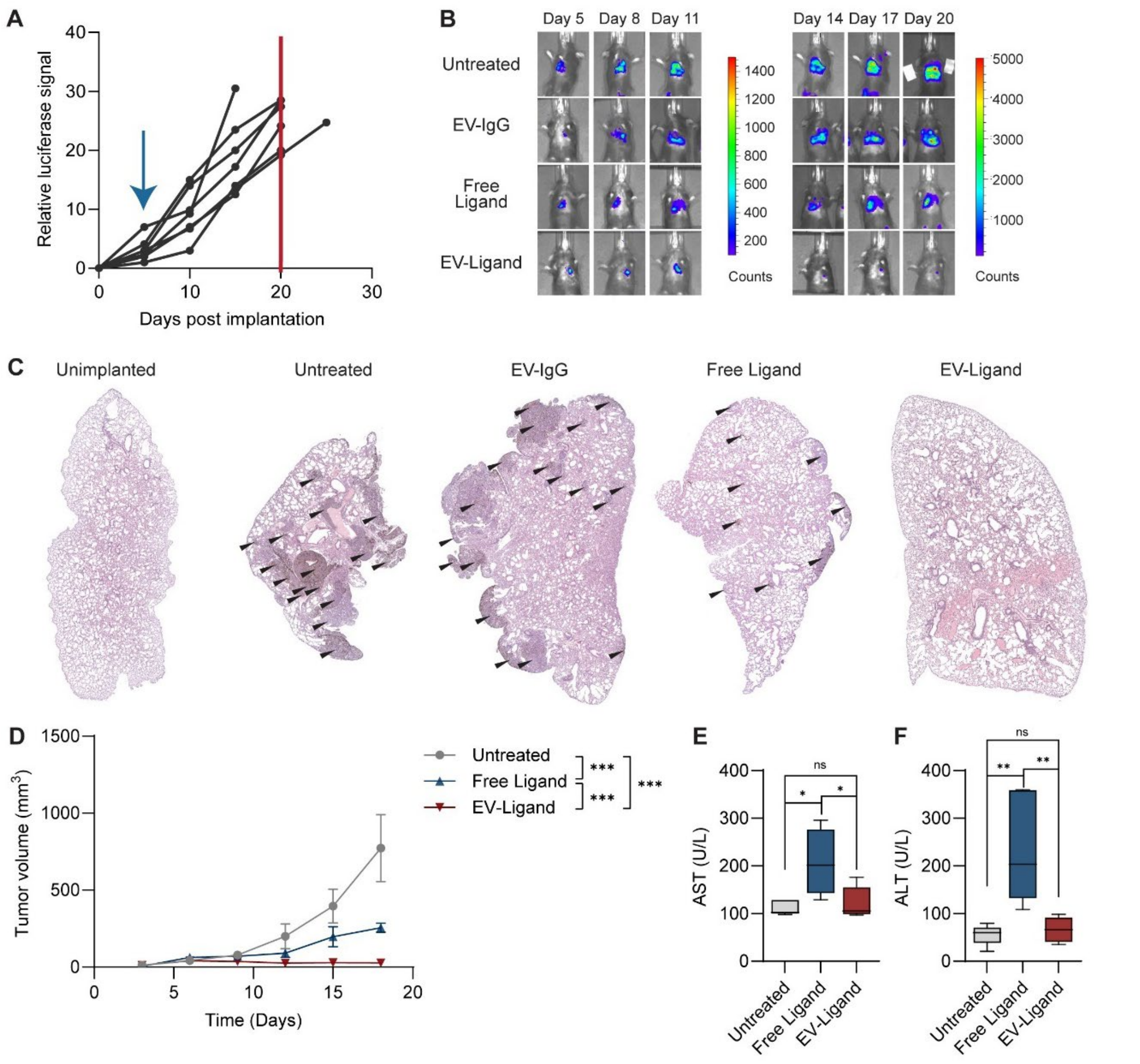

Figure S8| EV-Ligand treatments show superior *in vivo* tumor suppression in multiple models of melanoma. (A) Relative luciferase signals from mice injected intravenously with 0.5M B16-F10-Luc2 cells monitored over a period of 25 days. The blue arrow indicates the timepoint selected for starting treatment administration and the red line indicates the end point of the study, at which most mice succumbed to tumor burden. (B) Representative IVIS images of mice from each treatment group over the 20-day treatment period. (C) Representative H&E images of complete lung sections from tumor bearing mice 20 days post-treatment. Significant tumor modules are indicated with arrows. (D) Tumor volume of B16 F10 flank tumors over a treatment period of 18 days. Treatments were administered at 3 days intervals starting on day 3. (E-F) AST (E) and ALT (F) levels of B16 F10 flank tumor bearing mice across each treatment group at the endpoint of the treatment (day 18). For the B16 F10 flank tumor treatment in (D)-(F), n=3 mice and the treatment consisted of agonistic CD137 antibody either in its free form or conjugated on EVs. Student's two-tailed t-test: ns – not significant, *P < 0.05, ***P < 0.001. Error bars represent standard deviation.

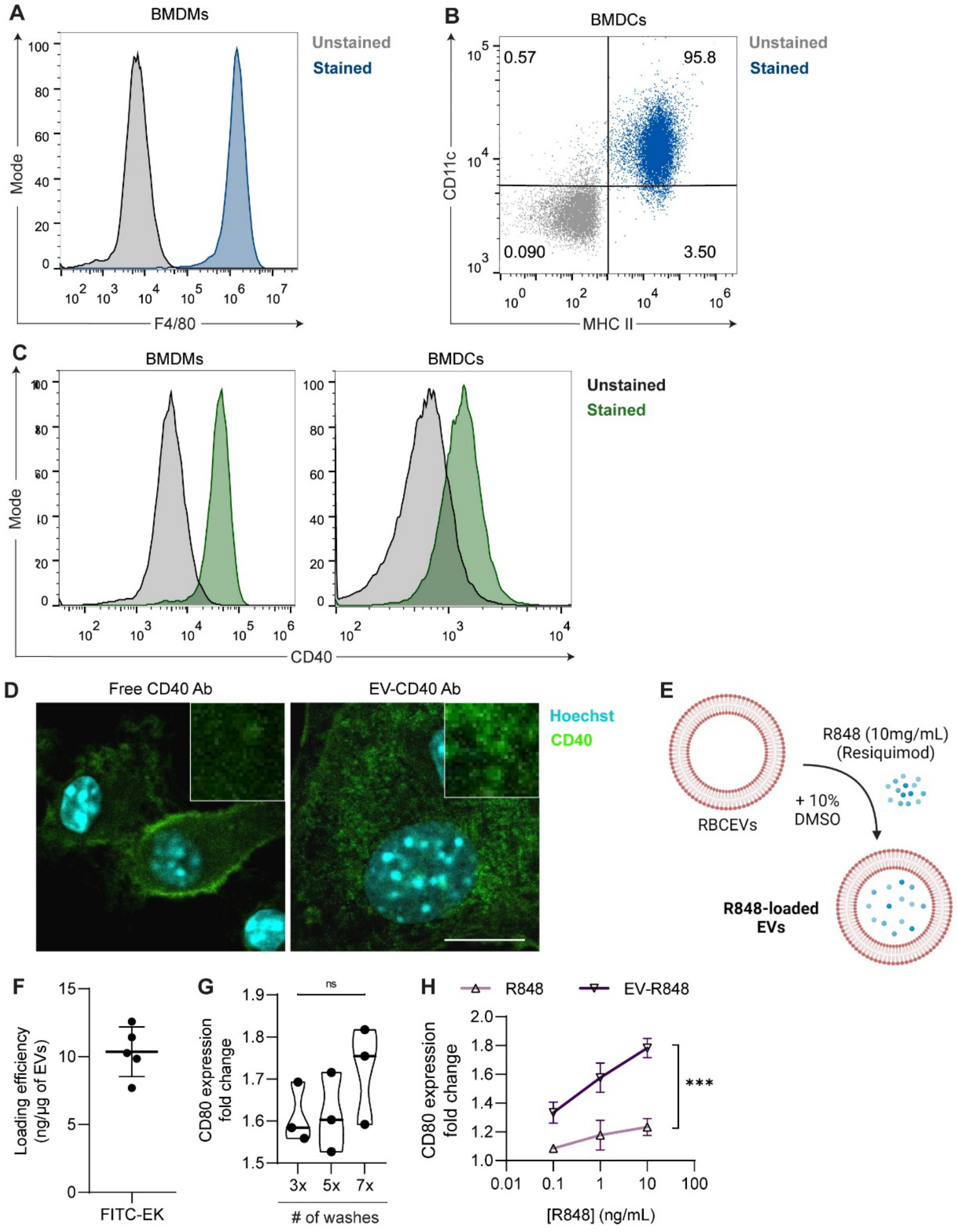

Figure S9| APC differentiation and R848 loading. (A) Flow cytometric analysis of F4/80 in murine BMDMs following differentiation and enrichment. (B) Flow cytometric analysis of DC markers CD11c and MHC II following differentiation and negative selection of murine BMDCs. (C) Flow cytometry histograms depicting CD40 expression in murine BMDMs and BMDCs. (D) High resolution image demonstrating distinct receptor clustering of CD40 in BMDMs following stimulation with free CD40 agonistic antibodies or EV-conjugated CD40 antibodies. Scale bar: 10 µm. (E) Protocol for loading R848 into EVs. EVs were co-incubated with R848 in the presence of 10% DMSO. (F) Quantity of R848 loaded per µg of EVs determined using a fluorescent probe. (G) CD80 expression fold change in RAW264.7 cells following incubation with R848-loaded EVs that were washed 3, 5 and 7 times respectively. (H) Relative increase in CD80 expression in RAW264.7 cells following treatment with increasing doses of free R848 or EV-loaded R848. For (F)-(H), each replicate was performed using EVs from individual donors. Student's two-tailed t-test: ns – not significant, *P < 0.05, ***P < 0.001. Error bars represent standard deviation.

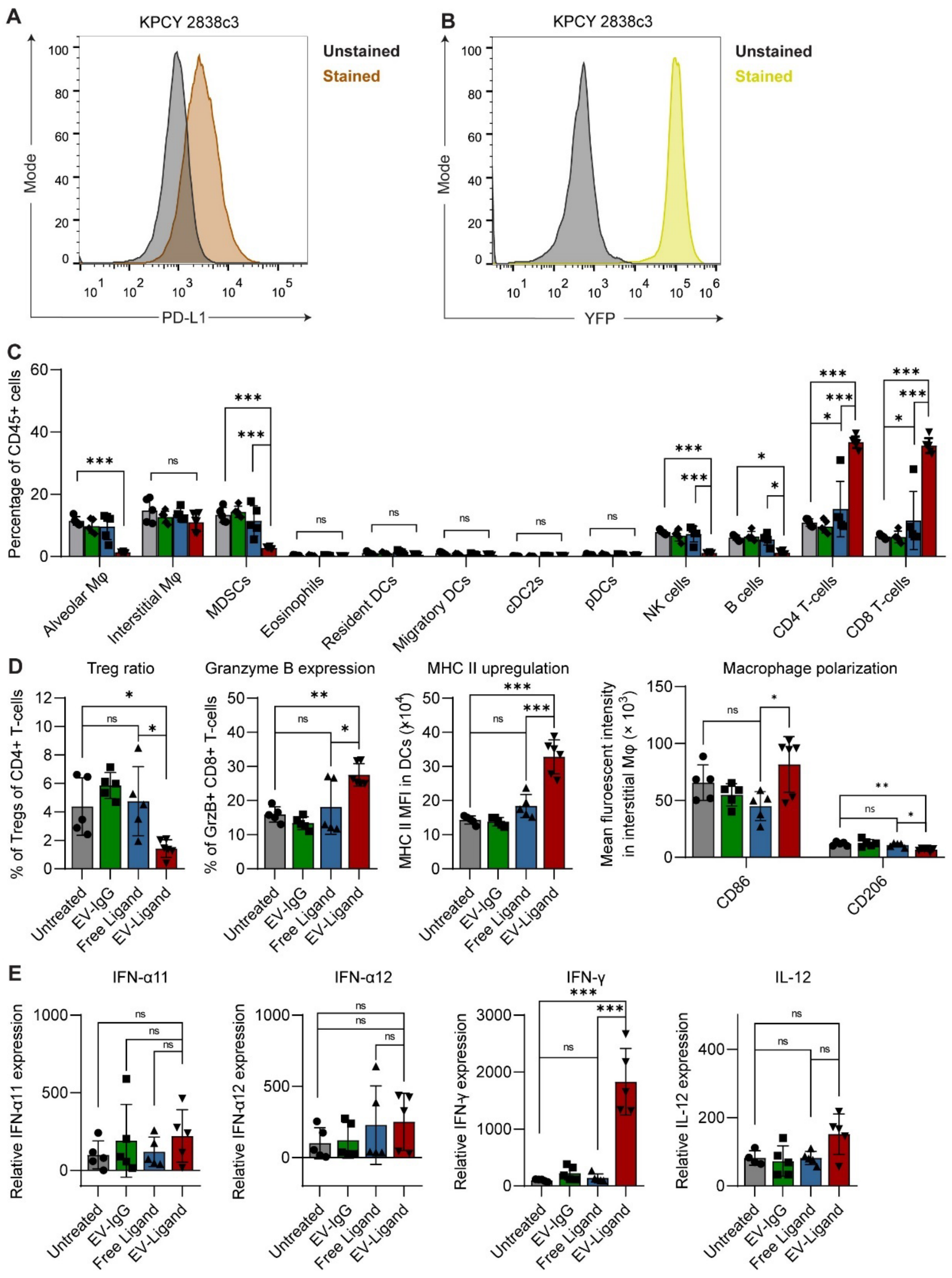

Figure S10| PDAC tumor immune composition analysis following treatment. (A) PD-L1 expression in KPCY 2838c3 cells determined using flow cytometry. (B) Expression of the fluorescent YFP reporter in KPCY 2838c3 cells determined using flow cytometry. (C) Composition of major immune cell subsets in the tumor-bearing lung 30 days post-treatment represented as a percentage of all CD45-positive cells. (D) Analysis of key immune cell subsets relevant to the generation of effective anti-cancer immune responses. (E) RT-qPCR analysis of the expression of various pro-inflammatory cytokines in lung extracts at the endpoint of the treatment. For (C)-(E) data was obtained from 5-6 mice. Student's two-tailed t-test: ns – not significant, *P < 0.05, ***P < 0.001. Error bars represent standard deviation.

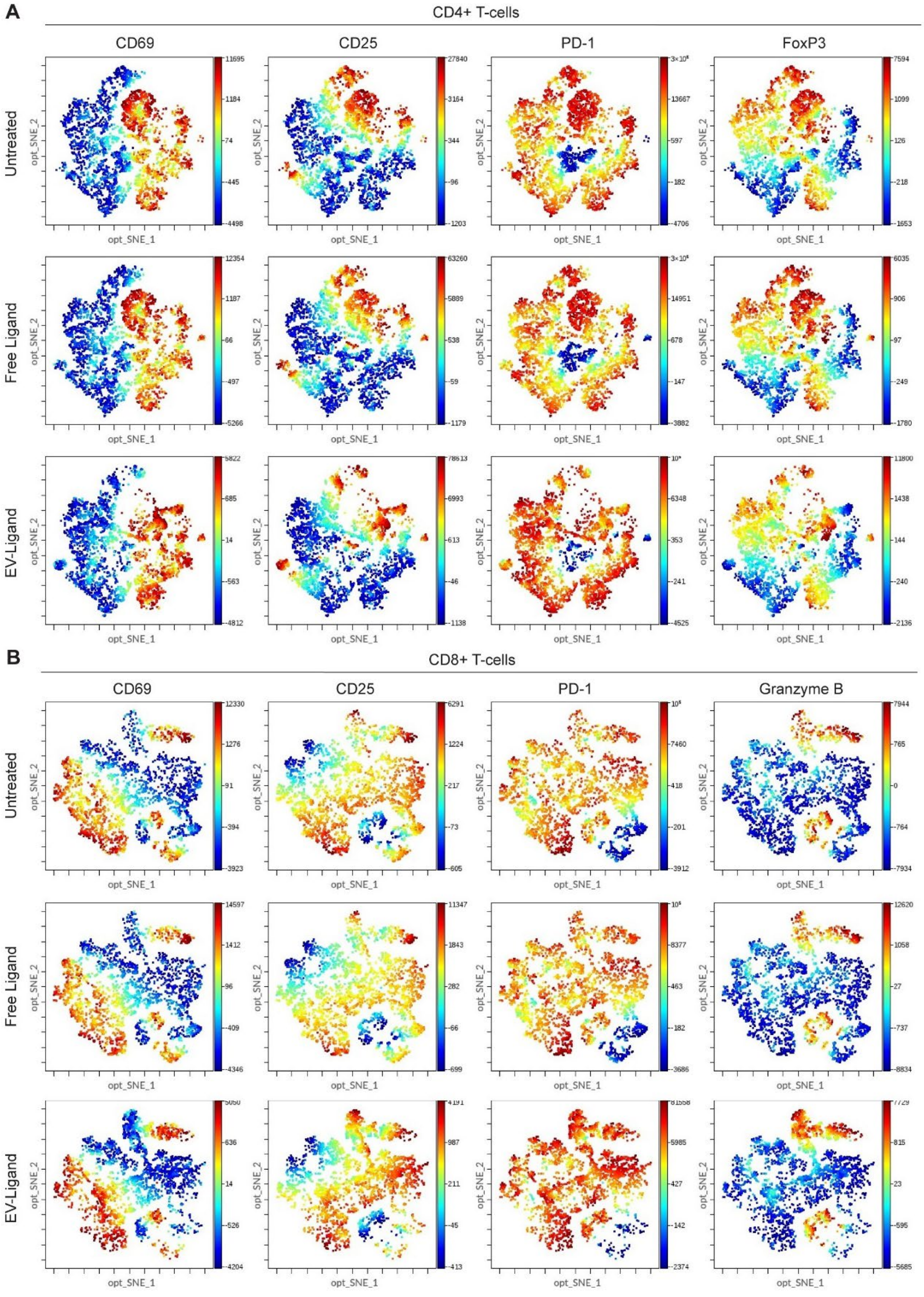

Figure S11| EV-Ligand treatments effectively remodel tumor infiltrating lymphocytes. (A) Detailed expression profiles of CD69, CD25, PD-1 and FoxP3 in CD4+ T-cells following administration with each of the indicated treatments. (B) In depth analysis of CD69, CD25, PD-1 and Granzyme B expression in CD8+ T-cells. For (A)-(B), T-cell subsets were subjected to a opt-SNE dimensionality reduction algorithm and the respective markers are indicated on the z-axis.

1 Dang, X. T. T. *et al.* Dendritic cell-targeted delivery of antigens using extracellular vesicles for anti-cancer immunotherapy. *Cell Proliferation* **n/a**, e13622 (2024). https://doi.org:https://doi.org/10.1111/cpr.13622

2 Jayasinghe, M. K. *et al.* Red Blood Cell-Derived Extracellular Vesicles Display Endogenous Antiviral Effects and Enhance the Efficacy of Antiviral Oligonucleotide Therapy. *ACS Nano* **17**, 21639-21661 (2023). https://doi.org:10.1021/acsnano.3c06803
